# Annelated N-heterocyclic carbene-derived organic redox system for consecutive visible-light photocatalysis

**DOI:** 10.1093/nsr/nwag408

**Published:** 2026-07-01

**Authors:** Yu-Jia Liu, Xin Li, Ji-Xin Man, Can Chen, Man Li, Jia-Ling Zhu, Ying-Feng Han

**Affiliations:** College of Chemistry, Zhengzhou University, Zhengzhou 450001, China; College of Chemistry, Zhengzhou University, Zhengzhou 450001, China; College of Chemistry and Materials Science, Northwest University, Xi’an 710127, China; College of Chemistry and Materials Science, Northwest University, Xi’an 710127, China; College of Chemistry and Materials Science, Northwest University, Xi’an 710127, China; College of Chemistry and Materials Science, Northwest University, Xi’an 710127, China; College of Chemistry, Zhengzhou University, Zhengzhou 450001, China; College of Chemistry and Materials Science, Northwest University, Xi’an 710127, China

**Keywords:** N-heterocyclic carbene, organic redox system, consecutive photoinduced electron transfer, photocatalysis, radical

## Abstract

Electron transfer is a fundamental process ubiquitous in both natural and artificial systems. The construction of organic redox systems serves as an ideal platform to explore this mechanism. The interconversion between different electronic structures endows them with unique photoelectric properties, enabling their promising applications in fields such as photocatalysis, organic batteries and electrochromic materials. Herein, we present an organic redox system derived from bis(imino)acenaphthene-supported N-heterocyclic carbene, which exhibits two reversible and well-separated redox processes. The different oxidation states could be isolated and fully characterized, including X-ray diffraction. Moreover, the radical cation exhibits strong absorption in the visible-light region and can be easily generated through photoinduction, fulfilling the prerequisites for consecutive photoinduced electron transfer. Therefore, organic redox system can function as a photocatalyst in the reduction of aryl halides. This strategy expands the variety of organic redox systems and provides a novel approach to the development of photocatalysts.

## Teaser

The bis(imino)acenaphthene-supported N-heterocyclic carbene with a p-phenylene skeleton constructs a three-state redox system and enables the activation of C(sp2)–X via consecutive photoinduced electron transfer.

## INTRODUCTION

Organic redox systems, characterized by multiple oxidation states and interconvertibility between distinct electronic states, play a crucial role in understanding electron transfer processes and hold broad application prospects in fields such as organic electronics and energy storage [1–3]. The alteration of the electronic structure during electron transfer can shift the absorption wavelength to the visible-light or even the near-infrared regions [4–7]. This unique photoelectric property enables the mimicking of the Z scheme of natural photosynthesis using the consecutive photoinduced electron transfer (ConPET) mechanism [8–11], further expanding the redox window of the photocatalyst, thereby enabling the activation of inert chemical bonds and the transformation of complicated molecules [12–14].

Thiele’s hydrocarbon (**TH**), a classical Kekulé diradicaloid with a *p*-phenylene skeleton, was first prepared shortly after Gomberg’s synthesis of the triphenylmethyl radical [15–18]. **TH** can undergo continuous oxidation [19], making it a promising candidate for organic redox systems. Nevertheless, the extreme instability of its radical cation (*t*_1/2_ = 3 s) significantly constrains the exploration of its redox properties and applications [19]. Over the past years, N-heterocyclic carbenes (NHCs) have shown remarkable effectiveness in the supramolecular self-assembly [20–22], small molecule activation [23,24] and stabilization of various paramagnetic species [25–27]. In particular, classical NHCs (1,3-bis(2,6-diisopropylphenyl)imidazole-2-ylidene, IPr; 1,3-bis(2,6-diisopropylphenyl)imidazolidin-2-ylidene, SIPr) [28,29], cyclic alkyl amino carbene (CAAC) [30] and *N,N'*-diamidocarbene [31] have been used to synthesize stable analogs of **TH** (Fig. 1a). However, the two redox potentials of these symmetric systems are close or overlap, making it impossible to isolate the corresponding radical cations. Thus, it is difficult for them to serve as organic redox systems to study the stepwise electron transfer.

**Figure 1.**
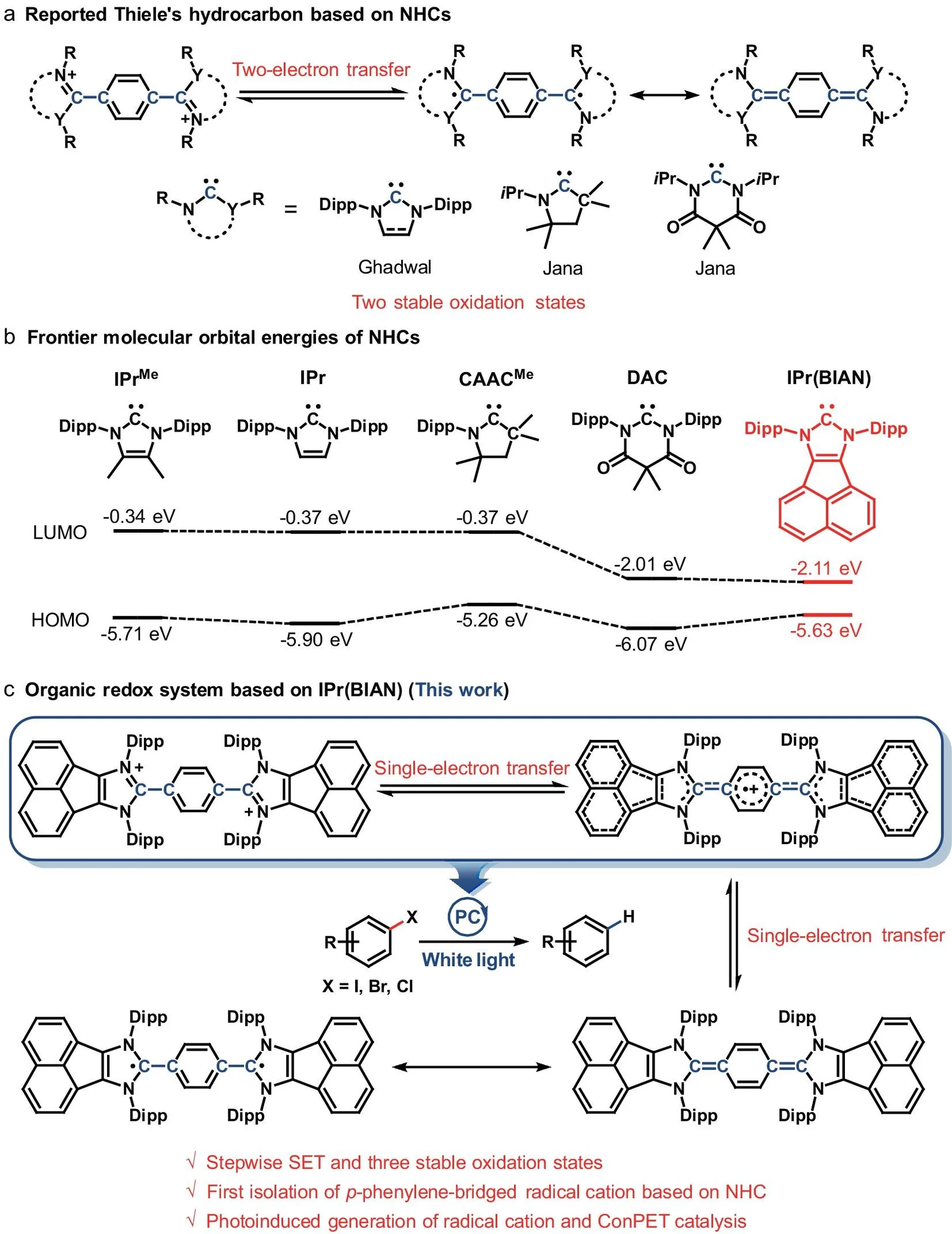
(a) Reported Thiele’s hydrocarbon based on NHCs. (b) The frontier orbital energies of NHCs calculated at the B3LYP/6-311G(d) level. (c) This work: organic redox system based on IPr(BIAN) (Dipp = 2,6-diisopropylphenyl).

Bis(imino)acenaphthene-supported NHC (IPr(BIAN)) is frequently used in transition-metal catalysis [32–36]. Recently, we have expanded its application, successfully isolating carbon-centered monoradicals and Breslow enolates [37,38]. IPr(BIAN) constitutes the basic imidazolylidene ring system fused with an additional *π*-system. Such a large rigid planar framework structure endows IPr(BIAN) with a narrower HOMO-LUMO gap (HOMO: highest occupied molecular orbital; LUMO: lowest unoccupied molecular orbital) and lower LUMO level compared to classical NHCs and CAACs, which can enhance electronic coupling and facilitate the stabilization of unpaired electrons (Fig. 1b). To leverage these characteristics, we replaced the Ph_2_C units at both ends of the **TH** with IPr(BIAN) and synthesized an organic redox system based on a bis(annelated imidazolium) salt (Fig. 1c). Consistent with our expectations, this system showed three stable oxidation states. The corresponding radical cation was successfully isolated for applications in the analysis of structural and property changes during electron transfer and as a photocatalyst for consecutive visible-light-driven photocatalytic reactions.

## RESULTS AND DISCUSSION

The direct C2-arylation of 1,4-diiodobenzene with IPr(BIAN) mediated by a catalytic amount of Pd_2_(dba)_3_ afforded the corresponding dication [**1a**^2+^][I^−^]_2_. Its structure was characterized via nuclear magnetic resonance spectroscopy (NMR) and high-resolution mass spectrometry (Figs S1‒S3). A cyclic voltammogram (CV) of [**1a**^2+^][I^−^]_2_ in MeCN/Bu_4_NPF_6_ reveals two reversible one-electron redox processes (*∆E*_1_^red^ = 67 mV; *∆E*_2_^red^ = 63 mV), showing that electron transfer proceeds in a stepwise manner (Fig. 2a). This was further confirmed via differential pulse voltammetry. The first redox process at *E*_1/2_ = −1.37 V versus Fc/Fc^+^ was assigned to the formation of the radical cation, followed by a second reduction at *E*_1/2_ = −1.67 V, generating the neutral diradicaloid. In contrast, previously reported CAAC-based **TH** exhibits only one reversible redox process [30]. Although the classical NHC-based **TH** (*∆E* = 150 mV for SIPr; *∆E* = 250 mV for IPr) shows two redox processes, they are less well-separated than in [**1a**^2+^][I^−^]_2_ (*∆E* = 300 mV) [28,29]. This indicates that IPr(BIAN)-substitution increases electronic coupling and confers a certain degree of thermodynamic stability to the radical cation.

**Figure 2.**
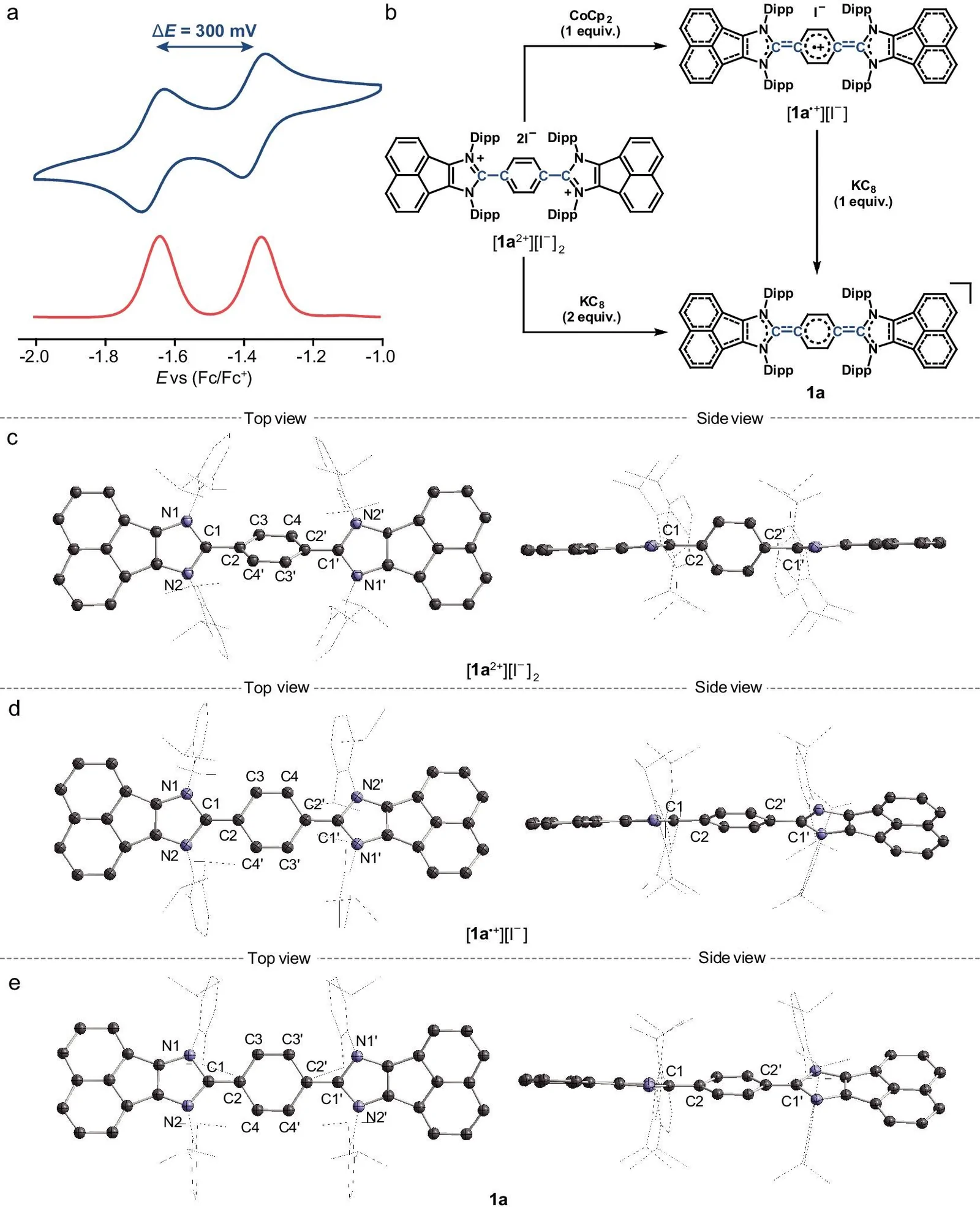
(a) CV (top) and differential pulse voltammogram (bottom) of [**1a**^2+^][I^−^]_2_ (Bu_4_NPF_6_ in MeCN, 100 mV s^−1^ vs Fc/Fc^+^). (b) Synthesis of radical cation [**1a**$^{\bullet +}$][I^−^] and diradicaloid **1a**. Solid-state molecular structures of (c) [**1a**^2+^][I^−^]_2_, (d) [**1a**$^{\bullet +}$][I^−^] and (e) **1a**. For clarity, all H atoms and counter anions are omitted.

Thereafter, dication [**1a**^2+^][I^−^]_2_ was chemically reduced with one equivalent of cobaltocene in CH_2_Cl_2_, and the obtained radical cation [**1a**$^{\bullet +}$][I^−^] was isolated as an NMR-silent dark purple solid (Figs S4 and S5). When two equivalents of KC_8_ were used, diradicaloid **1a** was formed and subsequently isolated as a purple-red solid (Fig. 2b). The diradicaloid **1a** can also be synthesized via the one-electron reduction of radical cation [**1a**$^{\bullet +}$][I^−^] using KC_8_. [**1a**$^{\bullet +}$][I^−^] and **1a** are air- and moisture-sensitive but stable under an inert gas atmosphere.

The solid-state structures of [**1a**$^{\bullet +}$][I^−^] (Fig. 2d) and **1a** (Fig. 2e) reveal that upon the reduction of [**1a**^2+^][I^−^]_2_ (Fig. 2c), the dihedral angle between the central phenylene ring and the peripheral NHC ligand substantially changes (61.06° in [**1a**^2+^][I^−^]_2_ to 14.08° in [**1a**$^{\bullet +}$][I^−^] and 14.76° in **1a**). The C1‒C2 bond length in [**1a**$^{\bullet +}$][I^−^] (1.414(9) Å) is shorter than that in [**1a**^2+^][I^−^]_2_ (1.465(4) Å), presenting a symmetrical structure, which indicates significant electronic coupling between the two redox sites (Table S1). Upon further reduction to **1a**, the C1‒C2 bond length decreases to 1.376(5) Å, which is comparable to that observed in classical NHC-supported diradicaloids (1.371 Å for SIPr and 1.375 Å for IPr) but shorter than that in **TH** (1.381 Å) [31,32,42]. The C‒C bond lengths of the central phenylene ring in **1a** exhibit greater bond length alternation (BLA = 0.10 Å) than those of [**1a**^2+^][I^−^]_2_ (BLA = 0.01 Å) and [**1a**$^{\bullet +}$][I^−^] (BLA = 0.07 Å), indicating a transition toward a quinoidal structure. The structural changes among the three different oxidation states are consistent with stepwise reduction processes, explicitly demonstrating the gradual enhancement of the delocalization of the unpaired electron over the *π*-conjugated system.

X-band electron paramagnetic resonance (EPR) spectra of [**1a**$^{\bullet +}$][I^−^] (which are in good agreement with simulation results) confirm the presence of an unpaired electron centered at *g* = 2.0032, which is hyperfinely coupled with all four nitrogen atoms, hinting at the fully delocalized nature across the *π*-conjugated system (Fig. 3a). Diradicaloid **1a** could conceivably exist in any of the three possible forms: an open-shell diradical that could have either a singlet or a triplet ground state, and a ‘quinoidal’ closed-shell singlet. At room temperature, the ^1^H NMR spectrum of **1a** displayed virtually no proton signals (Figs S6 and S7), indicating a substantial population of the triplet state under these conditions. Lowering the temperature likewise yielded no observable signals in the ¹H NMR spectrum. To further elucidate the electronic structure of **1a**, variable-temperature EPR measurements were conducted over the range of 190–290 K, the results revealed an increase in EPR signal intensity with rising temperature (Fig. 3b). Moreover, fitting the temperature-dependent double-integral intensity to the Bleaney–Bowers equation afforded an estimated singlet–triplet energy gap (*∆E*_ST_) of −1.31 kcal mol^−1^ (Fig. S65). At the same time, a half-field signal is observed in the solid state at 290 K. These findings indicate a thermally excited triplet state population at room temperature, which strikingly contrasts with the spin states and characteristics of **TH** [39]. **TH** is in a closed-shell singlet state as its ground state at room or lower temperatures, with an inaccessible triplet state [40].

**Figure 3.**
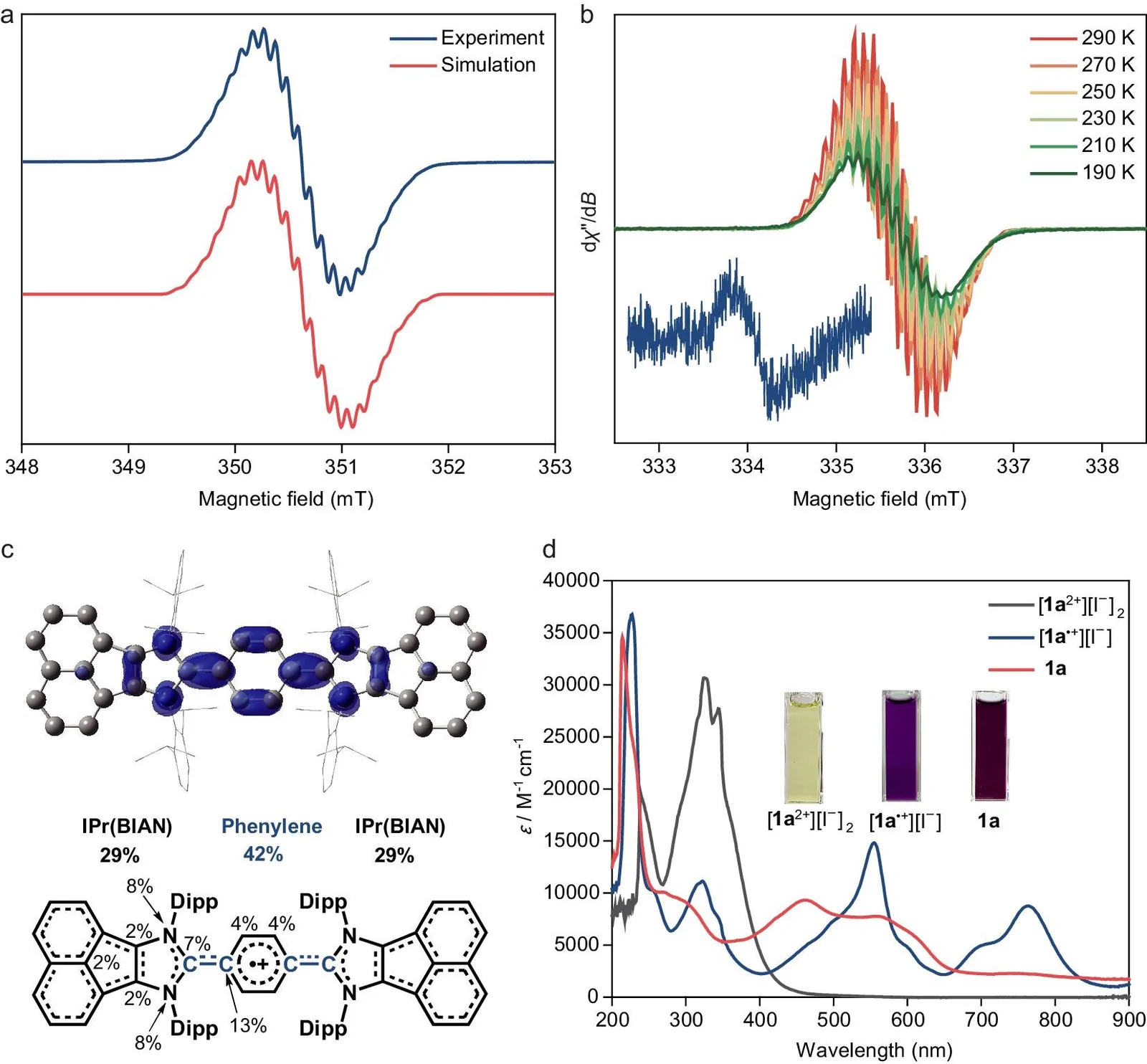
(a) Experimental (blue) and simulated (red) EPR spectra of [**1a**$^{\bullet +}$][I^−^] in CH_2_Cl_2_ at room temperature. (b) Variable-temperature EPR spectra of **1a** in 2-methyltetrahydrofuran between 190 and 290 K with the inset showing the half-field signal in the solid state at 290 K. (c) Calculated spin densities (isovalue 0.0015 a.u.) and Mulliken spin density values at the UB3LYP/6-311G(d) theoretical level of [**1a**$^{\bullet +}$][I^−^]. Hydrogen atoms are omitted for clarity. (d) Ultraviolet-visible (UV-vis) spectra of [**1a**^2+^][I^−^]_2_, [**1a**$^{\bullet +}$][I^−^] and **1a** in tetrahydrofuran (THF) at room temperature (*c* = 10^−5^ M).

Afterward, quantum chemical calculations were performed to investigate the spin distribution and ground-state electronic structures (Figs S76‒S83 and Tables S8‒S12). The Mulliken spin density of [**1a**$^{\bullet +}$][I^−^] suggests that a relatively significant unpaired electron density (13%) at the two ends of the *π*-conjugated system is concentrated at the C2 and C2′ centers. Approximately 42% of the spin density is on the central phenylene ring, whereas the remaining 58% is distributed across the two IPr(BIAN) moieties (Fig. 3c). This shows that the unpaired electron is well distributed between the two redox centers of the molecule, consistent with the EPR results. For **1a**, the theoretical *∆E*_ST_ is −5.03 kcal mol^−1^, indicating a closed-shell singlet ground state, which aligns with the experimental value obtained through variable-temperature EPR. Additionally, no indicators of a low-energy open-shell singlet state are observed.

Nucleus-independent chemical shift (NICS) calculations were employed to further clarify the changes in the electronic structure during stepwise reduction (Table S9). The results indicate that the central phenylene ring of [**1a**^2+^][I^−^]_2_ (NICS(1)_ZZ_ = −23.42) and [**1a**$^{\bullet +}$][I^−^] (NICS(1)_ZZ_ = −2.69) retains the aromatic character, with a notable decrease in aromaticity observed upon one-electron reduction. In contrast, **1a** exhibits the anti-aromatic character (NICS(1)_ZZ_ = 10.12). These data, along with the crystal structures and EPR spectra, reinforce the idea that electron delocalization occurs across the *π*-conjugated system when [**1a**^2+^][I^−^]_2_ is converted into [**1a**$^{\bullet +}$][I^−^] or **1a** and support the quinoidal character of the central phenylene ring in **1a**. The description of **1a** is also supported by the HOMO morphology, which shows the alternating bonding/antibonding character of the C‒C bonds in the central ring (Fig. S78).

The UV-vis absorption spectra were recorded for [**1a**^2+^][I^−^]_2_, [**1a**$^{\bullet +}$][I^−^] and **1a** in THF (Fig. 3d). The absorption band of [**1a**^2+^][I^−^]_2_ is located in the UV region with a main peak around 326 nm. According to the results of time-dependent density functional theory calculations, this peak corresponds to the HOMO→LUMO transition. Radical cation [**1a**$^{\bullet +}$][I^−^] shows a strong absorption peak in the visible region at 555 nm, involving the HOMO(β)→SUMO (SUMO: singly unoccupied molecular orbital) transition, along with two broad Gaussian-shaped absorption bands at 702 and 762 nm attributed to the intervalence charge transfer from the radical cation. For **1a**, the broad absorption peak in the visible region at 462 nm was attributed to a transition from the bonding orbitals on the central ring (HOMO) to the antibonding orbitals (LUMO + 2). All three oxidation states not only feature strong UV-vis absorption but are also stable and separable, implying potential applications in visible-light photocatalysis.

Aryl halides are versatile building blocks in organic synthesis that are widely utilized as nucleophilic reagents in aromatic substitution reactions, precursors for the synthesis of organometallic reagents and electrophiles in transition-metal catalysis [41–43]. Nevertheless, because of the high energy barrier, and thereby a low reduction potential (*E*_red_ ∼ −1.9 V vs. saturated calomel electrode) [44], the activation of aryl halides to cleave the carbon‒halogen bond at room temperature is challenging. In this light, we speculate that owing to its reversible redox capability and favorable photophysical properties, [**1a**^2+^][I^−^]_2_ could activate the C(*sp*^2^)‒X bonds of less reactive aryl halides through the ConPET mechanism [8].

We used 4-bromobenzaldehyde as a standard substrate and optimized the reaction conditions by irradiating a mixture of substrate, photocatalyst [**1a**^2+^][I^−^]_2_ and electron donor with white light (Tables S13‒S16). Different amines were tested as electron donors, with triethylamine (Et_3_N) yielding the best results (entry 8, Table S13). A slightly better gas chromatography (GC) yield is obtained for MeCN compared to other solvents within the same time period (entry 4, Table S14). Additionally, reducing the photocatalyst loading from 5 mol% to 2 mol% afforded the highest photoreduction yield of 54% (entry 4, Table S15). However, further decreasing the amount of Et_3_N led to a reduction in the yield (Table S16). In addition, no obvious effect of counteranions was observed (Table S17). Blank reactions without a photocatalyst or electron donor yielded only traces of the reduced product, and the reaction did not proceed in the dark (entries 1–3, Table S18). These results confirm that photocatalyst [**1a**^2+^][I^−^]_2_, electron donor Et_3_N and light irradiation are essential for the photoreduction reaction. In addition, light on/off experiments demonstrated that the formation and cessation of the product were strictly synchronized with switching the irradiation on or off, ruling out a radical chain mechanism (Fig. S84).

Using the optimized reaction conditions, the influence of carbene skeletons and substituent effects on photocatalytic efficiency was systematically investigated through control experiments (Table 1). We compared the photocatalytic efficiency of [**1a**^2+^][I^−^]_2_, [**1b**^2+^][I^−^]_2_ (based on IPr) and [**1c**^2+^][I^−^]_2_ (based on SIPr) firstly. The results demonstrate that [**1a**^2+^][I^−^]_2_ exhibits the highest catalytic performance (compare entry 1 with entries 2 and 3), whereas [**1c**^2+^][I^−^]_2_ displays the lowest efficiency, followed by [**1b**^2+^][I^−^]_2_. These observations indicate that compared with the classical NHCs, the introduction of IPr(BIAN) is beneficial for improving the photocatalytic activity of this system. Consistent with theoretical calculations, IPr(BIAN) exhibits a lower LUMO level, which enhances the stability of the radical cation and thereby may facilitate the ConPET process. Moreover, its narrower HOMO–LUMO gap further promotes photoexcitation. Then, structural modification of IPr(BIAN) through the introduction of different substituents enabled the successful synthesis of photocatalysts [**1d**^2+^][I^−^]_2_ and [**1e**^2+^][I^−^]_2_ (for detailed synthesis and characterization, see the Supplementary data). However, both cyano- and methoxy-substituted photocatalysts resulted in reduced yields (compare entry 1 with entries 4 and 5), which may be attributed to the reduced separation between the two redox processes ([**1d**^2+^][I^−^]_2_ exhibits two close-lying one-electron waves; *∆E* = 280 mV for [**1e**^2+^][I^−^]_2_; Figs S48−S51). Introducing the methoxy group into the IPr(BIAN) skeleton raises the LUMO level, destabilizing the radical cation (Fig. S67). In contrast, while the cyano group lowers the LUMO level, its high reactivity also readily leads to deactivation of the catalyst, reducing the yield [45]. Additionally, asymmetric photocatalysts [**1f**^2+^][I^−^]_2_ and [**1g**^2+^][I^−^]_2_ were constructed by combining IPr(BIAN) with classical NHCs (synthetic details and characterization are provided in the Supplementary data). Notably, the introduction of IPr(BIAN) leads to a marked improvement in catalytic efficiency compared to symmetric NHC-based catalysts [**1b**^2+^][I^−^]_2_ and [**1c**^2+^][I^−^]_2_, although their performance remains inferior to that of [**1a**^2+^][I^−^]_2_ (entries 1–3, 6 and 7). Surprisingly, when [**1f**^2+^][I^−^]_2_ photocatalyzed the reduction of 4-bromobenzaldehyde, only trace amounts of the product were detected (entry 6). The results further confirm that IPr(BIAN) plays a key role in enhancing photocatalytic efficiency.

**Table 1.**
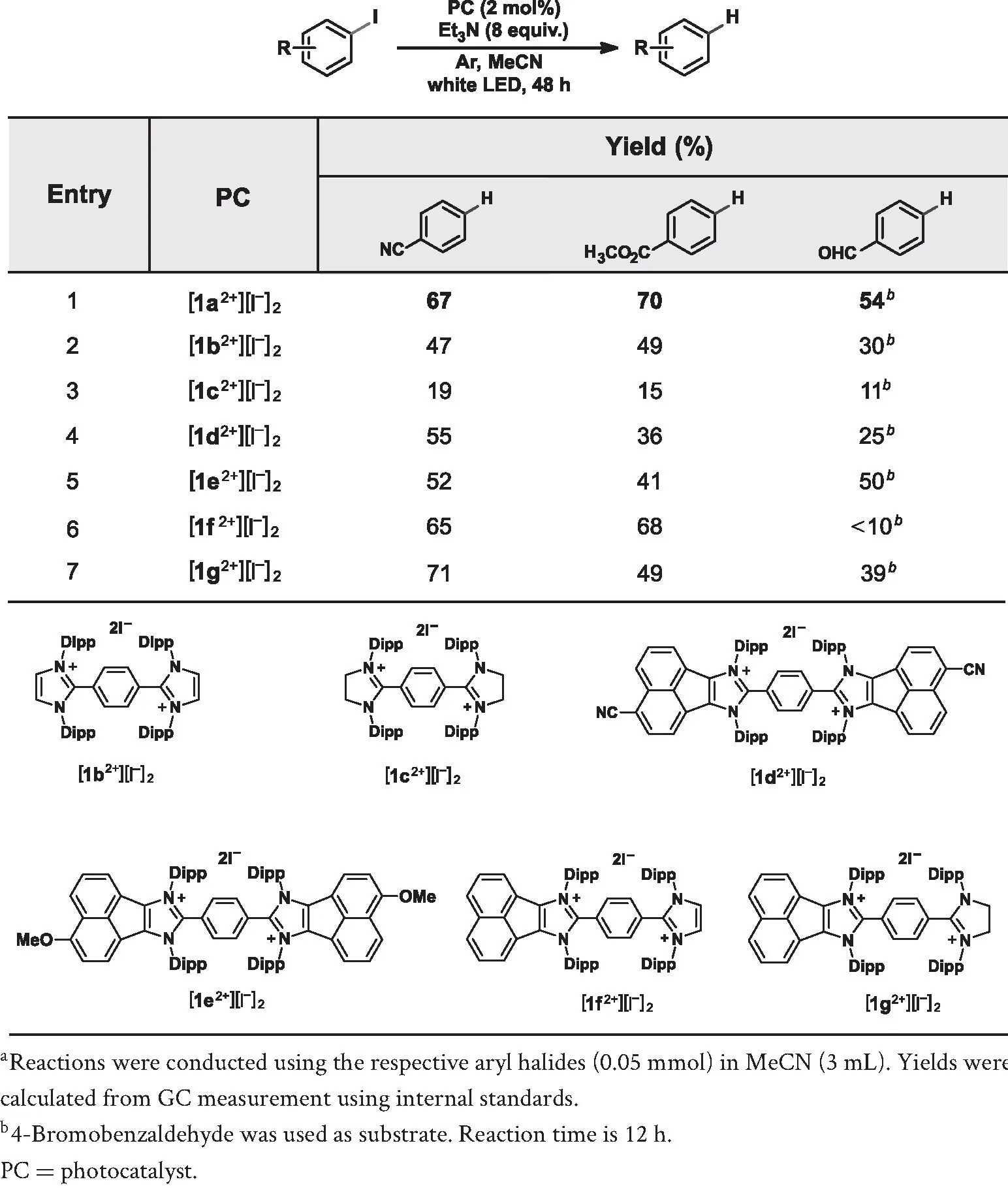
Control experiments.^a^

By selecting [**1a**^2+^][I^−^]_2_ with the best comprehensive efficiency, we determined the substrate scope of different substituted aryl halides (Scheme 1a). Under standard reaction conditions, various substituted aryl iodides and aryl bromides afforded moderate-to-excellent yields of the photoreduction products. Notably, when the substituent is an electron-donating group, the reduction potential of aryl halide is more negative [46], but the reduction products are still obtained in moderate yields. Further ^1^H NMR analysis confirmed that the limited yield was primarily due to self-coupling of the aryl radical and incomplete substrate conversion (Figs S85 and S86). We also attempted to substitute aryl chlorides, which are readily available and relatively inexpensive. This class of compounds exhibits low reactivity because of their low reduction potentials and the high energy barrier for C(*sp*^2^)‒Cl bond activation (PhCl at ∼97 kcal mol^−1^) [47]. Fortunately, when the electron-withdrawing group is a *para* or *ortho* substituent, the photoreduction products are obtained in moderate yields. Subsequently, the photoreduction reaction was further conducted on a larger scale, and the isolated yields were determined. These results indicated that the catalytic system maintained moderate-to-excellent catalytic efficiency. Moreover, under the same conditions, the classical metal-based photocatalysts Ru(bpy)_3_Cl_2_ and *fac*-Ir(ppy)_3_ exhibited lower catalytic efficiency than [**1a**^2+^][I^−^]_2_, particularly in the case of aryl bromides (Table S19).

**Scheme 1.**
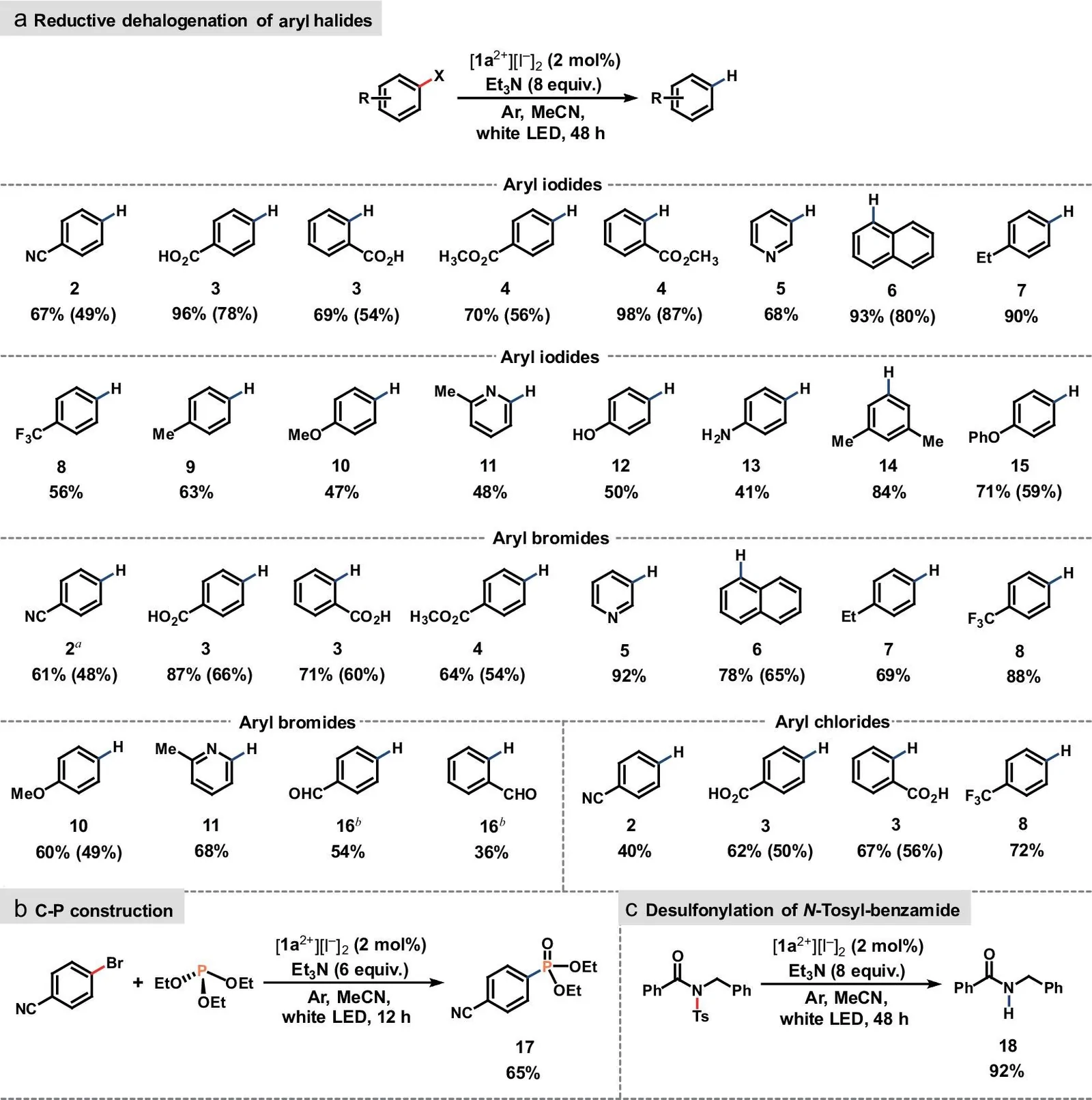
(a) Substrate scope for the photoreduction of aryl halides. All reactions were conducted using the respective aryl halides (0.05 mmol) in MeCN (3 mL). Yields were calculated from GC measurement. Isolated yields of aryl halides (0.5 mmol) in MeCN (10 mL) are given in parentheses. (b) Catalytic C–P bond forming reaction and (c) reductive cleavage of a *N*-tosyl group. Yields were calculated from NMR measurement. ^a^An additional 2 equiv. KBF_4_ was used. ^b^Reaction time is 12 h.

Based on the relevant literature and experimental results, we proposed a reasonable reaction mechanism (Fig. 4a) [8–11,48–50]. To verify this mechanism, we monitored the generation and quenching of radical cation [**1a**$^{\bullet +}$][I^−^] under light irradiation through UV-vis spectroscopy. In the presence of Et_3_N as an electron donor, after irradiation with white light, the color of the solution changes from light yellow to purple, and a new absorption peak at 555 nm appears, confirming the formation of [**1a**$^{\bullet +}$][I^−^] (Fig. 4c). The formation of diradicaloid **1a** is not detected under these reaction conditions (Figs 3d and 4c). Upon the exposure of the light-irradiated mixture to air, [**1a**$^{\bullet +}$][I^−^] gradually quenches back to [**1a**^2+^][I^−^]_2_ (Fig. 4d). These results confirm that the electron transfer from Et_3_N to [**1a**^2+^][I^−^]_2_ requires photoexcitation. Subsequently, in the blank experiments, no reduction occurred upon the addition of 4-bromobenzaldehyde to chemically generated [**1a**$^{\bullet +}$][I^−^]. However, after exposure to white-light irradiation, the reduced product was detected, indicating the occurrence of a second photoexcitation (entries 4 and 5, Table S18). Furthermore, the near quadratic relation between the yield and the irradiation density provides kinetic evidence for an overall two-photon excitation process (Fig. S89).

**Figure 4.**
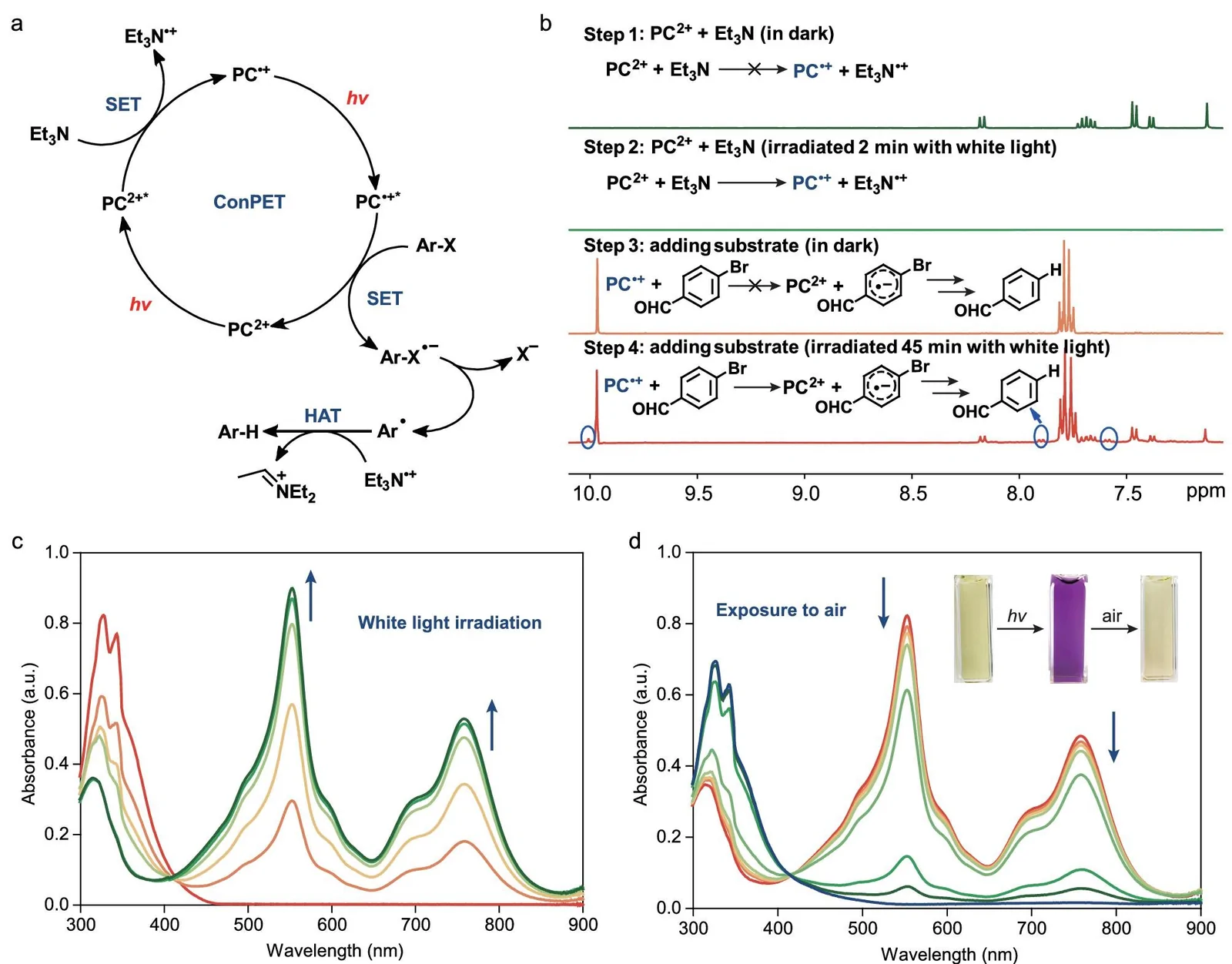
(a) Plausible photoreduction mechanism of aryl halides through the ConPET process. (b) ^1^H NMR spectroscopic evidence supporting the proposed ConPET process. (c) In the presence of Et_3_N, [**1a**^2+^][I^−^]_2_ is photoexcited (white light) to form [**1a**$^{\bullet +}$][I^−^] (*c* = 4 × 10^−6^ M). (d) Regeneration of [**1a**^2+^][I^−^]_2_ upon exposure to air.

Further ^1^H NMR measurements provide direct evidence for the ConPET mechanism (Fig. 4b). The NMR signal vanishes upon the white-light irradiation of the mixture in the NMR tube, indicating the generation of paramagnetic species [**1a**$^{\bullet +}$][I^−^] (steps 1 and 2). The addition of the substrate does not affect [**1a**$^{\bullet +}$][I^−^] in the dark (step 3). However, upon light irradiation, [**1a**$^{\bullet +}$][I^−^] is quenched back to [**1a**^2+^][I^−^]_2_, and the benzaldehyde product is detected (step 4). Thus, all results support the proposed catalytic cycle. Upon light excitation, [**1a**^2+^][I^−^]_2_ undergoes single-electron transfer with Et_3_N to generate [**1a**$^{\bullet +}$][I^−^] and the Et_3_N$^{\bullet +}$ radical cation (Figs S90 and S91). Then, photoexcited [**1a**$^{\bullet +}$][I^−^] exhibits a reduction potential of −2.87 V vs. Fc/Fc^+^, sufficiently low to engage in electron transfer with the aryl radical precursor, leading to bond cleavage and the formation of an aryl radical, while simultaneously forming [**1a**^2+^][I^−^]_2_ in the ground state. Finally, the aryl radical abstracts a hydrogen atom from Et_3_N$^{\bullet +}$ to generate the desired reduction products.

After successful examination of the photoreduction reaction, we decided to extend this radical photocatalysis to other reactions. We utilized phosphite as a reaction partner that delivered the C‒P coupling product with good catalytic efficiency (product **17**, Scheme 1b). A plausible mechanistic pathway is illustrated in Fig. S93. Phosphite serves as a well-established radical-trapping agent and readily couples with an aryl radical to form a C–P bond, affording a transient phosphoranyl radical intermediate. This intermediate subsequently releases an ethyl radical, thereby furnishing the corresponding aryl phosphonate product. Furthermore, similar to aryl halides, the desulfonylation reaction of *N*-tosyl-benzamide also proceeds via the ConPET mechanism, affording the expected unprotected amide in a 92% yield (product **18**, Scheme 1c and Fig. S94).

## CONCLUSION

In summary, we report the first isolation of an IPr(BIAN)-based *p*-phenylene-bridged radical cation, obtaining an organic redox system with three oxidation states, which can undergo stepwise and reversible single-electron transfer. The results of single-crystal X-ray diffraction and theoretical calculations revealed that the stepwise single-electron reduction is accompanied by a considerable contraction of the C1‒C2 bond length (from 1.465 to 1.376 Å) and an increase in electron delocalization. Notably, the prepared diradicaloid is in a thermally excited triplet state at room temperature, in sharp contrast to **TH**. Additionally, the radical cation is stable and exhibits strong absorption in the UV-vis region, which can be generated through photoinduction and activate inert C(*sp*^2^)‒X bonds through the ConPET mechanism with good catalytic yields. Therefore, the current strategy not only facilitates the expansion of the variety of organic molecules with multiple redox states but also enables the use of such molecules as promising organic photocatalysts.

## Supplementary Material

nwag408_Supplemental_Files

## Data Availability

Experimental details for all new compounds with synthesis, characterization, spectra (^1^H NMR, ^13^C{^1^H} NMR, MS, CV, UV-vis and EPR) and DFT computation details are provided in the Supplementary data.
