# Supplementary material for: Annelated N-heterocyclic carbene-derived organic redox system for consecutive visible-light photocatalysis
Source: Natl Sci Rev. 2026 Jul 1;13(19):nwag408. doi: 10.1093/nsr/nwag408 (PMC13656323; doi:10.1093/nsr/nwag408)
Supplement: Supplementary file 1 [file nwag408_supplemental_files.zip › Supporting_Information-MS-2026-1000.R1.pdf]

Supporting Information for

Annelated N-heterocyclic carbene-derived organic redox system for consecutive visible-light photocatalysis

Yu-Jia Liu[1,†], Xin Li[1,†], Ji-Xin Man[2], Can Chen[2], Man Li[2], Jia-Ling Zhu[2] and Ying-Feng Han[1,2,*]

[1]College of Chemistry, Zhengzhou University, Zhengzhou 450001, China and [2]College of Chemistry and Materials Science, Northwest University, Xi'an 710127, China

***Corresponding author**. E-mail: yfhan@nwu.edu.cn

[†]Equally contributed to this work.

Table of Contents

1. Experimental section S2

1.1 Materials and methods S2

1.2 Synthesis and characterization of [**1a**$^{2+}$][I^-]$_2$, [**1a**$^{2+}$][BF_4^-]$_2$, [**1a**$^{2+}$][PF_6^-]$_2$, [**1a**$^{\bullet+}$][I^-] and **1a** S2

1.3 Synthesis and characterization of [**1d**$^{2+}$][I^-]$_2$, [**1e**$^{2+}$][I^-]$_2$, [**1f**$^{2+}$][I^-]$_2$ and [**1g**$^{2+}$][I^-]$_2$ S12

2. Cyclic voltammetry S32

3. Crystallographic details S37

4. EPR spectra S47

5. Computational details S49

6. Photocatalysis S62

6.1 General procedure for the photoreduction S62

6.2 Procedure for carbon-phosphorus coupling S62

6.3 Optimization of photoreduction reaction conditions S63

6.4 Calculation of the excited-state redox potential S65

6.5 Mechanistic experiments S65

6.6 Direct evidence of the ConPET process in NMR investigation S71

6.7 Characterization of photocatalytic products S72

7. Cartesian coordinates of the optimized geometries by DFT calculations S75

8. References S106

1. Experimental section

1.1 Materials and methods

All manipulations were performed under an argon atmosphere using standard Schlenk techniques and a glovebox. THF, dioxane, Et_2O and CH_2Cl_2 were dried over Na/benzophenone or CaH_2, distilled prior to use. ^1H, $^{13}C\{^1H\}$ spectra were recorded on Bruker AVANCE III 400. Chemical shifts (δ) are expressed in ppm downfield from tetramethylsilane using the residual protonated solvent as an internal standard. Mass spectra were obtained with a Bruker micro TOF-Q II mass spectrometer (Bruker Daltonics Crop., USA) in the electrospray ionization (ESI) mode. UV-visible spectra were recorded using an Agilent Technologies Evolution 300 spectrophotometer. Prepared IPr(BIAN), 1,2-dihyroacenaphthylene-5-carbonitrile, **II**, [**1b**$^{2+}$][I^-]$_2$ and [**1c**$^{2+}$][I^-]$_2$ according to the literature procedure [1–6].

1.2 Synthesis and characterization of [1a^{2+}][I^-]$_2$, [1a^{2+}][BF_4^-]$_2$, [1a^{2+}][PF_6^-]$_2$, [1a$^{\bullet+}$][I^-] and 1a

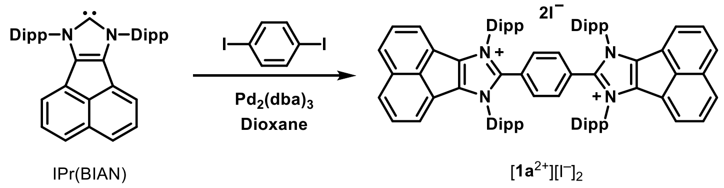

[**1a**$^{2+}$][I^-]$_2$: IPr(BIAN) (565 mg, 1.1 mmol), $Pd_2(dba)_3$ (14 mg, 0.015 mmol) and 1,4-diiodobenzene (165 mg, 0.5 mmol) were dissolved in 60 mL of dioxane and stirred at 120 °C for 30 h. And a lot of orange precipitate was formed. After cooling the reaction mixture to room temperature and this precipitate was filtered, washed with Et_2O. And dried under vacuum to yield [**1a**$^{2+}$][I^-]$_2$ as a yellow solid. Single crystals were grown by the diffusion of Et_2O into its concentrated MeCN solution. Yield: 339 mg, 50%. ^1H NMR (400 MHz, CD_3CN) δ = 8.17 (d, J = 8.2 Hz, 4H), 7.73-7.64 (m, 8H), 7.46 (d, J = 7.9 Hz, 8H), 7.38 (d, J = 6.9 Hz, 4H), 7.12 (s, 4H), 2.58 (sept, J = 6.7 Hz, 8H), 1.03 (d, J = 6.7 Hz, 24H), 0.84 (d, J = 6.8 Hz, 24H) ppm. $^{13}C\{^1H\}$ NMR (100 MHz, CD_3CN): δ = 145.8, 145.2, 139.5, 133.9, 132.3, 131.6, 131.2, 130.9, 129.6, 128.9, 127.0, 125.5, 123.5, 30.0, 25.5, 23.1 ppm. HRMS (ESI, positive ions): m/z = 550.3373 (calcd for $[C_{80}H_{84}N_4]^{2+}$ 550.3343) and m/z = 1227.5743 (calcd for $[C_{80}H_{84}N_4I]^+$ 1227.5735). UV-vis (THF): λ = 239, 324, 343 nm.

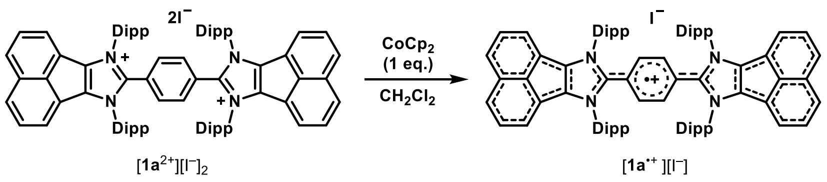

$[\mathbf{1a}^{\bullet+}][\mathrm{I}^-]$: $CoCp_2$ (19 mg, 0.1 mmol) was added to a CH_2Cl_2 (3 mL) solution of precursor salt $[\mathbf{1a}^{2+}][\mathrm{I}^-]_2$ (136 mg, 0.1 mmol) at room temperature. The reaction mixture was stirred for overnight. After which removal of the solvent under pressure. The residual solid was extracted with THF then slow diffusion of Et_2O into the concentrated solution led to the precipitation. The solution was decanted and the solvent removed to afford $[\mathbf{1a}^{\bullet+}][\mathrm{I}^-]$ as a dark purple powder. Single crystals were grown by the diffusion of Et_2O into its concentrated CH_2Cl_2 solution. Yield: 60 mg, 48%. UV-vis (THF): λ = 227, 322, 555, 702, 762 nm.

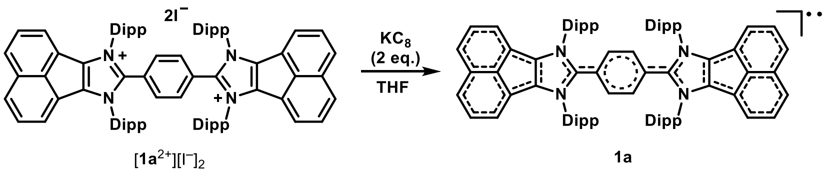

1a (Method A): KC_8 (27 mg, 0.2 mmol) was added to a THF (10 mL) solution of precursor salt $[\mathbf{1a}^{2+}][\mathrm{I}^-]_2$ (136 mg, 0.1 mmol) at −30 °C. The reaction mixture was brought to room temperature and further stirred for overnight. After which removal of the solvent under reduced pressure. The residual solid was extracted with Et_2O then filtered over Celite and removal of all volatiles afforded compound **1a** as a purple-red powder. Single crystals were grown by cooling a saturated toluene solution of **1a** at −30 °C. Yield: 98 mg, 89%. UV-vis (THF): λ = 215, 302, 462, 560 nm.

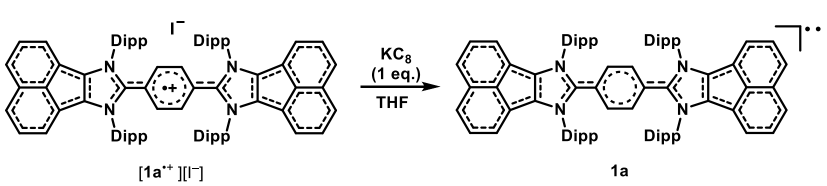

1a (Method B): KC_8 (14 mg, 0.1 mmol) was added to a THF (10 mL) solution of radical cation

[**1a**$^{\bullet+}$][I^-] (123 mg, 0.1 mmol) at −30 °C. The reaction mixture was brought to room temperature and further stirred for overnight. After which removal of the solvent under reduced pressure. The residual solid was extracted with Et_2O then filtered over Celite and removal of all volatiles afforded compound **1a**. Yield: 81 mg, 73%.

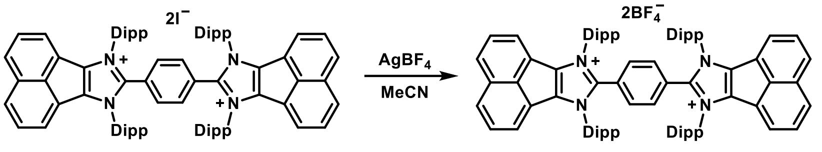

[$1a^{2+}$][I^-]$_2$ [$1a^{2+}$][BF_4^-]$_2$

[**1a**$^{2+}$][BF_4^-]$_2$: [**1a**$^{2+}$][I^-]$_2$ (136 mg, 0.1 mmol) was dissolved in MeCN, and $AgBF_4$ (39 mg, 0.2 mmol) was added. The mixture was stirred at room temperature overnight. Subsequently, the mixture was filtered, and the solvent was removed under vacuum. Et_2O was added to the residue, and the precipitate was collected by filtration. The solid was dried under vacuum to yield [**1a**$^{2+}$][BF_4^-]$_2$ as a yellow powder. Yield: 106 mg, 83%. ^{1}H NMR (400 MHz, CD_3CN) δ = 8.17 (d, J = 8.3 Hz, 4H), 7.72-7.64 (m, 8H), 7.46 (d, J = 7.9 Hz, 8H), 7.38 (d, J = 7.1 Hz, 4H), 7.13 (s, 4H), 2.59 (sept, J = 6.8 Hz, 8H), 1.03 (d, J = 6.7 Hz, 24H), 0.85 (d, J = 6.8 Hz, 24H) ppm. ^{13}C{^{1}H} NMR (100 MHz, CD_3CN): δ = 145.9, 145.2, 139.5, 133.9, 132.3, 131.6, 131.2, 130.9, 129.5, 129.0, 127.0, 125.5, 123.6, 30.0, 25.4, 23.1 ppm. ^{19}F NMR (376 MHz, CD_3CN): δ = −151.7 ppm. HRMS (ESI, positive ions): m/z = 550.3373 (calcd for [$C_{80}H_{84}N_4$]$^{2+}$ 550.3343). UV-vis (THF): λ = 235, 326, 344 nm.

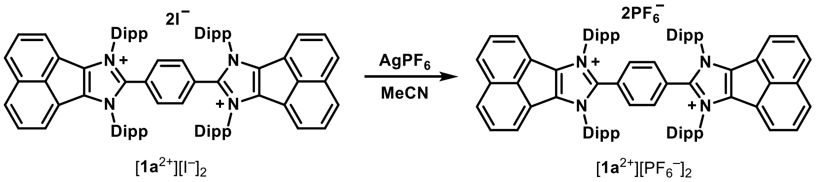

[$1a^{2+}$][I^-]$_2$ [$1a^{2+}$][PF_6^-]$_2$

[**1a**$^{2+}$][PF_6^-]$_2$: [**1a**$^{2+}$][I^-]$_2$ (136 mg, 0.1 mmol) was dissolved in MeCN, and $AgPF_6$ (51 mg, 0.2 mmol) was added. The mixture was stirred at room temperature overnight. Subsequently, the mixture was filtered, and the solvent was removed under vacuum. Et_2O was added to the residue, and the precipitate was collected by filtration. The solid was dried under vacuum to yield [**1a**$^{2+}$][PF_6^-]$_2$ as a yellow powder. Yield: 97 mg, 70%. ^{1}H NMR (400 MHz, CD_3CN) δ = 8.17 (d, J = 8.3 Hz, 4H), 7.73-

7.63 (m, 8H), 7.46 (d, J = 7.9 Hz, 8H), 7.38 (d, J = 7.1 Hz, 4H), 7.13 (s, 4H), 2.59 (sept, J = 6.8 Hz, 8H), 1.03 (d, J = 6.7 Hz, 24H), 0.85 (d, J = 6.8 Hz, 24H) ppm. $^{13}C\{^1H\}$ NMR (100 MHz, CD_3CN): δ = 145.9, 145.2, 139.5, 133.9, 132.3, 131.6, 131.2, 130.0, 129.6, 129.0, 127.0, 125.5, 123.6, 30.1, 25.5, 23.1 ppm. ^{19}F NMR (376 MHz, CD_3CN): δ = −71.9, −73.7 ppm. HRMS (ESI, positive ions): m/z = 550.3367 (calcd for $[C_{80}H_{84}N_4]^{2+}$ 550.3343). UV-vis (THF): λ = 236, 329, 345 nm.

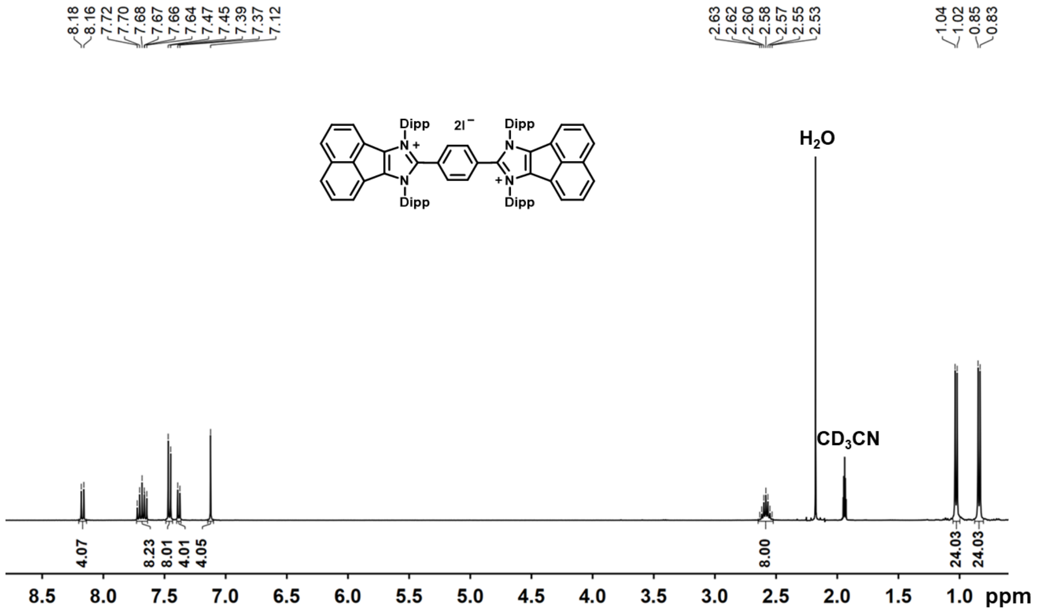

Figure S1. ^1H NMR spectrum of [$\mathbf{1a}^{2+}$][I^-]$_2$ (400 MHz, CD_3CN).

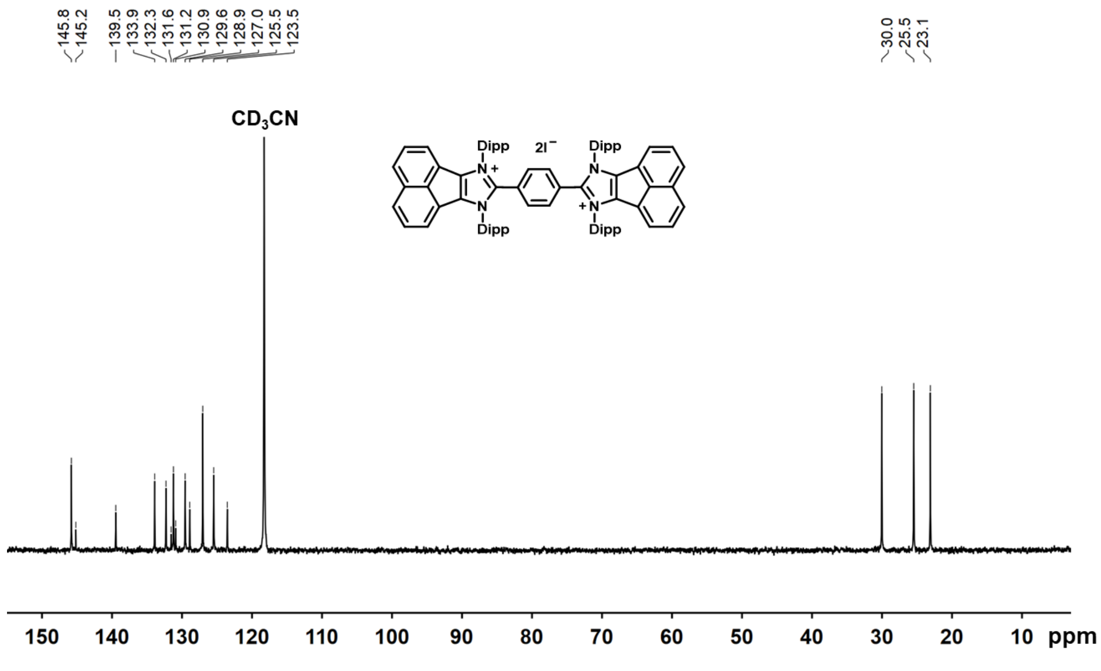

Figure S2. $^{13}C\{^1H\}$ NMR spectrum of [$\mathbf{1a}^{2+}$][I^-]$_2$ (100 MHz, CD_3CN).

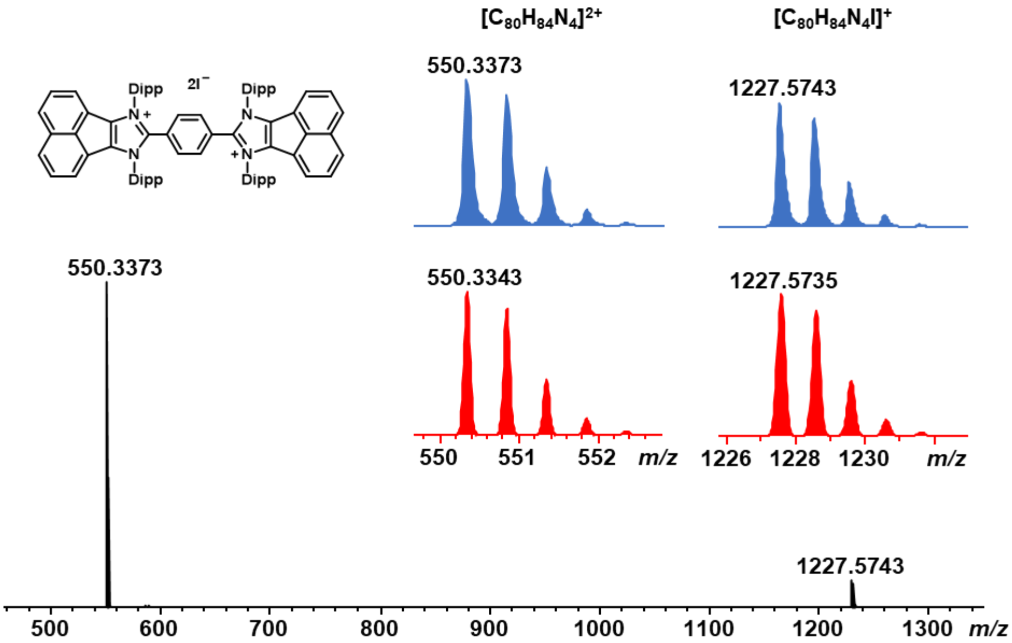

Figure S3. The HRMS spectrum of [**1a**$^{2+}$][I^{-}]$_2$, inset experimental (blue) and calculated (red) isotope distributions for peaks corresponding to $[C_{80}H_{84}N_4]^{2+}$ and $[C_{80}H_{84}N_4I]^{+}$.

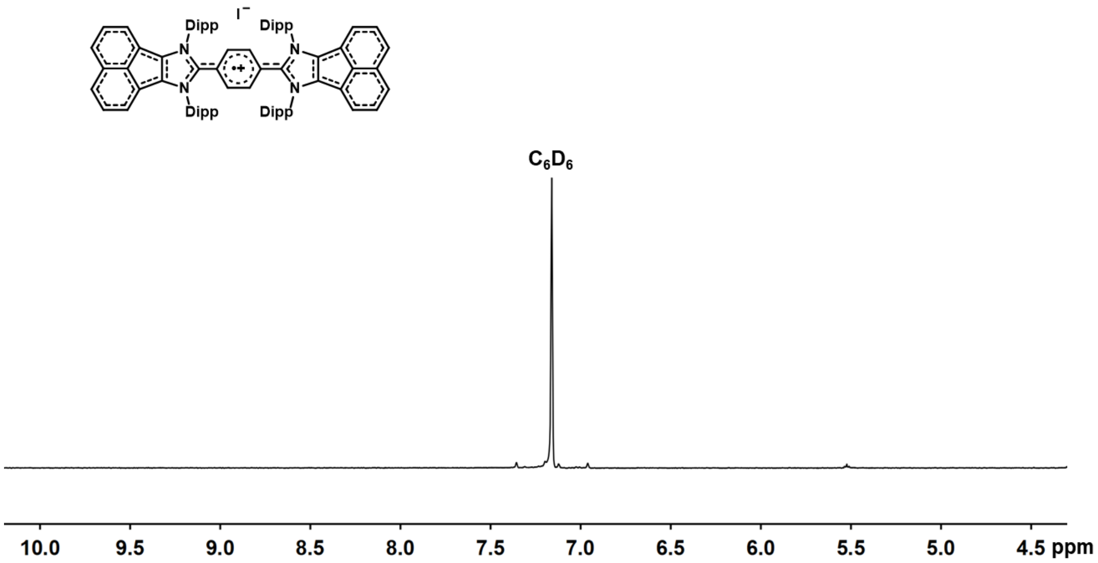

Figure S4. ^{1}H NMR spectrum of [**1a**$^{•+}$][I^{-}] (400 MHz, C_6D_6).

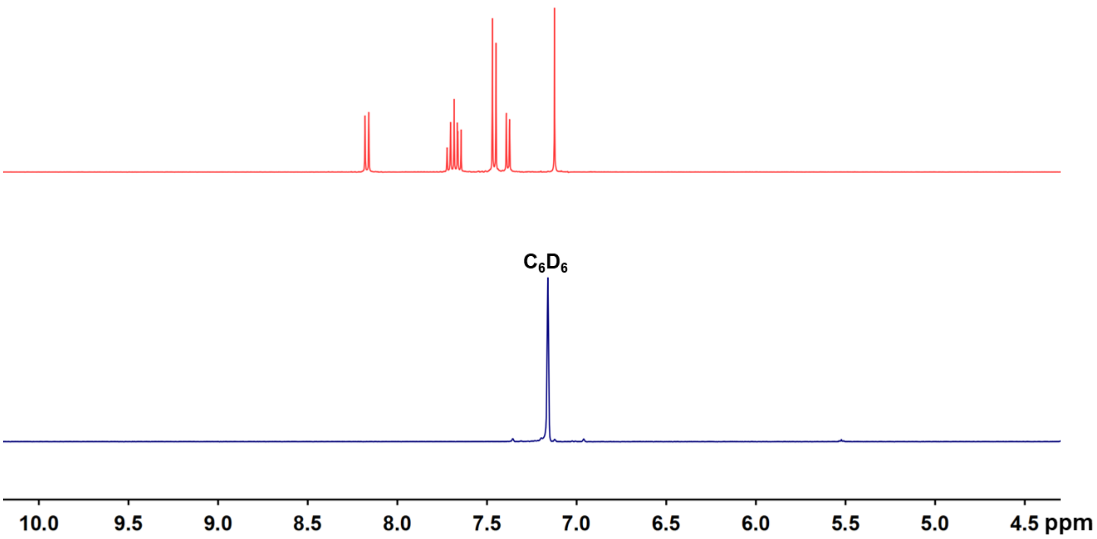

Figure S5. Comparison of ^{1}H NMR spectra of [**1a**$^{2+}$][I^{-}]$_2$ and [**1a**$^{\bullet+}$][I^{-}]. The proton signal of the single-electron reduction product [**1a**$^{\bullet+}$][I^{-}] disappears, demonstrating that it has paramagnetic properties.

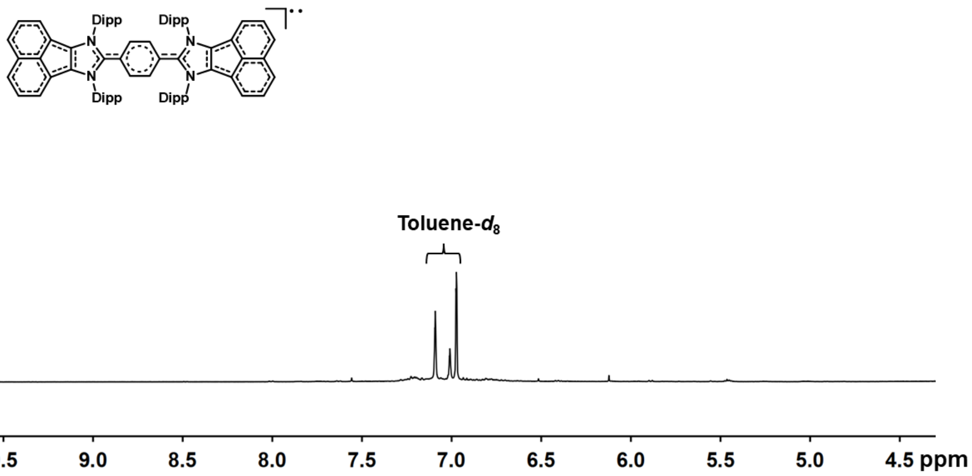

Figure S6. ^{1}H NMR spectrum of **1a** (400 MHz, toluene-d_8).

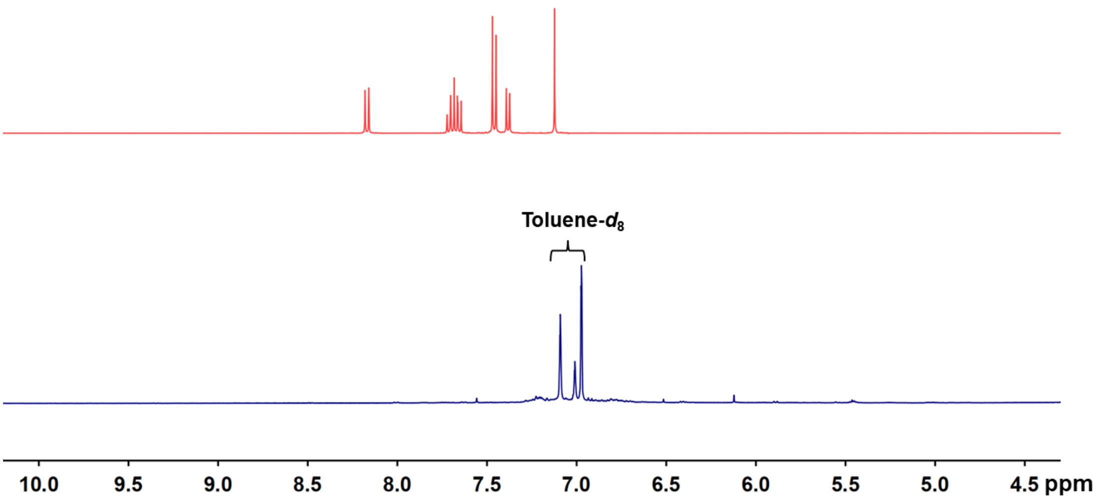

Figure S7. Comparison of ^{1}H NMR spectra of [**1a**$^{2+}$][I$^-$]$_2$ and **1a**. The proton signal of **1a** in the aromatic region disappears, indicating a substantial population of the triplet state at room temperature.

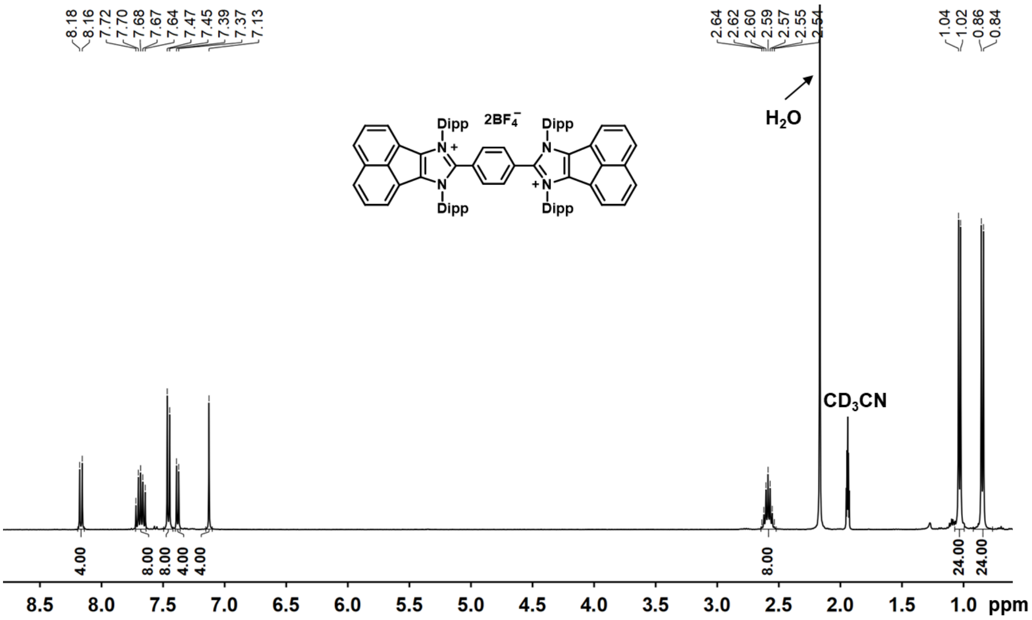

Figure S8. ^{1}H NMR spectrum of [**1a**$^{2+}$][BF$_4^-$]$_2$ (400 MHz, CD$_3$CN).

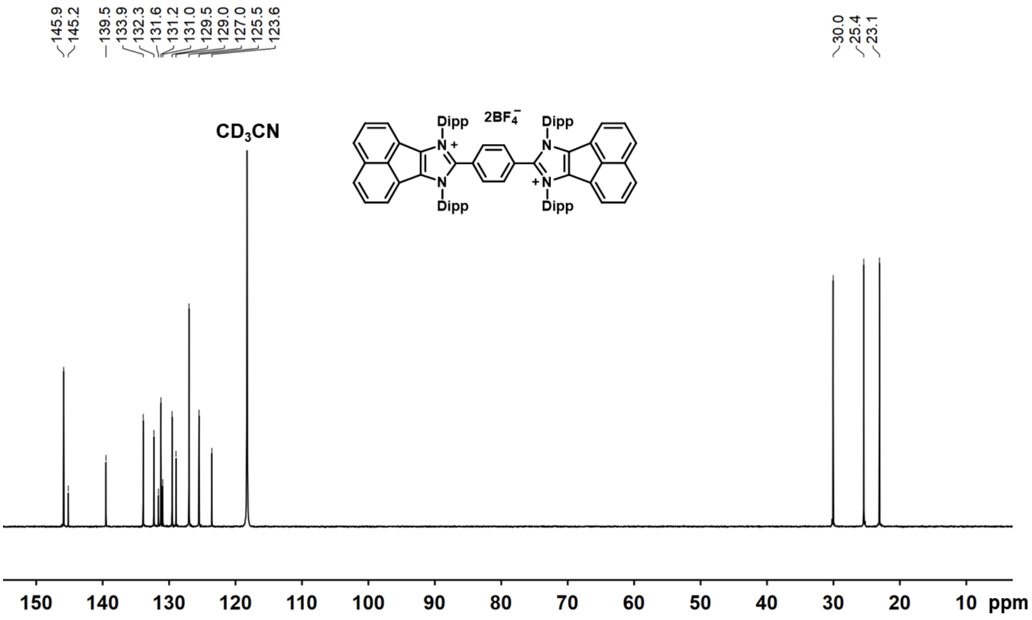

Figure S9. $^{13}C\{^{1}H\}$ NMR spectrum of [**1a**$^{2+}$][BF_4^-]$_2$ (100 MHz, CD_3CN).

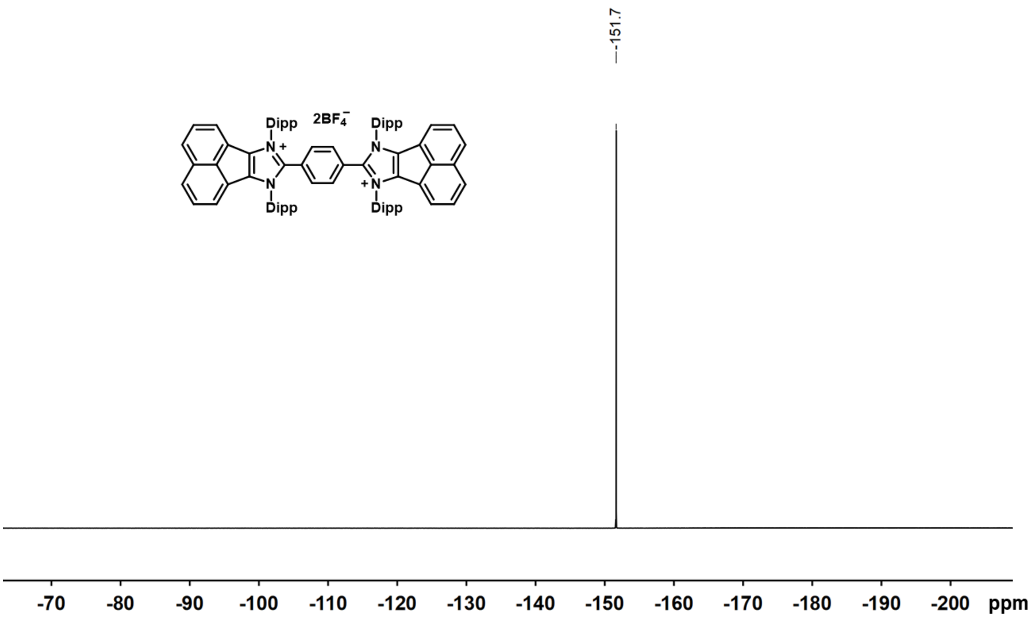

Figure S10. ^{19}F NMR spectrum of [**1a**$^{2+}$][BF_4^-]$_2$ (376 MHz, CD_3CN).

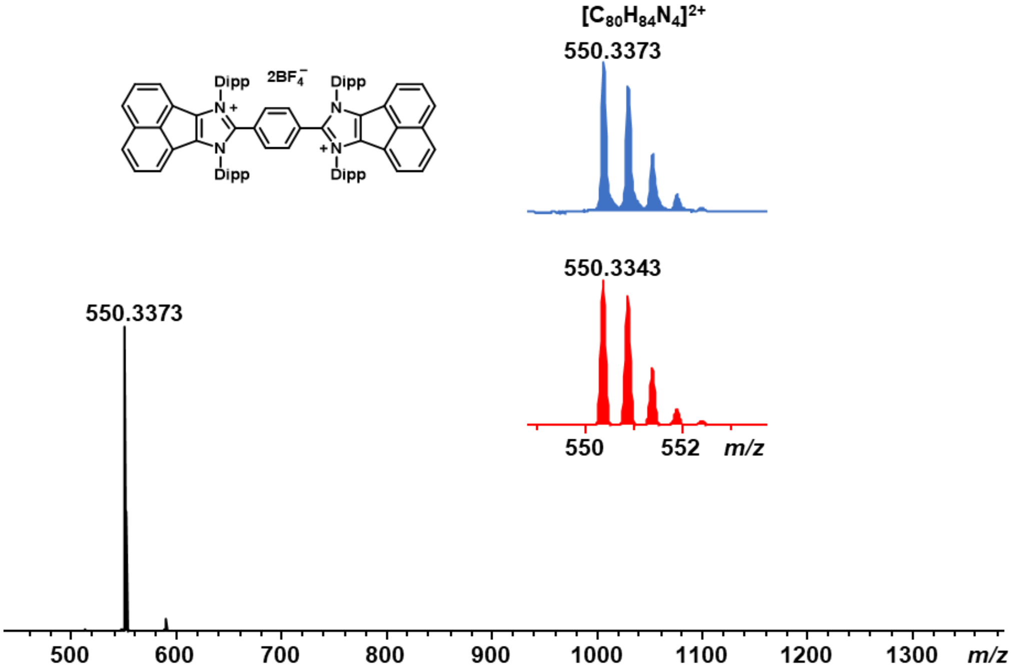

Figure S11. The HRMS spectrum of [**1a**$^{2+}$][BF_4^-]$_2$, inset experimental (blue) and calculated (red) isotope distributions for peaks corresponding to [$C_{80}H_{84}N_4$]$^{2+}$.

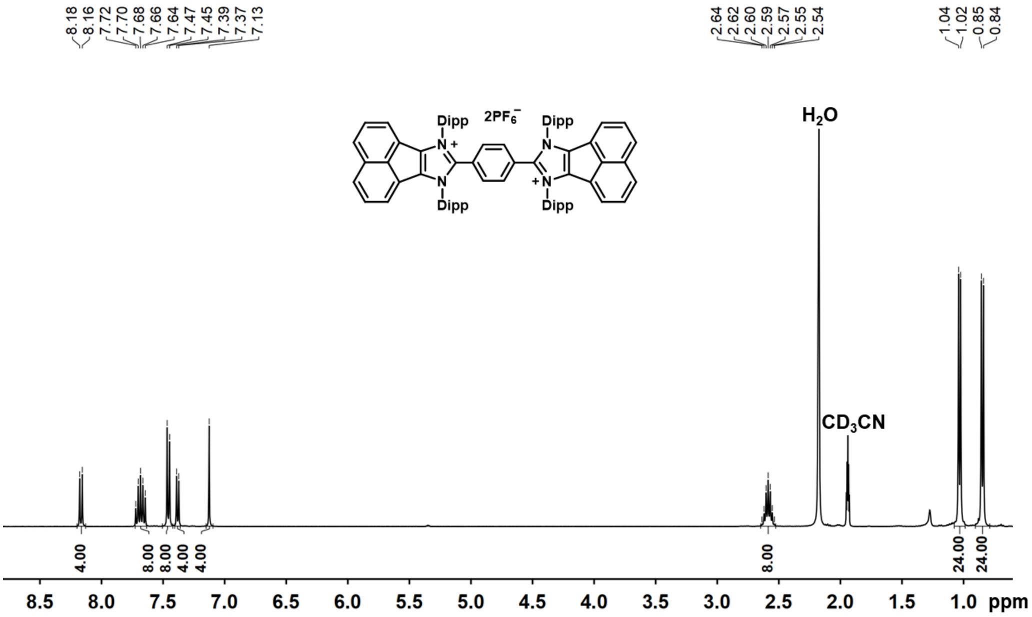

Figure S12. ^{1}H NMR spectrum of [**1a**$^{2+}$][PF_6^-]$_2$ (400 MHz, CD_3CN).

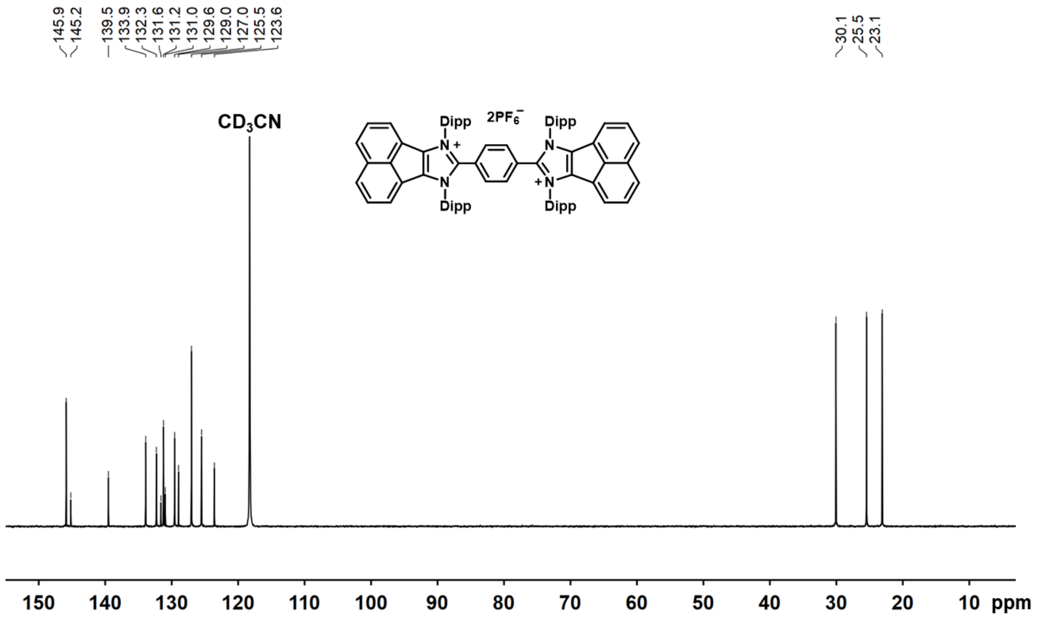

Figure S13. $^{13}C\{^{1}H\}$ NMR spectrum of [**1a**$^{2+}$][PF_6^{-}]$_2$ (100 MHz, CD_3CN).

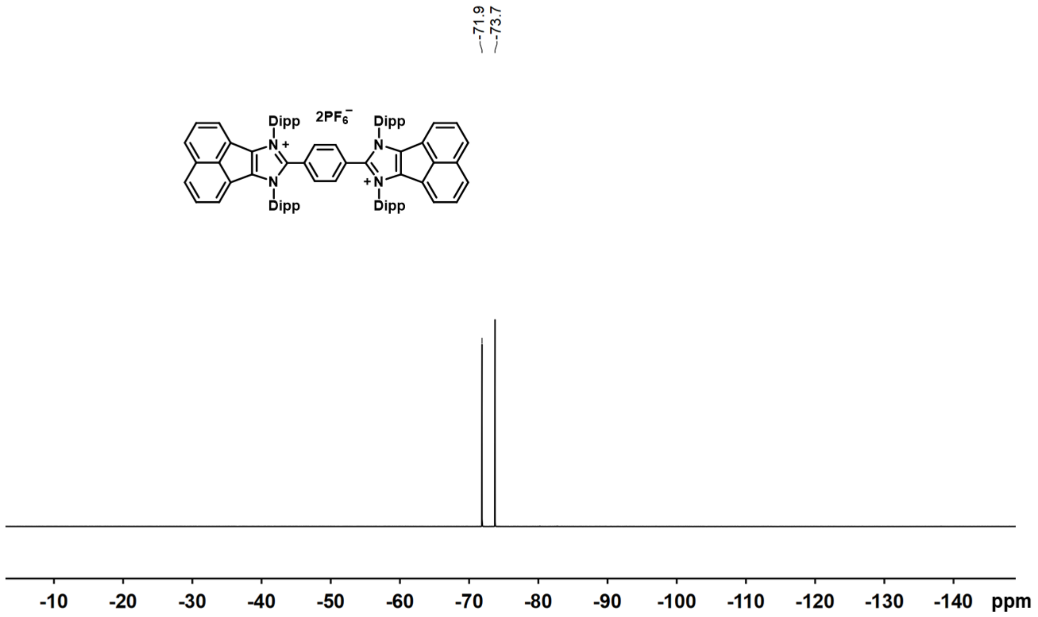

Figure S14. ^{19}F NMR spectrum of [**1a**$^{2+}$][PF_6^{-}]$_2$ (376 MHz, CD_3CN).

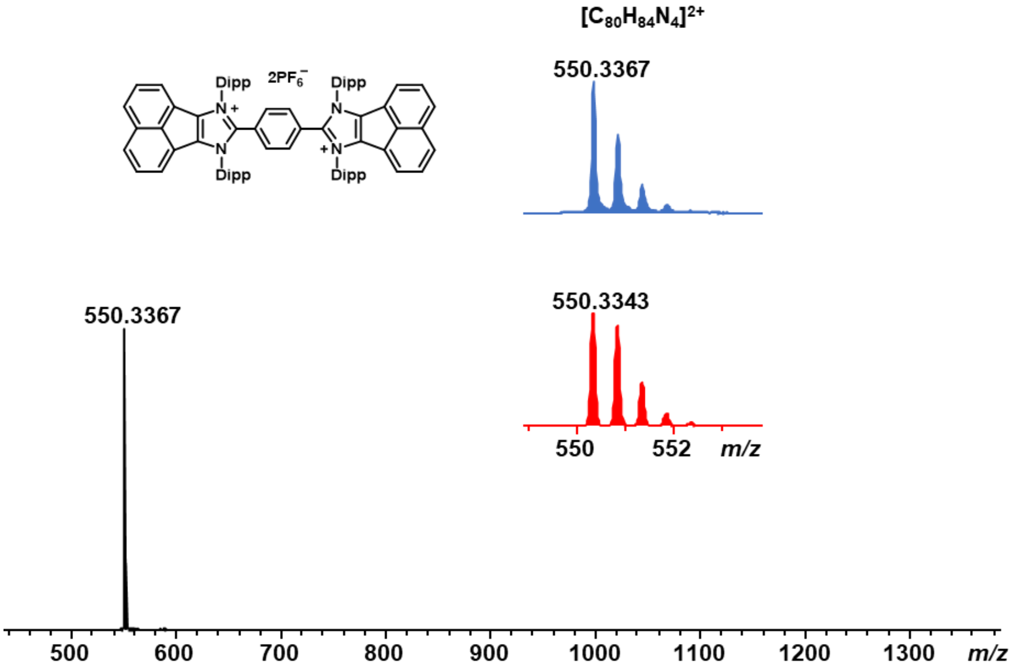

Figure S15. The HRMS spectrum of [**1a**$^{2+}$][PF_6^-]$_2$, inset experimental (blue) and calculated (red) isotope distributions for peaks corresponding to [$C_{80}H_{84}N_4$]$^{2+}$.

1.3 Synthesis and characterization of [**1d**$^{2+}$][I^-]$_2$, [**1e**$^{2+}$][I^-]$_2$, [**1f**$^{2+}$][I^-]$_2$ and [**1g**$^{2+}$][I^-]$_2$

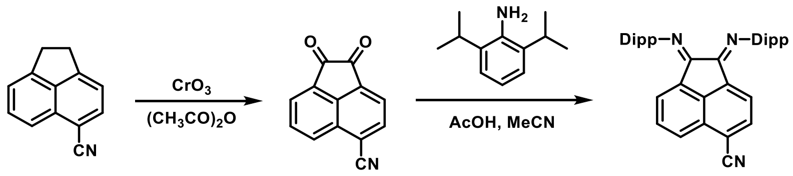

I

I: 1,2-dihyroacenaphthylene-5-carbonitrile (723 mg, 4.0 mmol) was dissolved in 25 mL of acetic anhydride at 110 °C. CrO_3 (1.6 g, 16.0 mmol) was added carefully to the stirred solution. The resulting green suspension was stirred at 110 °C for 1 h. After cooling the reaction mixture to room temperature and poured into ice water. Concentrated H_2SO_4 (5 mL) was added and the mixture was filtered. The yellow precipitate was washed with water and dried in vacuum. Subsequently, this precipitate in 30 mL of MeCN was refluxed at 90 °C for 30 min. Then 3 mL of acetic acid was added and heating was continued until the solid had completely dissolved. To this hot solution, 2,6-diisopropylaniline (0.9 mL, 4.8 mmol) was added directly and the solution was heated at 120 °C for

12 h. It was then cooled to room temperature and the precipitate was filtered, washed with hexane. And dried under vacuum to yield **I** as a red solid. Yield: 611 mg, 74%. ^{1}H NMR (400 MHz, $CDCl_3$) δ = 8.18 (d, J = 8.3 Hz, 1H), 7.76 (d, J = 7.5 Hz, 1H), 7.59 (t, J = 7.9 Hz, 1H), 7.29 (s, 6H), 6.76 (d, J = 7.1 Hz, 1H), 6.63 (d, J = 7.3 Hz, 1H), 2.96 (m, 4H), 1.23 (d, J = 6.8 Hz, 12H), 0.96 (d, J = 6.8 Hz, 12H) ppm. ^{13}C{^{1}H} NMR (100 MHz, $CDCl_3$): δ = 159.6, 159.4, 146.9, 139.5, 135.0, 134.8, 134.1, 132.7, 130.6, 130.4, 129.9, 126.6, 125.0, 124.7, 123.6, 122.0, 116.2, 110.9, 28.6, 23.2, 23.0 ppm.

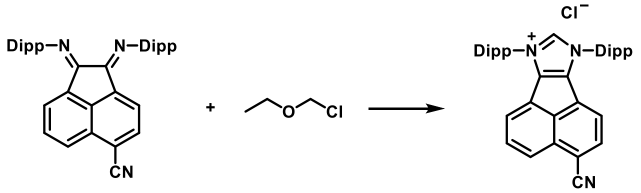

IPr(BIAN)^{CN}·HCl: **I** (526 mg, 1.0 mmol) and chloromethyl ethyl ether (3.5 mL, 37.5 mmol) were stirred at 100 °C for 16 h. The reaction mixture was then cooled down to room temperature and diluted with hexane and filtered. The product was obtained as a yellow solid after the filtration. Yield: 223 mg, 39%. ^{1}H NMR (400 MHz, $CDCl_3$): δ = 12.55 (s, 1H), 8.22 (d, J = 8.4 Hz, 1H), 7.95 (d, J = 7.2 Hz, 1H), 7.69 (q, J = 7.5 Hz, 3H), 7.47 (d, J = 7.7 Hz, 4H), 7.22 (d, J = 7.3 Hz, 2H), 2.68 (s, 4H), 1.41 (d, J = 6.3 Hz, 12H), 1.16 (dd, J = 6.8, 3.1 Hz, 12H) ppm. ^{13}C{^{1}H} NMR (100 MHz, $CDCl_3$): δ = 145.3, 144.7, 139.3, 136.4, 134.7, 132.6, 132.5, 130.8, 130.3, 129.4, 128.7, 128.6, 128.0, 127.2, 125.1, 124.6, 123.7, 121.5, 115.8, 112.4, 29.4, 24.7, 24.6, 23.5, 23.4 ppm. HRMS (ESI, positive ions): m/z = 538.3205 (calcd for $[C_{38}H_{40}N_3]^+$ 538.3217).

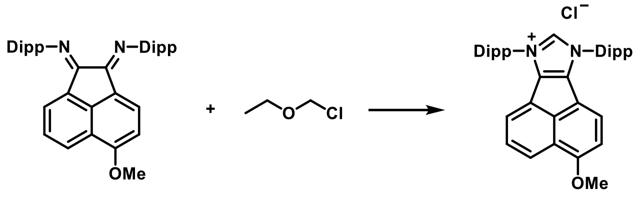

IPr(BIAN)^{OMe}·HCl: II (531 mg, 1.0 mmol) and chloromethyl ethyl ether (3.5 mL, 37.5 mmol)

were stirred at 100 °C for 16 h. The reaction mixture was then cooled down to room temperature and diluted with hexane and filtered. The product was obtained as a yellow solid after the filtration. Single crystals were grown by the diffusion of pentane into its concentrated chloroform solution. Yield: 475 mg, 82%. ^{1}H NMR (400 MHz, CD_3CN): δ = 9.67-9.60 (m, 1H), 8.27 (d, J = 8.4 Hz, 1H), 7.77 (t, J = 7.8 Hz, 2H), 7.59 (d, J = 7.9 Hz, 5H), 7.34 (d, J = 7.1 Hz, 1H), 7.29 (d, J = 7.9 Hz, 1H), 6.92 (d, J = 8.0 Hz, 1H), 4.03 (s, 3H), 2.73 (pd, J = 6.9, 2.0 Hz, 4H), 1.24 (d, J = 6.8 Hz, 12H), 1.16 (t, J = 6.4 Hz, 12H) ppm. $^{13}C\{^1H\}$ NMR (100 MHz, CD_3CN): δ = 161.2, 146.4, 140.1, 139.0, 133.3, 133.1, 129.9, 128.6, 126.8, 126.1, 125.9, 124.1, 123.4, 107.6, 57.3, 29.9, 24.8, 24.7, 23.6, 23.5 ppm. HRMS (ESI, positive ions): m/z = 543.3319 (calcd for $[C_{38}H_{43}N_2O]^+$ 543.3370).

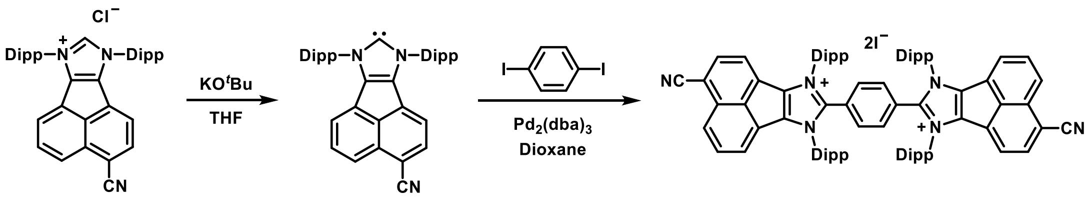

IPr(BIAN)^{CN}·HCl IPr(BIAN)^{CN} [**1d**$^{2+}$][I$^-$]$_2$

[**1d**$^{2+}$][I$^-$]$_2$: IPr(BIAN)^{CN}·HCl (172 mg, 0.3 mmol) and KO^{t}Bu (50 mg, 0.45 mmol) were dissolved in 15 mL of THF and stirred at room temperature for 5 h. Following this time, reaction mixture was filtered and the solvent was removed under reduced pressure, the red solid was obtained. Then $Pd_2(dba)_3$ (4 mg, 0.004 mmol), 1,4-diiodobenzene (45 mg, 0.14 mmol) and dioxane (15 mL) were added and stirred at 120 °C for 24 h. After cooling the reaction mixture to room temperature and this precipitate was filtered, washed with Et_2O. And dried under vacuum to yield [**1d**$^{2+}$][I$^-$]$_2$ as an orange solid. Yield: 51 mg, 26%. ^{1}H NMR (400 MHz, CD_3CN): δ = 8.26 (d, J = 8.4 Hz, 1H), 8.10 (d, J = 7.4 Hz, 1H), 7.78 (dd, J = 8.4, 7.2 Hz, 1H), 7.71 (t, J = 7.9 Hz, 2H), 7.50-7.37 (m, 6H), 7.13 (s, 2H), 2.63-2.50 (m, 4H), 1.03 (dd, J = 6.6, 4.2 Hz, 12H), 0.83 (dd, J = 6.8, 1.6 Hz, 12H) ppm. $^{13}C\{^1H\}$ NMR (100 MHz, CD_3CN): δ = 146.4, 145.8, 141.4, 138.7, 136.3, 134.1, 131.9, 131.5, 131.4, 130.0, 129.3, 128.6, 128.5, 127.9, 127.2, 127.0, 125.4, 124.5, 124.3, 116.9, 113.4, 30.0, 25.5, 23.1 ppm. HRMS (ESI, positive ions): m/z = 575.3279 (calcd for $[C_{82}H_{82}N_6]^{2+}$ 575.3295) and m/z = 1277.5666 (calcd for $[C_{82}H_{82}N_6I]^+$ 1277.5640). UV-vis (THF): λ = 236, 336 nm.

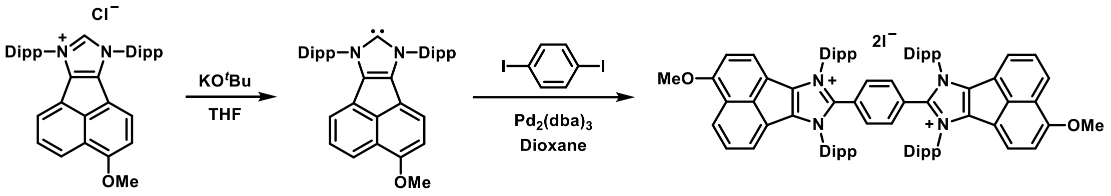

IPr(BIAN)^{OMe}·HCl IPr(BIAN)^{OMe} [$1e^{2+}$][I^-]$_2$

[$\mathbf{1e}^{2+}$][I^-]$_2$: IPr(BIAN)^{OMe}·HCl (255 mg, 0.44 mmol) and KO^{t}Bu (34 mg, 0.3 mmol) were dissolved in 20 mL of THF and stirred at room temperature for 5 h. Following this time, reaction mixture was filtered and the solvent was removed under reduced pressure, the yellow solid was obtained. Then $Pd_2(dba)_3$ (5 mg, 0.006 mmol), 1,4-diiodobenzene (66 mg, 0.2 mmol) and dioxane (15 mL) were added and stirred at 120 °C for 24 h. After cooling the reaction mixture to room temperature and this precipitate was filtered, washed with Et_2O. And dried under vacuum to yield [$\mathbf{1e}^{2+}$][I^-]$_2$ as a yellow solid. Single crystals were grown by the diffusion of Et_2O into its concentrated MeCN solution. Yield: 113 mg, 40%. ^{1}H NMR (400 MHz, CD_3CN): δ = 8.30 (d, J = 8.0 Hz, 1H), 7.69 (t, J = 7.9 Hz, 2H), 7.61 (dd, J = 8.4, 7.2 Hz, 1H), 7.45 (d, J = 7.9 Hz, 4H), 7.34 (d, J = 7.0 Hz, 1H), 7.30 (d, J = 8.0 Hz, 1H), 7.09 (s, 2H), 6.94 (d, J = 8.1 Hz, 1H), 4.04 (s, 3H), 2.57 (pd, J = 6.7, 2.0 Hz, 4H), 1.03 (dd, J = 10.2, 6.7 Hz, 12H), 0.84 (dd, J = 6.8, 1.9 Hz, 12H) ppm. ^{13}C{^{1}H} NMR (100 MHz, CD_3CN): δ = 161.6, 145.9, 145.8, 144.2, 139.4, 137.7, 133.8, 133.1, 131.1, 129.1, 129.0, 128.6, 128.0, 126.9, 126.5, 125.5, 125.1, 124.0, 123.0, 115.2, 107.8, 57.5, 30.1, 30.0, 25.5, 25.4, 23.1, 23.0 ppm. HRMS (ESI, positive ions): m/z = 580.3450 (calcd for $[C_{82}H_{88}N_4O_2]^{2+}$ 580.3448). UV-vis (THF): λ = 239, 363 nm.

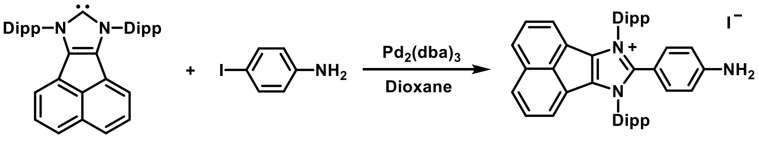

IPr(BIAN) [III^+][I^-]

[$\mathbf{III}^+$][I^-]: IPr(BIAN) (550 mg, 1.0 mmol), $Pd_2(dba)_3$ (8 mg, 0.03 mmol) and 4-iodoaniline (219 mg, 1.0 mmol) were dissolved in 60 mL of dioxane and stirred at 120 °C for 16 h. And a lot of yellow precipitate was formed. After cooling the reaction mixture to room temperature and this precipitate was filtered, washed with ethyl acetate. And dried under vacuum to yield [$\mathbf{III}^+$][I^-] as a yellow solid.

Yield: 241 mg, 33%. ^{1}H NMR (400 MHz, CD_3CN): δ = 8.08 (d, J = 8.3 Hz, 2H), 7.78 (t, J = 7.9 Hz, 2H), 7.68-7.52 (m, 6H), 7.22 (d, J = 7.0 Hz, 2H), 6.89 (d, J = 9.0 Hz, 2H), 6.44 (d, J = 9.0 Hz, 2H), 4.96 (s, 2H), 2.78 (sept, J = 6.7 Hz, 4H), 1.09 (dd, J = 6.8, 2.5 Hz, 24H) ppm. $^{13}C\{^1H\}$ NMR (100 MHz, CD_3CN): δ = 153.4, 149.3, 146.1, 137.9, 133.4, 131.5, 131.4, 130.9, 130.8, 129.3, 127.0, 124.5, 114.4, 108.1, 30.1, 25.0, 23.2 ppm. HRMS (ESI, positive ions): m/z = 604.3641 (calcd for $[C_{43}H_{44}N_3]^+$ 604.3686).

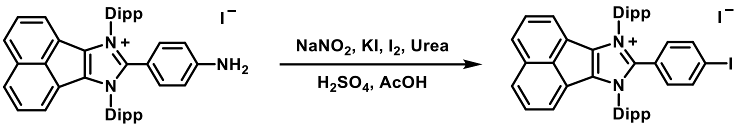

[III$^+$][I$^-$]

[IV$^+$][I$^-$]

[**IV**$^+$][I$^-$]: A flask containing $NaNO_2$ (97 mg, 1.4 mmol) was cooled to 0 °C. Under stirring, H_2SO_4 (0.6 mL) was added slowly. Acetic acid (1 mL) was then added, and the mixture was stirred for 10 min. In the meantime, [**III**$^+$][I$^-$] (732 mg, 1.0 mmol) was dissolved in acetic acid. Under stirring at 0 °C, H_2SO_4 (0.1 mL) was added slowly. The resulting white precipitate was dissolved by addition of a minimum of water. This solution was added dropwise very slowly to the $NaNO_2$/H_2SO_4 solution at 0 °C. After 30 min of continued stirring, the reaction mixture was added dropwise but rapidly under vigorous stirring to a solution of KI (345 mg, 1.36 mmol), I_2 (387 mg, 2.33 mmol) and urea (60 mg, 1.0 mmol) in 3 mL water and 2 mL of chloroform at 0 °C. The reaction mixture was stirred overnight while warming to room temperature. The two layers were separated in a separation funnel, and the aqueous layer was extracted with CH_2Cl_2. The organic phases were combined, dried over Na_2SO_4, and evaporated under reduced pressure. And dried under vacuum to yield [**IV**$^+$][I$^-$] as a yellow solid. Single crystals were grown by the diffusion of hexane into its concentrated THF solution. Yield: 674 mg, 80%. ^{1}H NMR (400 MHz, CD_3CN): δ = 8.15 (d, J = 8.3 Hz, 2H), 7.86-7.71 (m, 4H), 7.66 (dd, J = 8.3, 7.2 Hz, 2H), 7.58 (d, J = 7.9 Hz, 4H), 7.33 (d, J = 6.9 Hz, 2H), 6.94 (d, J = 8.8 Hz, 2H), 2.74 (sept, J = 6.7 Hz, 4H), 1.10 (dd, J = 6.8, 4.8 Hz, 24H) ppm. $^{13}C\{^1H\}$ NMR (100 MHz, CD_3CN): δ = 147.0, 146.0, 139.7, 139.1, 133.8, 132.0, 131.3, 131.2, 130.9, 129.7, 129.5, 127.1, 125.2, 123.8, 121.0, 101.9, 30.2, 25.3, 23.2 ppm. HRMS (ESI, positive ions): m/z = 715.2512 (calcd for $[C_{43}H_{44}N_3]^+$ 715.2544).

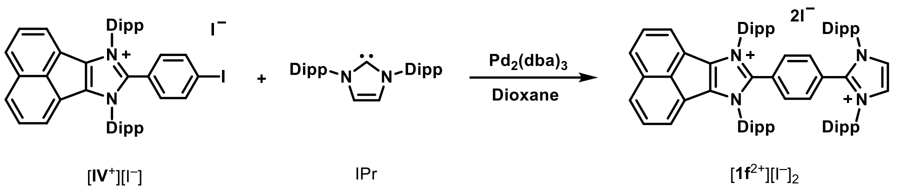

[$\mathbf{1f}^{2+}$][I^-]$_2$: [$\mathbf{IV}^+$][I^-] (169 mg, 0.2 mmol), $Pd_2(dba)_3$ (5 mg, 0.006 mmol) and IPr (170 mg, 0.4 mmol) were dissolved in 15 mL of dioxane and stirred at 120 °C for 24 h. After cooling the reaction mixture to room temperature and the solvent was removed under reduced pressure. The residue was washed with Et_2O. The crude mixture was chromatographed on neutral alumina (CH_2Cl_2/methanol, starting at 40:1 and gradually increasing the proportion of methanol) to give [$\mathbf{1f}^{2+}$][I^-]$_2$ as yellow solid. Yield: 54 mg, 23%. ^{1}H NMR (400 MHz, CD_3CN): δ = 8.16 (d, J = 8.3 Hz, 2H), 8.07 (s, 2H), 7.66 (q, J = 8.4 Hz, 4H), 7.54 (t, J = 7.9 Hz, 2H), 7.43 (d, J = 7.8 Hz, 4H), 7.36 (d, J = 7.1 Hz, 2H), 7.30 (d, J = 7.9 Hz, 4H), 7.09 (d, J = 8.4 Hz, 2H), 6.91 (d, J = 8.4 Hz, 2H), 2.55 (dp, J = 12.6, 6.2 Hz, 4H), 2.34-2.25 (m, 4H), 1.17 (d, J = 6.7 Hz, 12H), 1.01 (d, J = 6.6 Hz, 12H), 0.82 (dd, J = 11.8, 6.8 Hz, 24H) ppm. ^{13}C{^{1}H} NMR (100 MHz, CD_3CN): δ = 145.8, 145.6, 145.3, 144.3, 139.4, 133.8, 133.4, 132.3, 131.5, 131.3, 131.2, 130.9, 129.8, 129.5, 128.8, 127.7, 126.9, 126.2, 125.5, 125.4, 125.3, 123.5, 30.0, 29.9, 25.6, 25.5, 23.1, 22.5 ppm. HRMS (ESI, positive ions): m/z = 488.3165 (calcd for $[C_{70}H_{80}N_4]^{2+}$ 488.3186). UV-vis (THF): λ = 236, 323 nm.

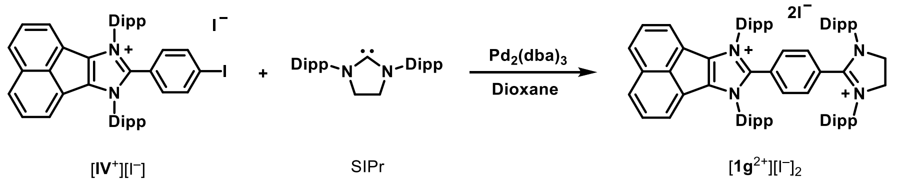

[$\mathbf{1g}^{2+}$][I^-]$_2$: [$\mathbf{IV}^+$][I^-] (169 mg, 0.2 mmol), $Pd_2(dba)_3$ (5 mg, 0.006 mmol) and SIPr (171 mg, 0.4 mmol) were dissolved in 15 mL of dioxane and stirred at 120 °C for 24 h. After cooling the reaction mixture to room temperature and the solvent was removed under reduced pressure. The residue was washed with Et_2O. The crude mixture was chromatographed on neutral alumina (CH_2Cl_2/methanol, starting at 40:1 and gradually increasing the proportion of methanol) to give [$\mathbf{1g}^{2+}$][I^-]$_2$ as yellow solid. Yield: 71 mg, 30%. ^{1}H NMR (400 MHz, CD_3CN): δ = 8.18 (d, J = 8.3 Hz, 2H), 7.69 (q, J = 8.2 Hz, 4H), 7.49-7.41 (m, 6H), 7.39 (d, J = 7.1 Hz, 2H), 7.22 (d, J = 7.8 Hz, 4H), 7.09 (d, J = 8.6

Hz, 2H), 6.96 (d, J = 8.6 Hz, 2H), 4.53 (s, 4H), 2.93 (sept, J = 6.4 Hz, 4H), 2.54 (sept, J = 6.7 Hz, 4H), 1.29 (d, J = 6.7 Hz, 12H), 1.03 (d, J = 6.7 Hz, 12H), 0.85 (d, J = 6.8 Hz, 12H), 0.79 (d, J = 6.8 Hz, 12H) ppm. $^{13}C\{^1H\}$ NMR (100 MHz, CD_3CN): δ = 166.1, 146.6, 145.8, 145.2, 139.4, 133.9, 132.5, 132.3, 131.5, 131.1, 130.9, 130.7, 130.5, 129.5, 128.7, 126.9, 126.6, 126.1, 125.5, 123.5, 54.7, 30.0, 29.6, 26.4, 25.6, 23.2, 23.1 ppm. HRMS (ESI, positive ions): m/z = 489.3210 (calcd for $[C_{70}H_{82}N_4]^{2+}$ 489.3264). UV-vis (THF): λ = 236, 324 nm.

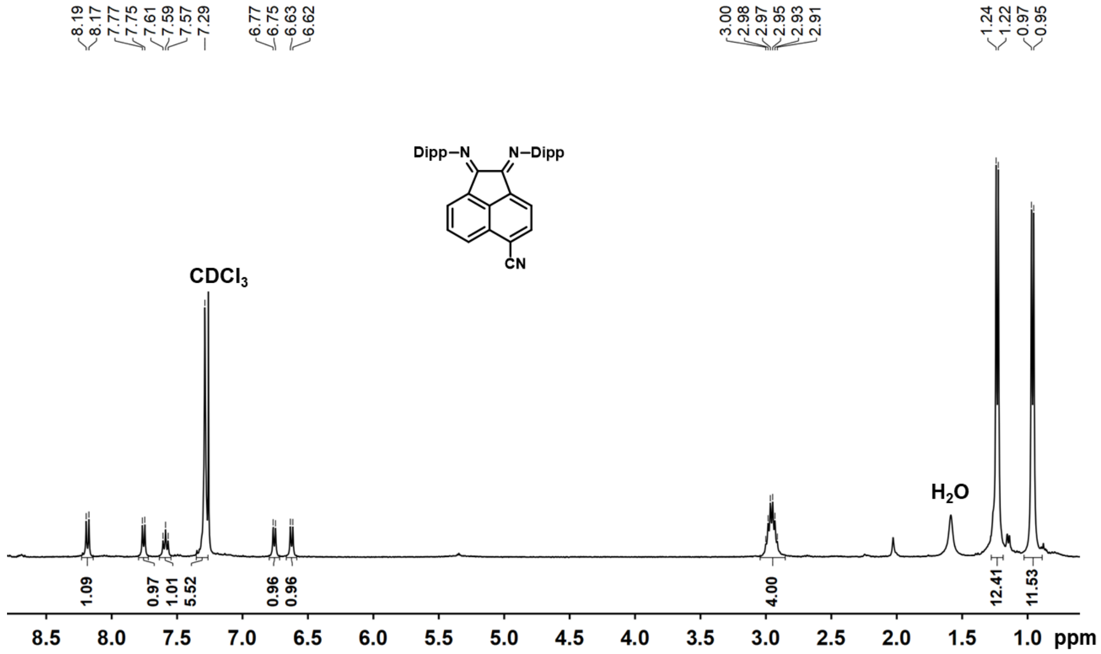

Figure S16. ^1H NMR spectrum of **I** (400 MHz, $CDCl_3$).

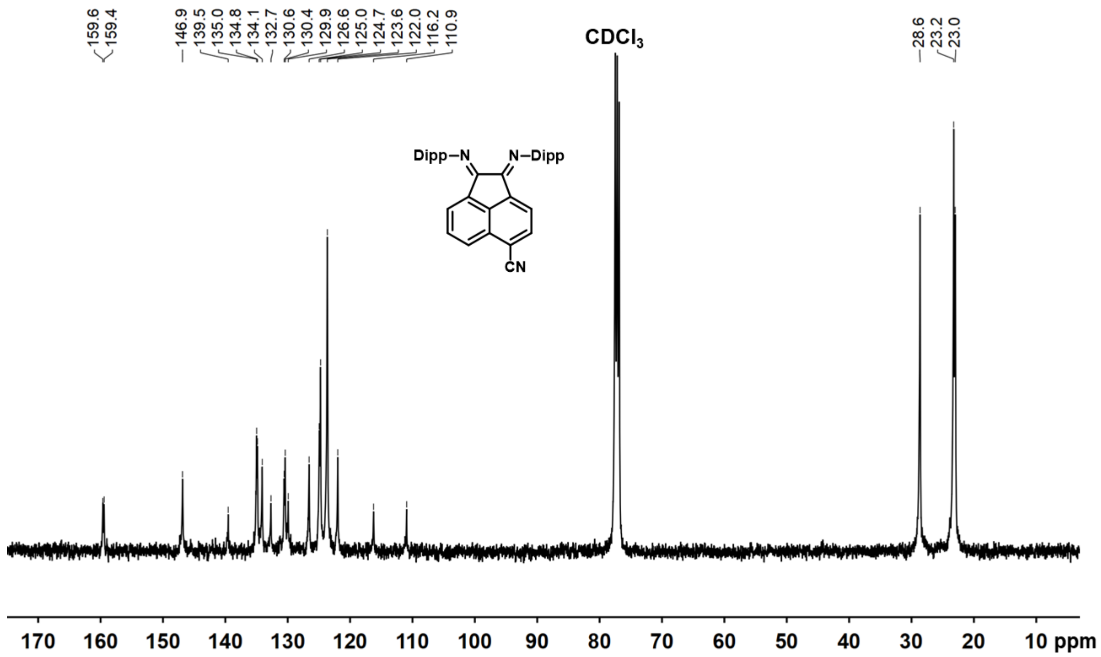

Figure S17. $^{13}C\{^{1}H\}$ NMR spectrum of **I** (100 MHz, $CDCl_3$).

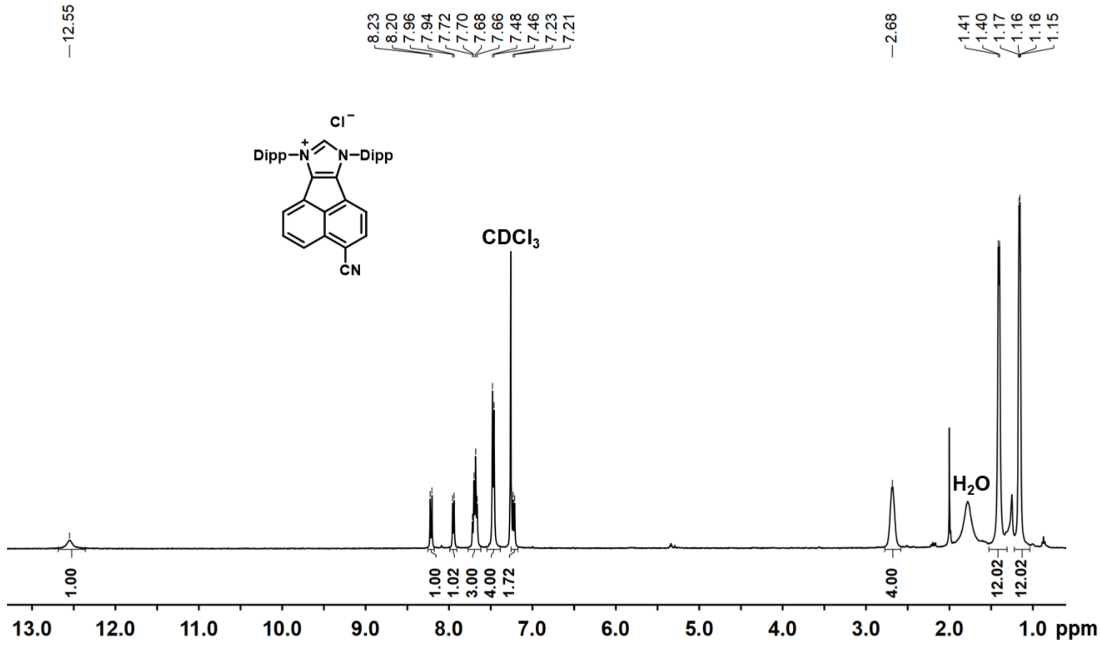

Figure S18. ^{1}H NMR spectrum of IPr(BIAN)$^{CN}\cdot$HCl (400 MHz, $CDCl_3$).

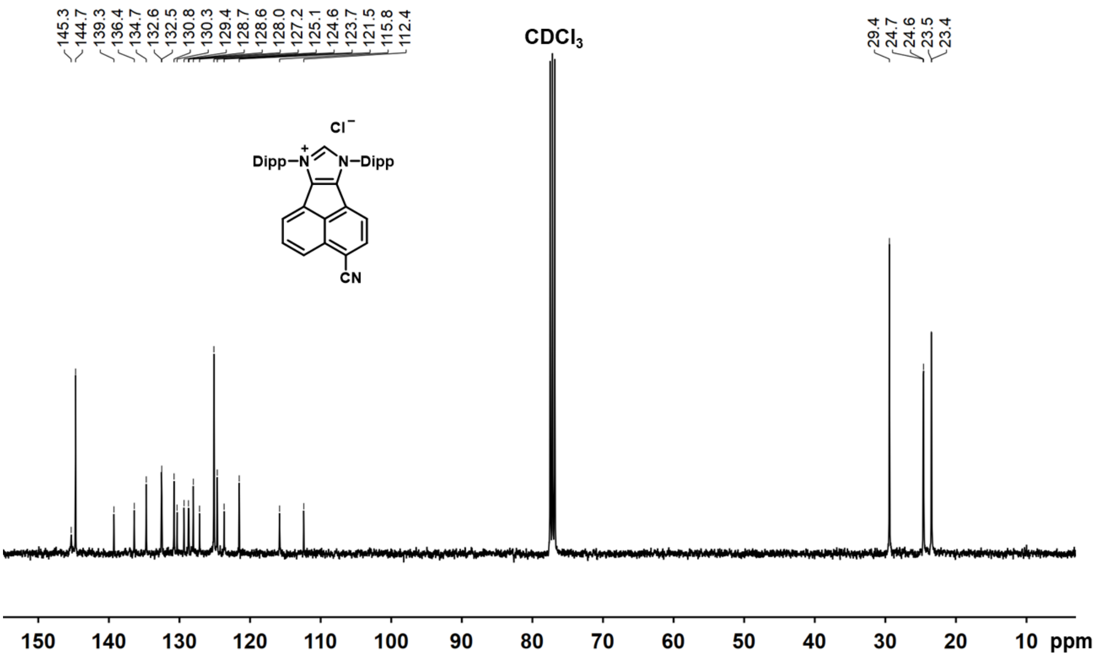

Figure S19. $^{13}C\{^1H\}$ NMR spectrum of IPr(BIAN)^{CN}·HCl (100 MHz, $CDCl_3$).

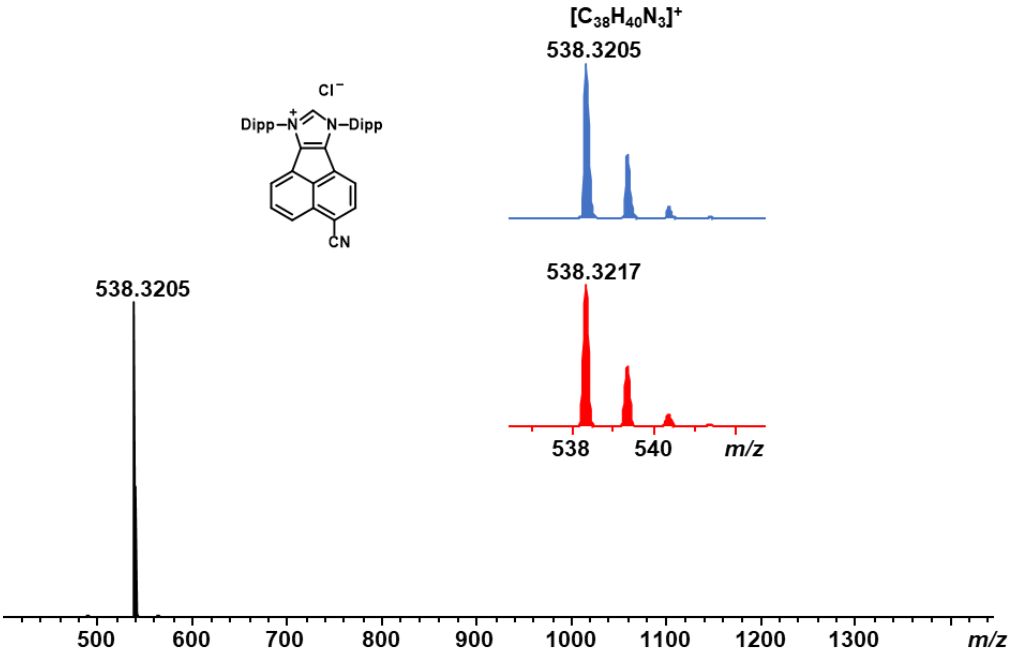

Figure S20. The HRMS spectrum of IPr(BIAN)^{CN}·HCl, inset experimental (blue) and calculated (red) isotope distributions for peaks corresponding to $[C_{38}H_{40}N_3]^+$.

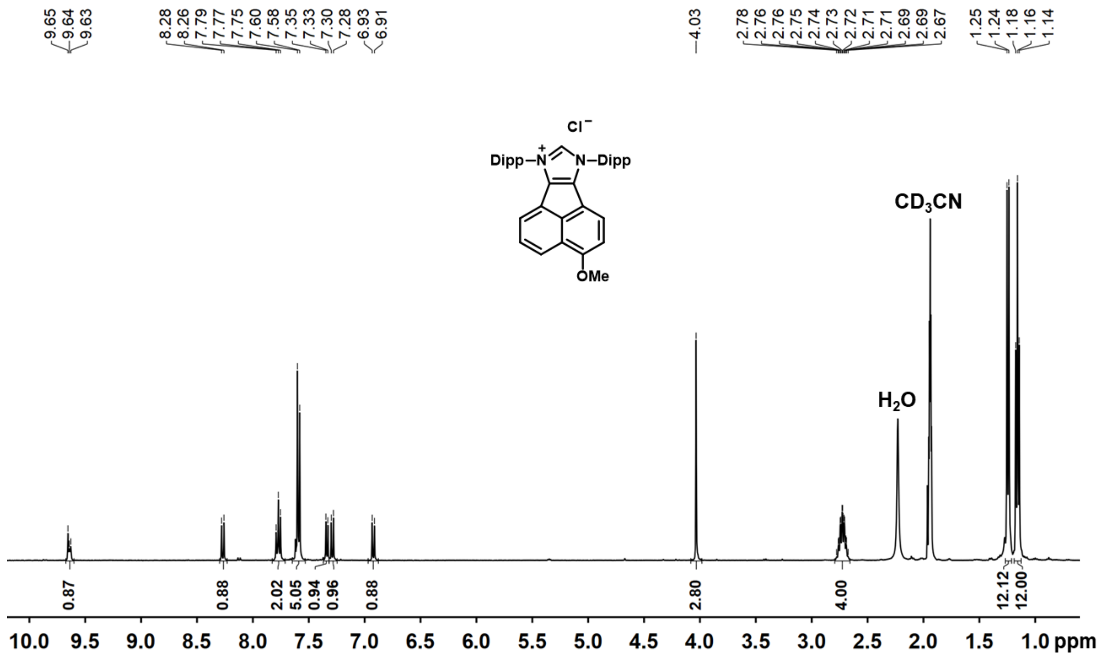

Figure S21. ^{1}H NMR spectrum of IPr(BIAN)^{OMe}·HCl (400 MHz, CD_3CN).

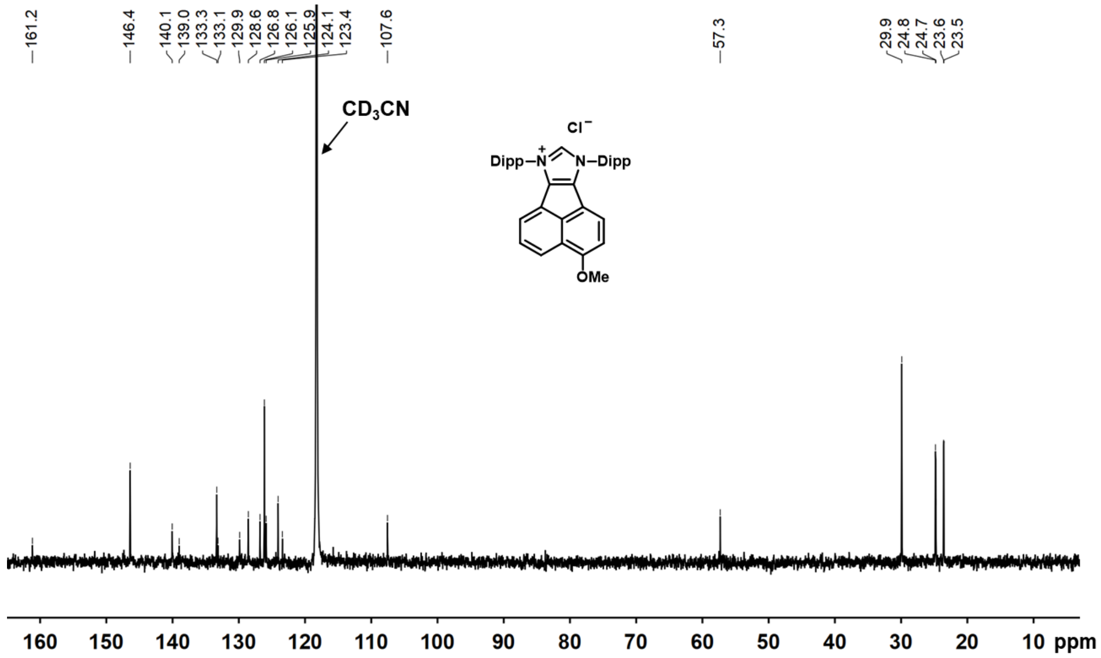

Figure S22. $^{13}C\{^{1}H\}$ NMR spectrum of IPr(BIAN)^{OMe}·HCl (100 MHz, CD_3CN).

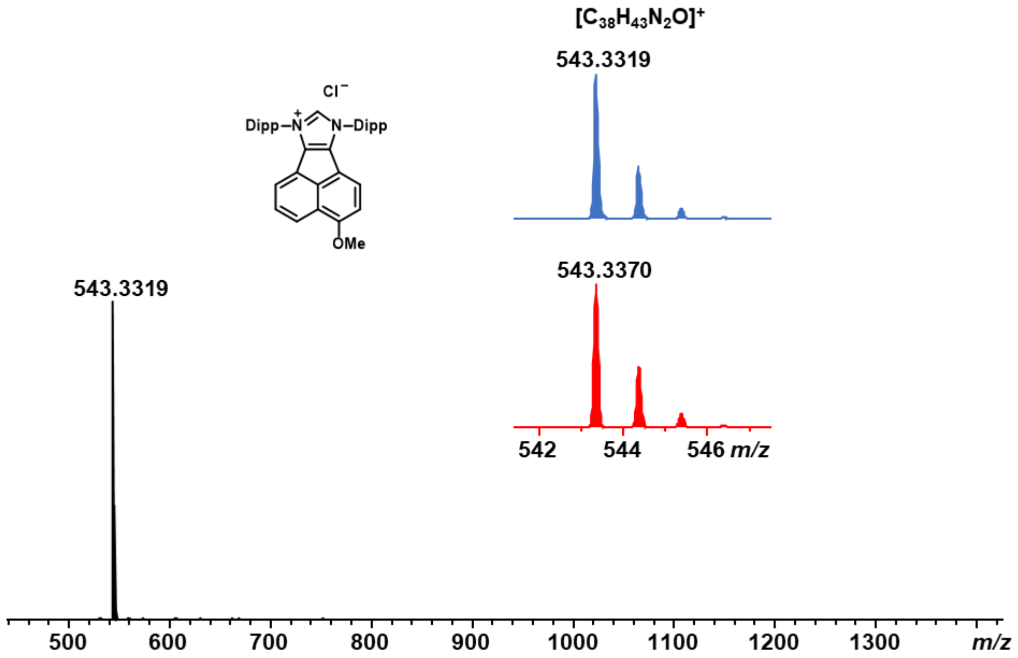

Figure S23. The HRMS spectrum of IPr(BIAN)^{OMe}·HCl, inset experimental (blue) and calculated (red) isotope distributions for peaks corresponding to $[C_{38}H_{43}N_2O]^+$.

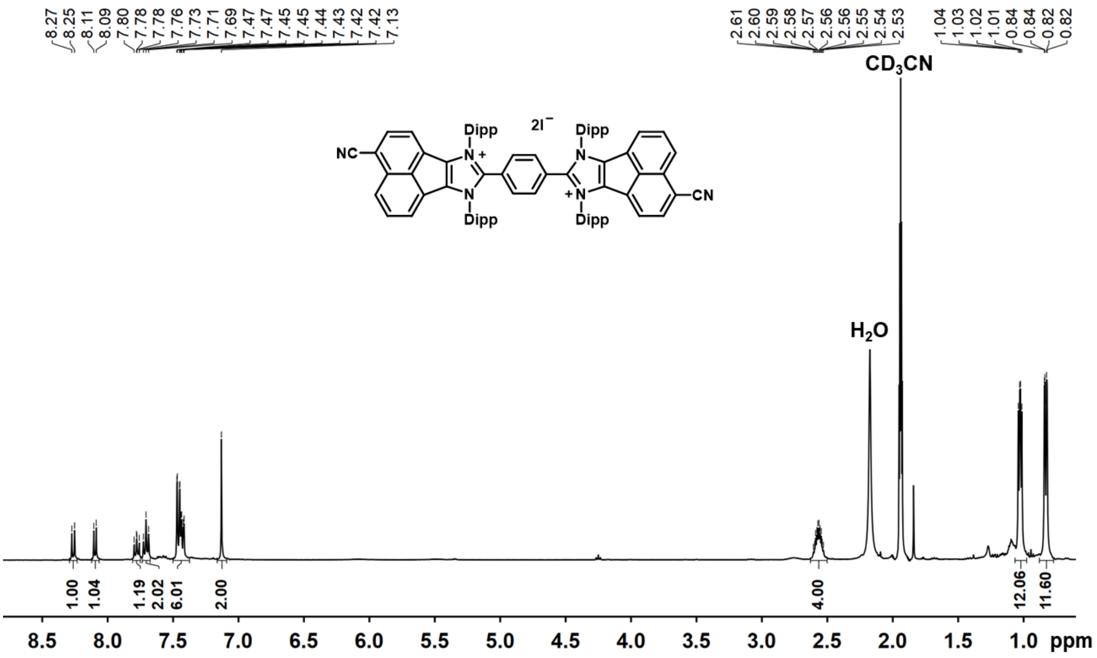

Figure S24. ^{1}H NMR spectrum of $[\mathbf{1d}^{2+}][I^-]_2$ (400 MHz, CD_3CN).

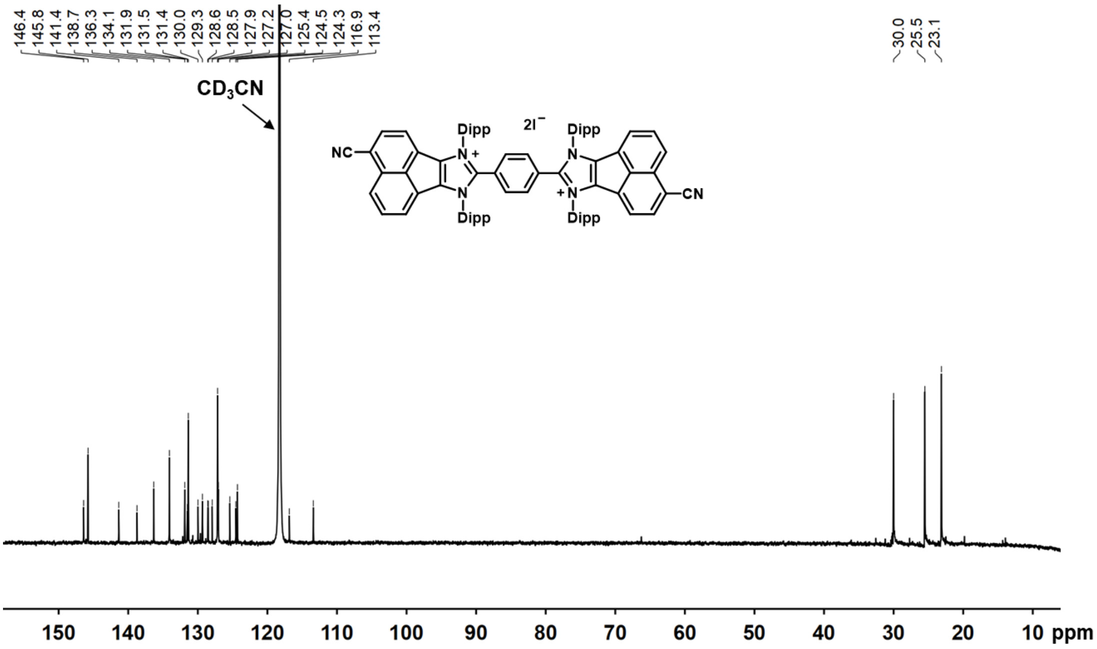

Figure S25. $^{13}C\{^1H\}$ NMR spectrum of [**1d**$^{2+}$][I^-]$_2$ (100 MHz, CD_3CN).

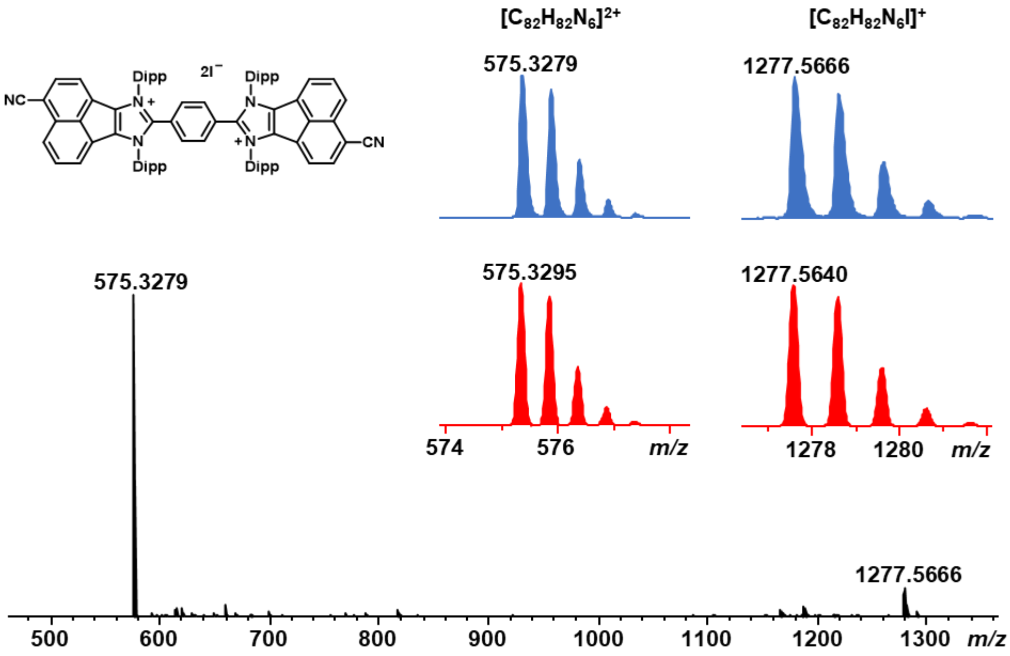

Figure S26. The HRMS spectrum of [**1d**$^{2+}$][I^-]$_2$, inset experimental (blue) and calculated (red) isotope distributions for peaks corresponding to $[C_{82}H_{82}N_6]^{2+}$ and $[C_{82}H_{82}N_6I]^+$.

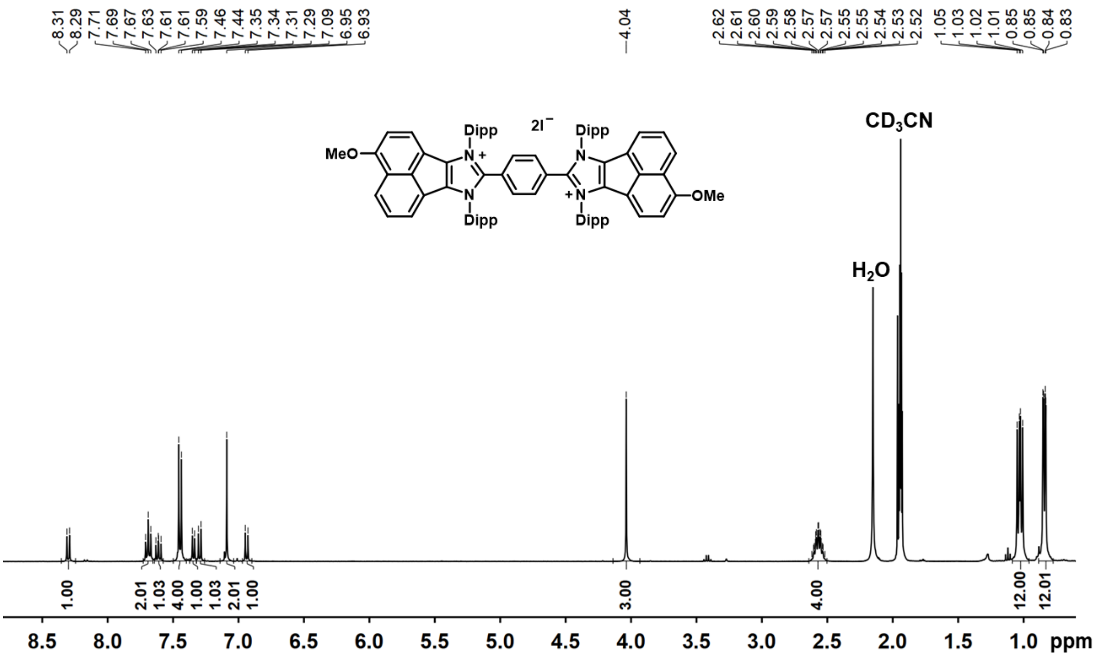

Figure S27. ^{1}H NMR spectrum of [**1e**$^{2+}$][I^{-}]$_2$ (400 MHz, CD_3CN).

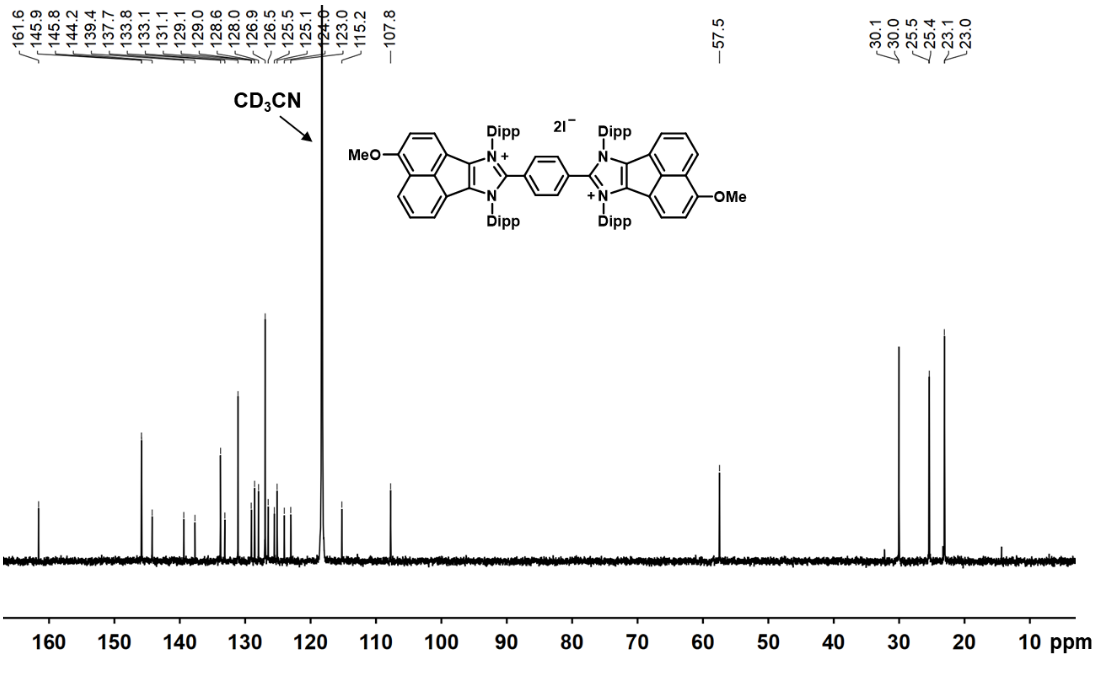

Figure S28. ^{13}C{^{1}H} NMR spectrum of [**1e**$^{2+}$][I^{-}]$_2$ (100 MHz, CD_3CN).

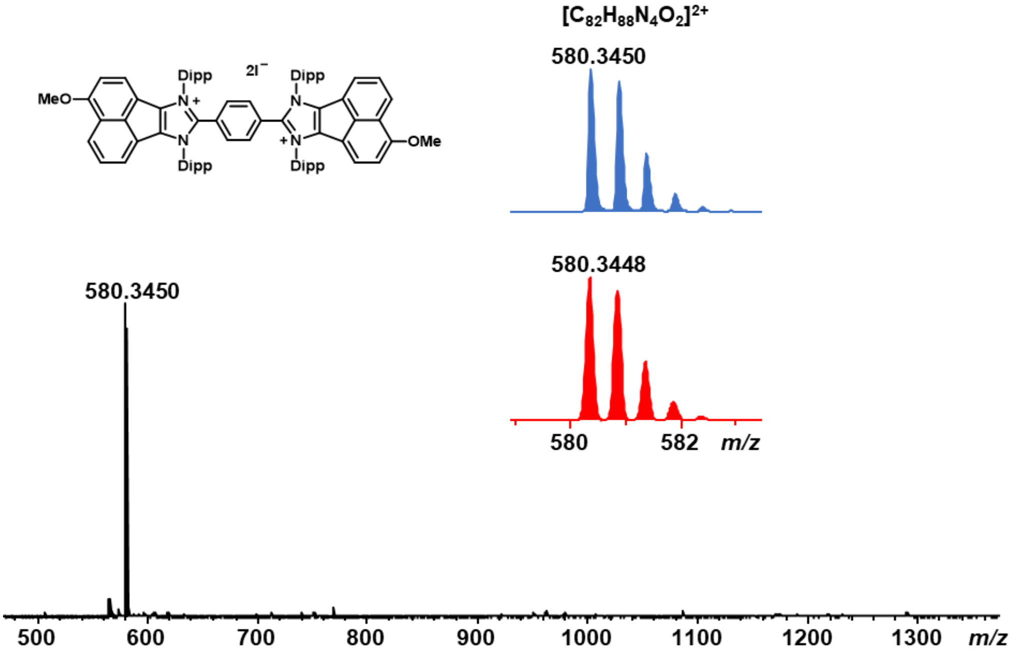

Figure S29. The HRMS spectrum of [**1e**$^{2+}$][I$^-$]$_2$, inset experimental (blue) and calculated (red) isotope distributions for peaks corresponding to [$C_{82}H_{88}N_4O_2$]$^{2+}$.

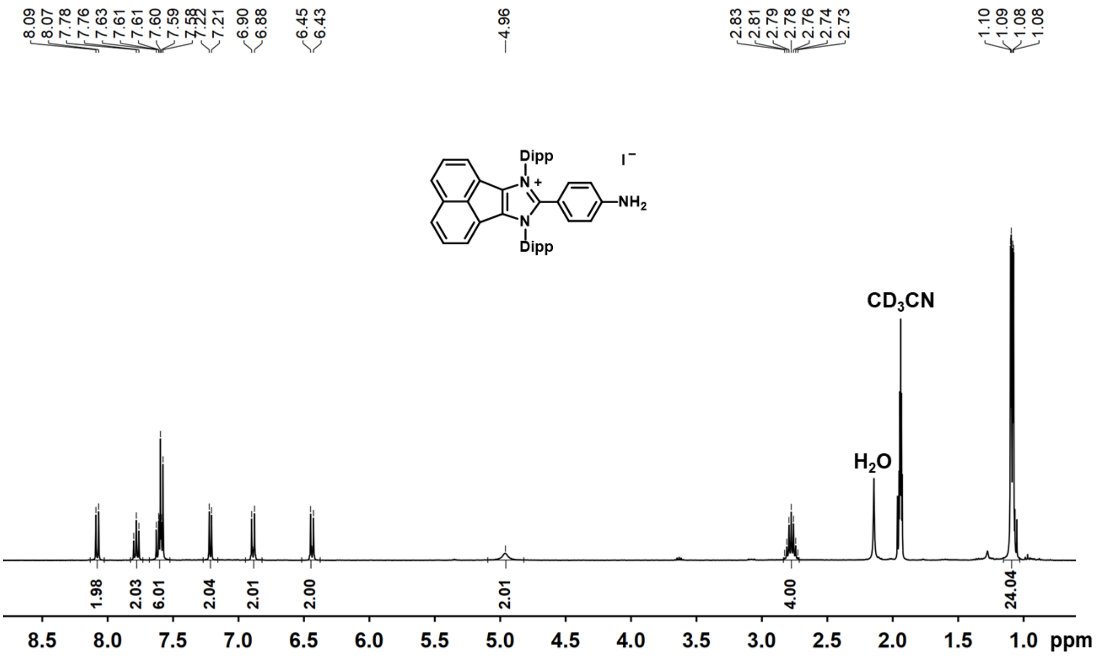

Figure S30. ^{1}H NMR spectrum of [**III**$^+$][I$^-$] (400 MHz, CD_3CN).

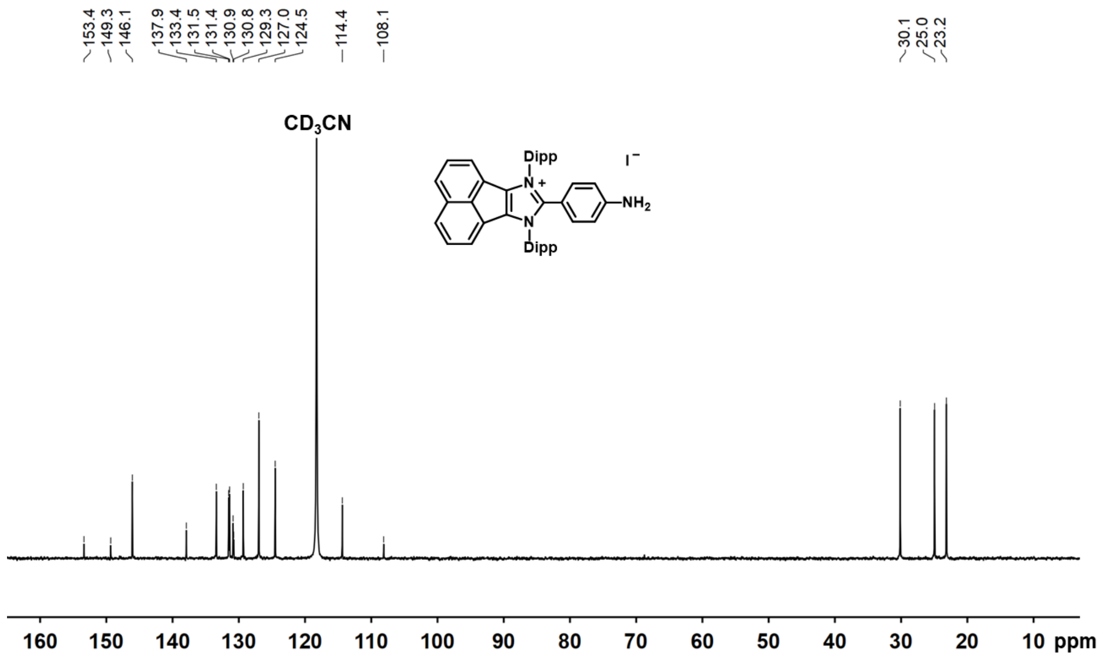

Figure S31. $^{13}C\{^1H\}$ NMR spectrum of [**III**$^+$][I$^-$] (100 MHz, CD_3CN).

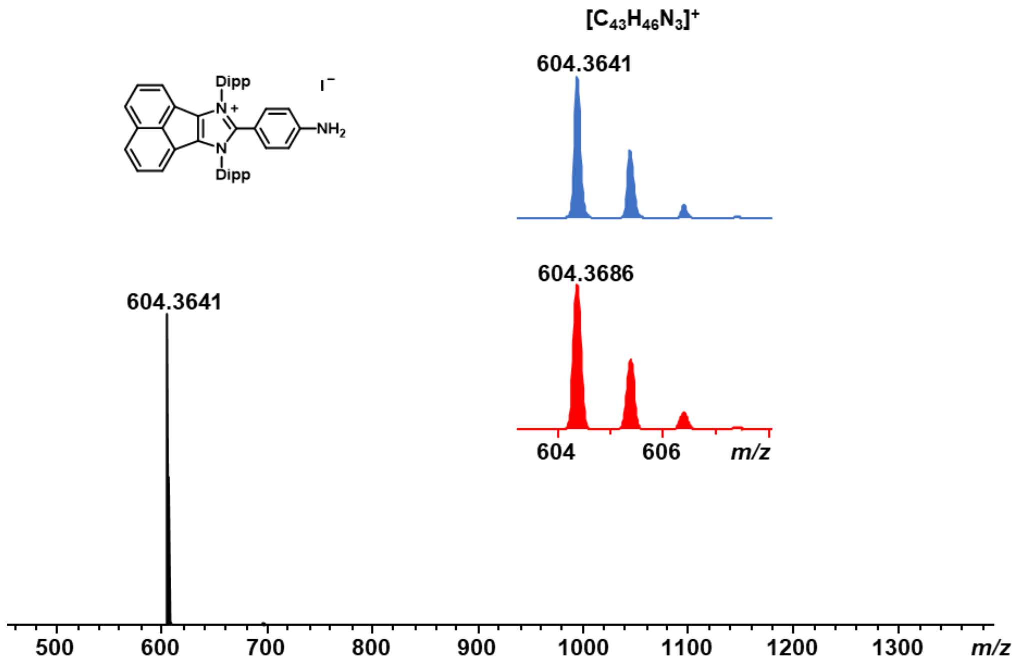

Figure S32. The HRMS spectrum of [**III**$^+$][I$^-$], inset experimental (blue) and calculated (red) isotope distributions for peaks corresponding to $[C_{43}H_{46}N_3]^+$.

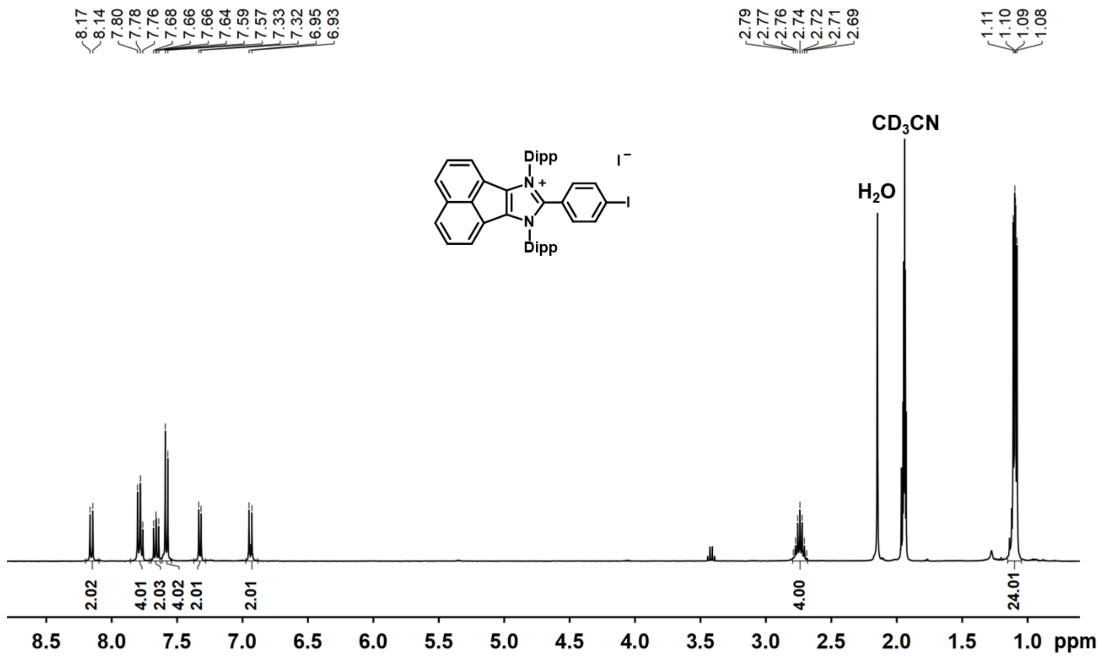

Figure S33. ^{1}H NMR spectrum of [**IV**$^{+}$][I^{-}] (400 MHz, CD_3CN).

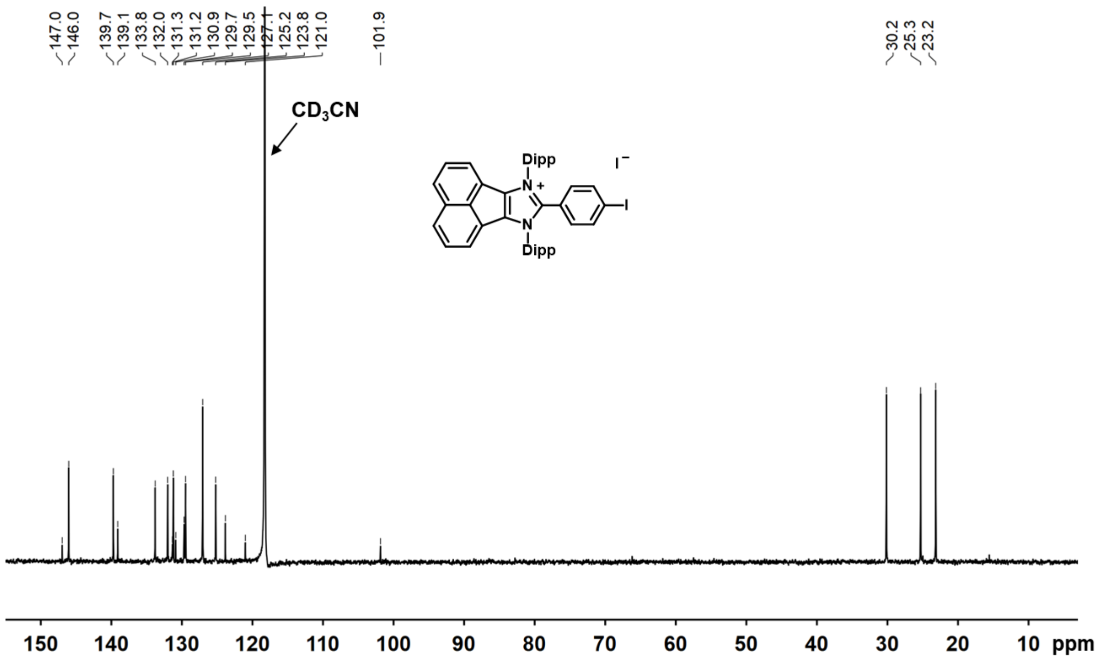

Figure S34. $^{13}C\{^{1}H\}$ NMR spectrum of [**IV**$^{+}$][I^{-}] (100 MHz, CD_3CN).

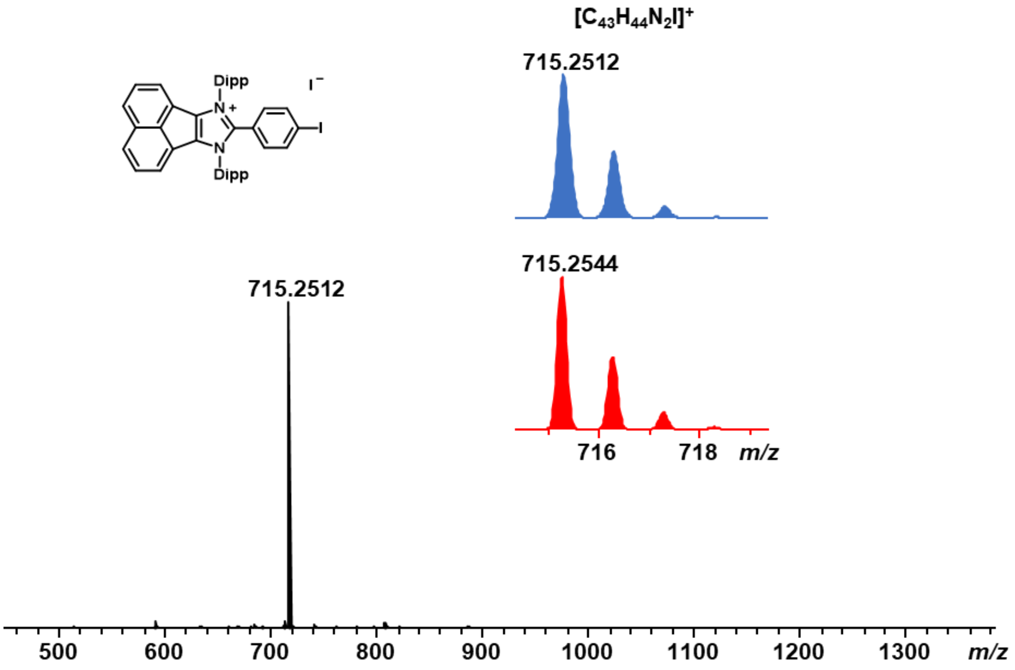

Figure S35. The HRMS spectrum of [**IV**$^+$][I$^-$], inset experimental (blue) and calculated (red) isotope distributions for peaks corresponding to $[C_{43}H_{44}N_2I]^+$.

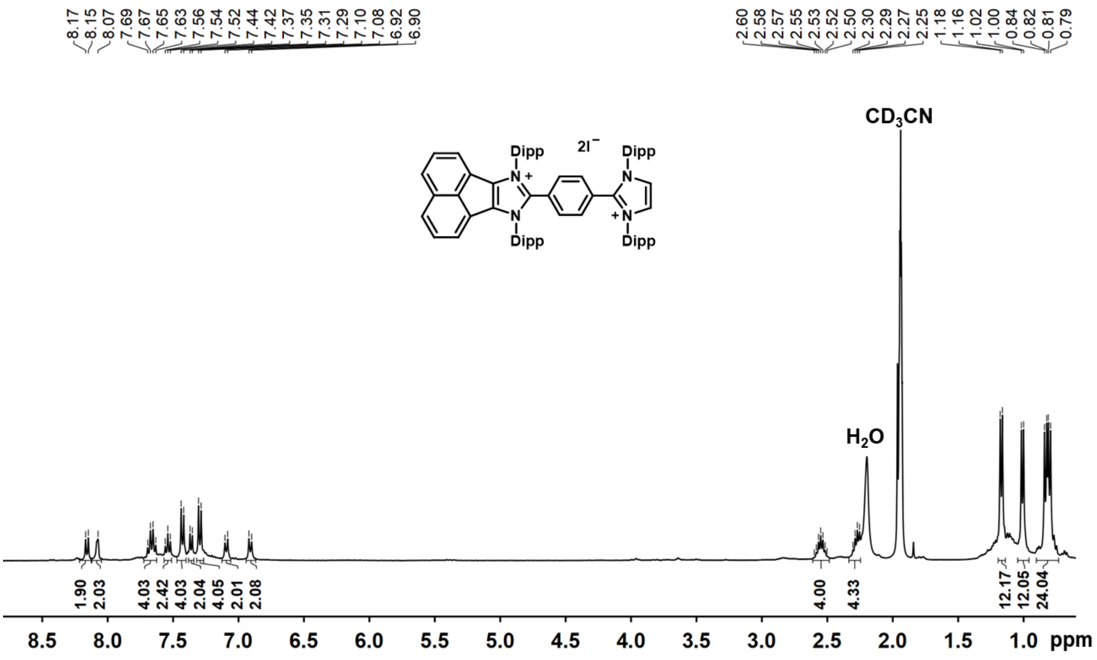

Figure S36. ^{1}H NMR spectrum of [**1f**$^{2+}$][I$^-$]$_2$ (400 MHz, CD_3CN).

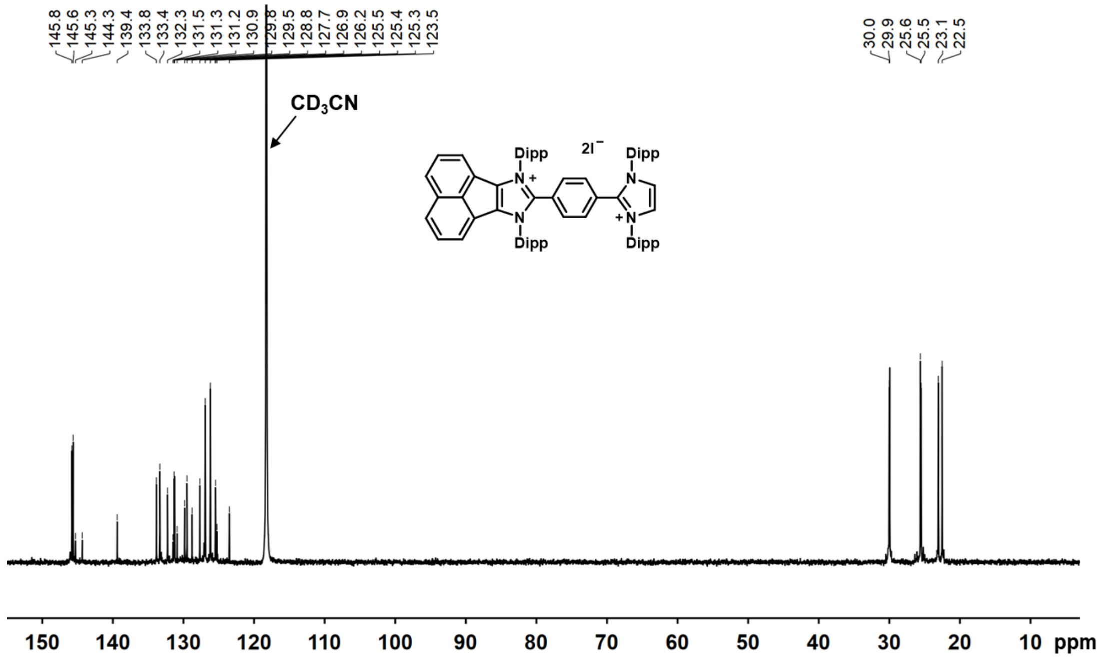

Figure S37. $^{13}C\{^{1}H\}$ NMR spectrum of $[\mathbf{1f}^{2+}][I^{-}]_2$ (100 MHz, CD_3CN).

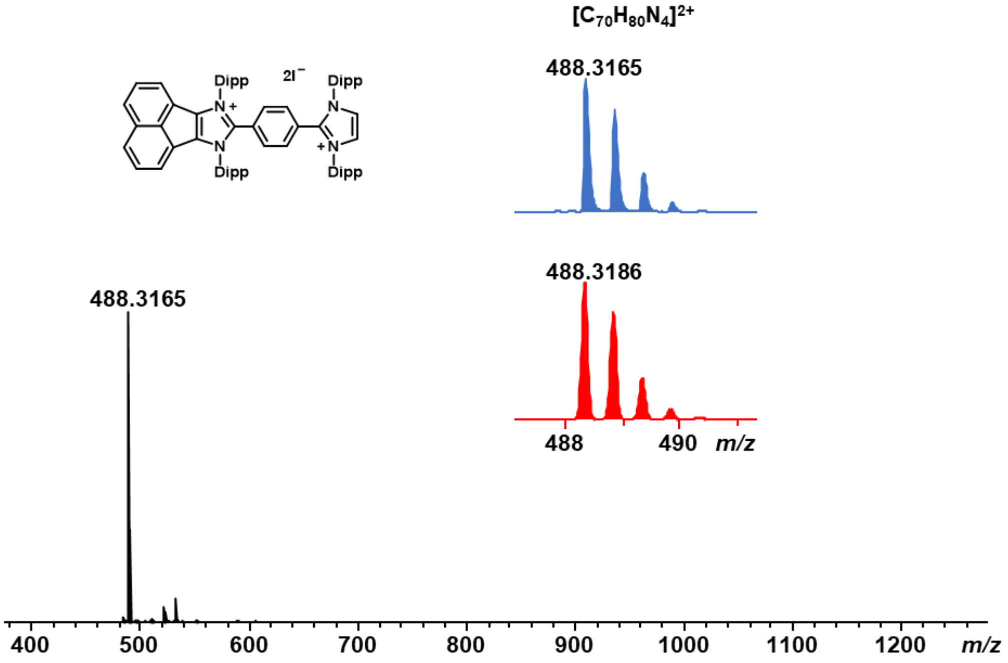

Figure S38. The HRMS spectrum of $[\mathbf{1f}^{2+}][I^{-}]_2$, inset experimental (blue) and calculated (red) isotope distributions for peaks corresponding to $[C_{70}H_{80}N_4]^{2+}$.

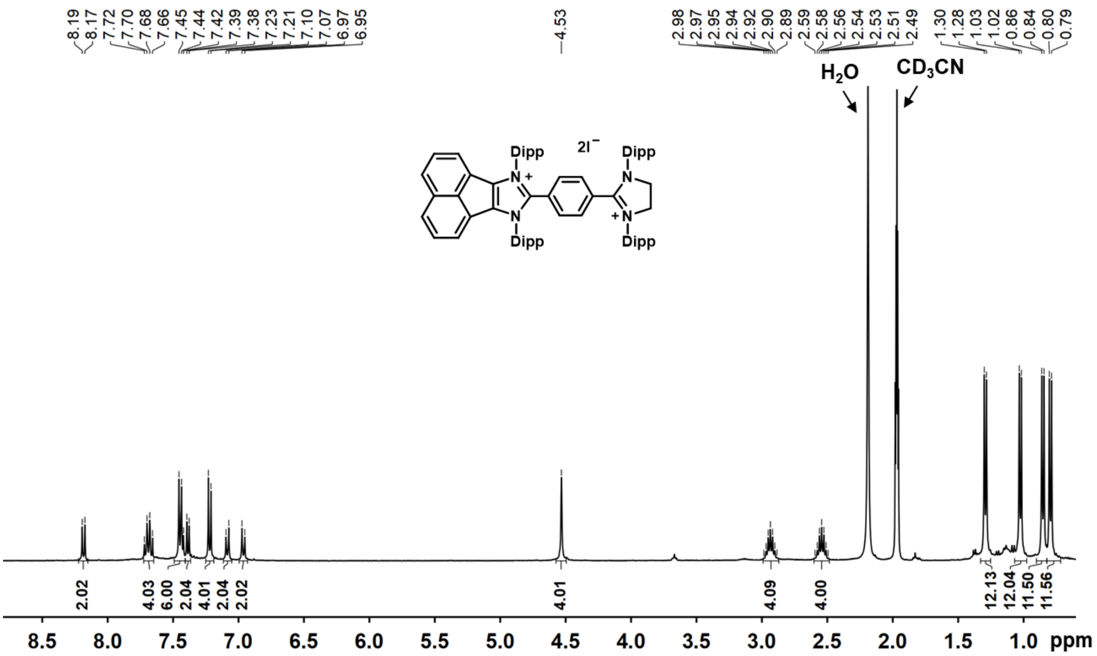

Figure S39. ^{1}H NMR spectrum of [**1g**$^{2+}$][I^{-}]$_2$ (400 MHz, CD_3CN).

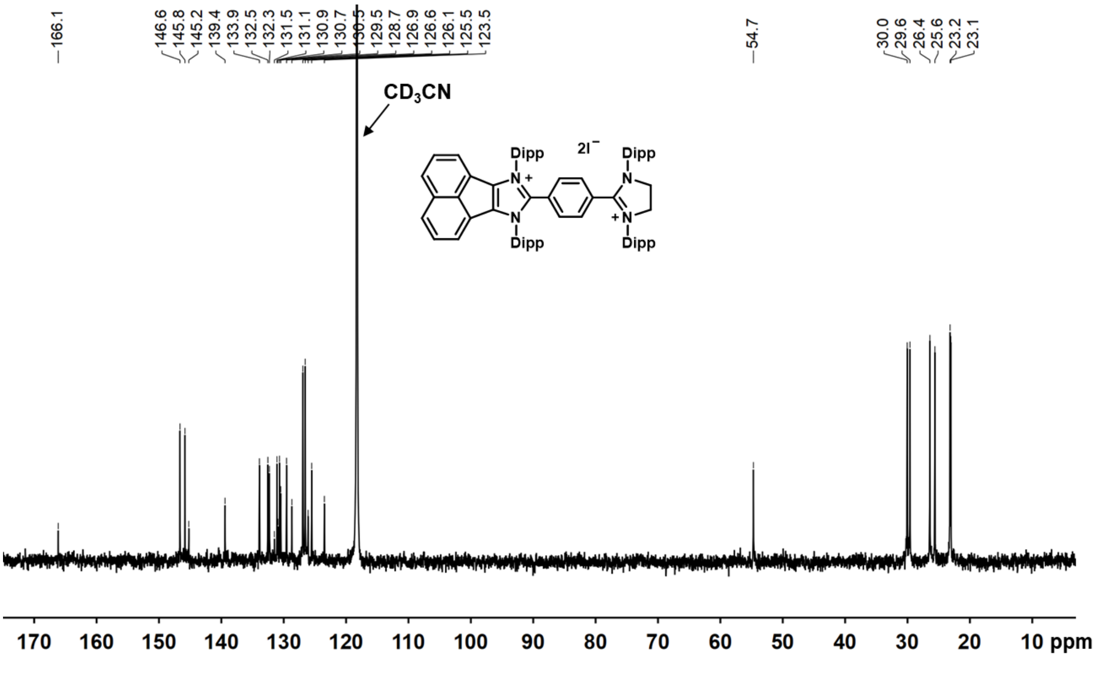

Figure S40. ^{13}C{^{1}H} NMR spectrum of [**1g**$^{2+}$][I^{-}]$_2$ (100 MHz, CD_3CN).

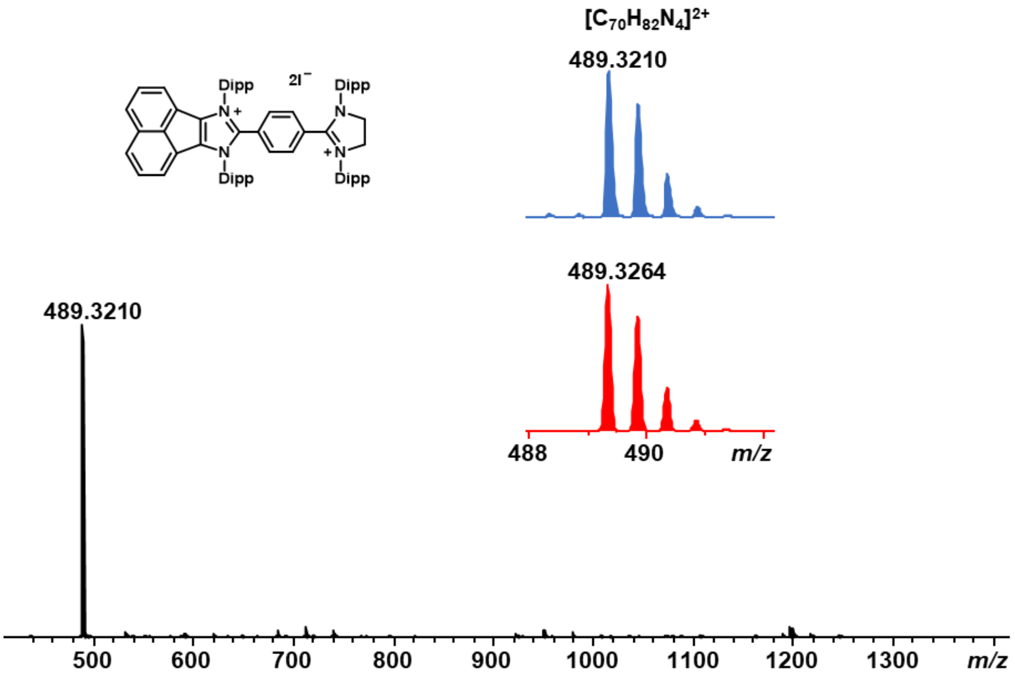

Figure S41. The HRMS spectrum of [**1g**$^{2+}$][I^{-}]$_2$, inset experimental (blue) and calculated (red) isotope distributions for peaks corresponding to [$C_{70}H_{82}N_4$]$^{2+}$.

2. Cyclic voltammetry

Cyclic voltammetry (CV) and differential pulse voltammetry (DPV) experiments were carried out using a CHI660E electrochemical workstation (CH Instruments, Inc). All experiments were carried out under an atmosphere of nitrogen in degassed and anhydrous acetonitrile solution containing Bu_4NPF_6 (0.1 M) at a scan rate of 100 mV s^{-1}. The setup consisted of a glassy carbon working electrode, a platinum wire counter electrode, and a silver wire inserted in a small glass tube fitted with a porous Vycor frit and filled with frit and filled with a $AgNO_3$ solution in acetonitrile. Ferrocene was used as a standard, and all reduction potentials are reported with respect to the $E_{1/2}$ of the Fc/Fc$^+$ redox couple.

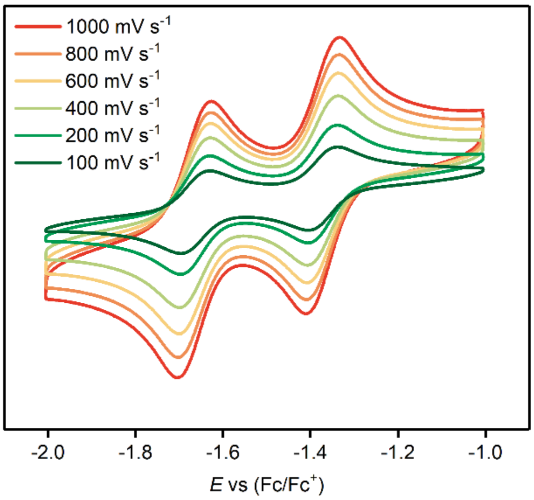

Figure S42. CVs of [**1a**$^{2+}$][I$^-$]$_2$ at different scan rates.

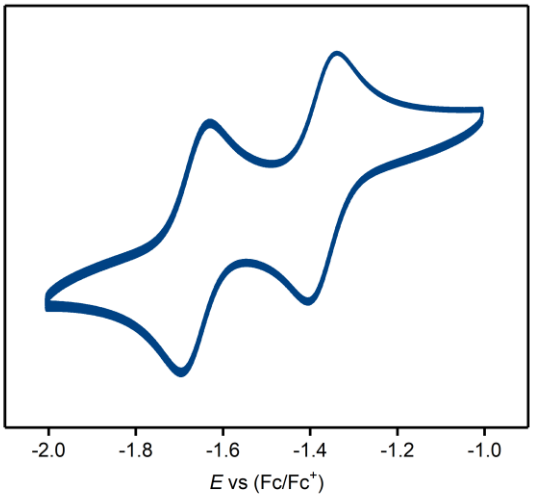

Figure S43. Multicycle CVs of [**1a**$^{2+}$][I$^-$]$_2$ with a scan rate of 100 mV s^{-1}.

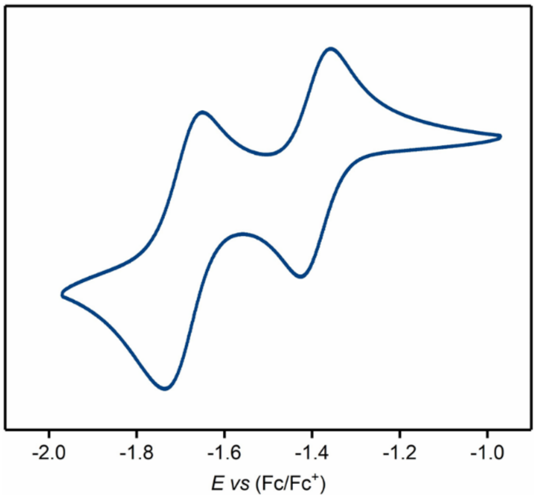

Figure S44. CV of [**1a**$^{2+}$][BF_4^-]$_2$ with a scan rate of 100 mV s^{-1}.

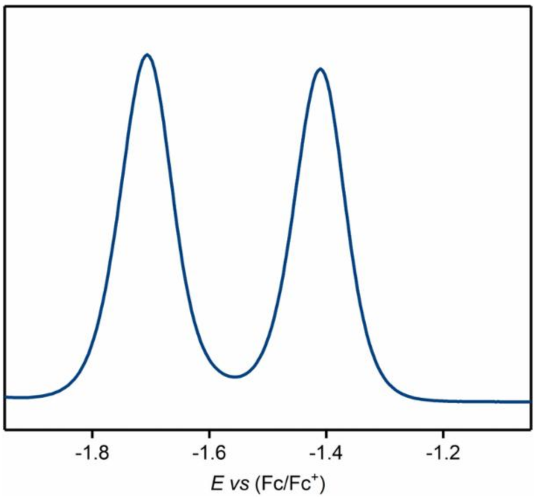

Figure S45. DPV of [**1a**$^{2+}$][BF_4^-]$_2$ in acetonitrile.

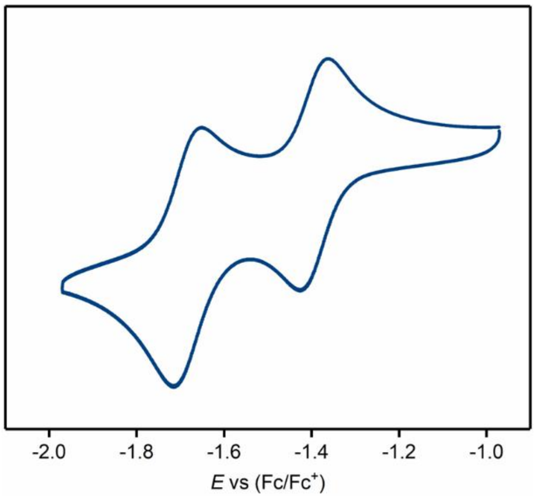

Figure S46. CV of [**1a**$^{2+}$][PF_6^-]$_2$ with a scan rate of 100 mV s^{-1}.

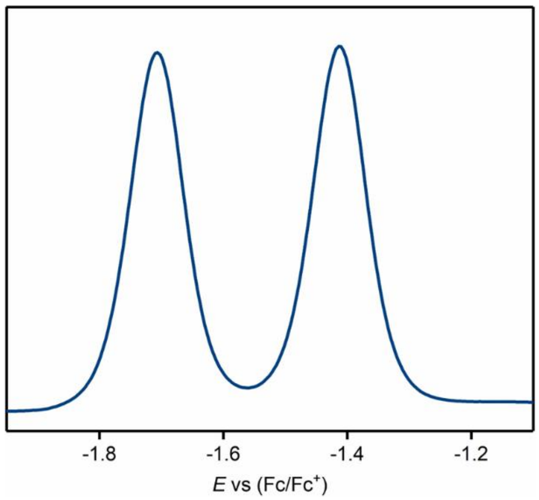

Figure S47. DPV of [**1a**$^{2+}$][PF_6^-]$_2$ in acetonitrile.

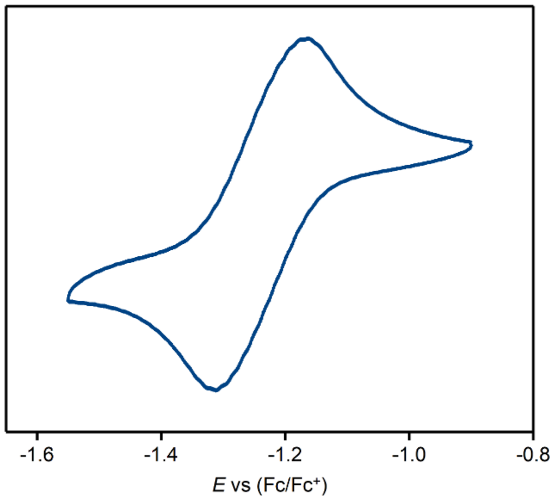

Figure S48. CV of [**1d**$^{2+}$][I^-]$_2$ with a scan rate of 100 mV s^{-1}.

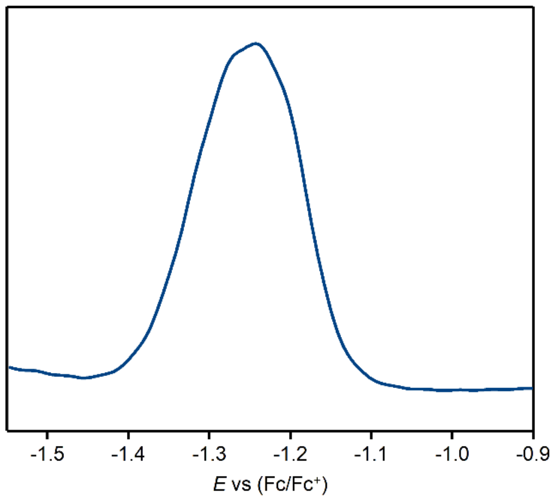

Figure S49. DPV of [**1d**$^{2+}$][I^-]$_2$ in acetonitrile.

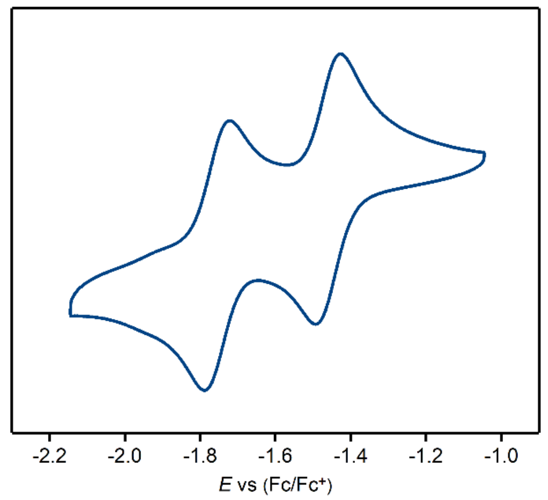

Figure S50. CV of [**1e**$^{2+}$][I^{-}]$_2$ with a scan rate of 100 mV s^{-1}.

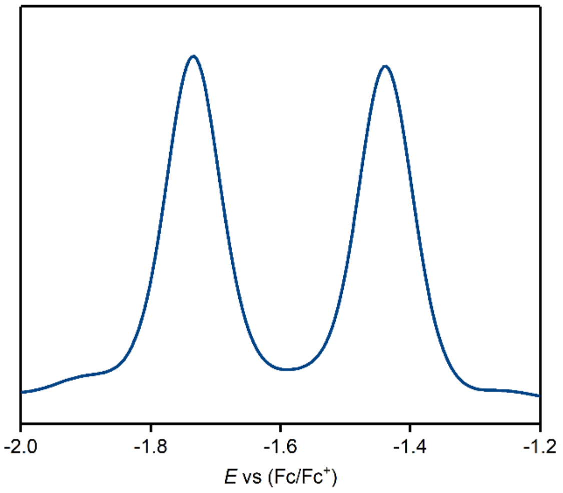

Figure S51. DPV of [**1e**$^{2+}$][I^{-}]$_2$ in acetonitrile.

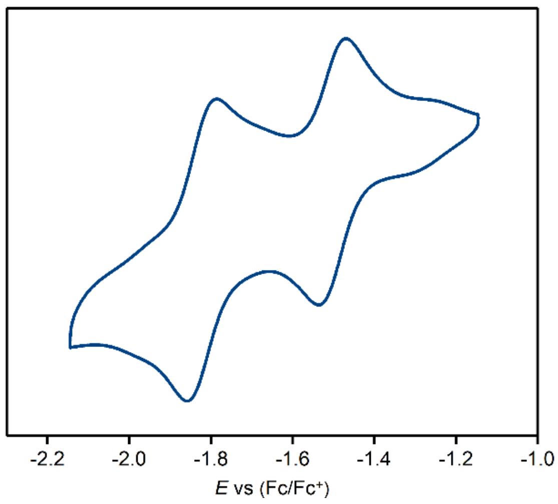

Figure S52. CV of [**1f**$^{2+}$][I^{-}]$_2$ with a scan rate of 100 mV s^{-1}.

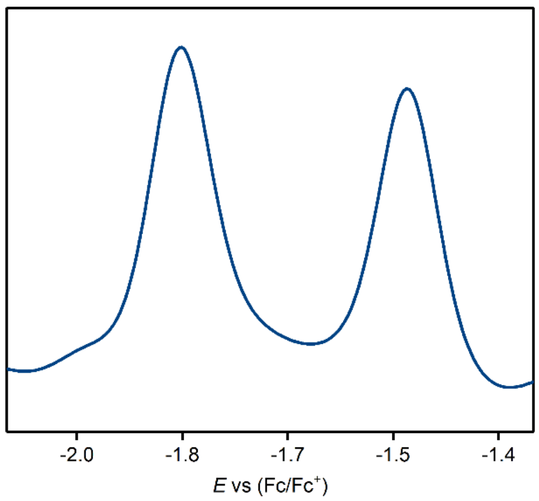

Figure S53. DPV of [**1f**$^{2+}$][I^{-}]$_2$ in acetonitrile.

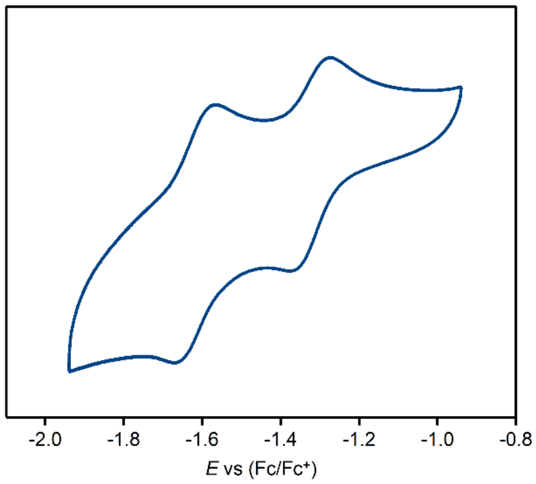

Figure S54. CV of [**1g**$^{2+}$][I^{-}]$_2$ with a scan rate of 100 mV s^{-1}.

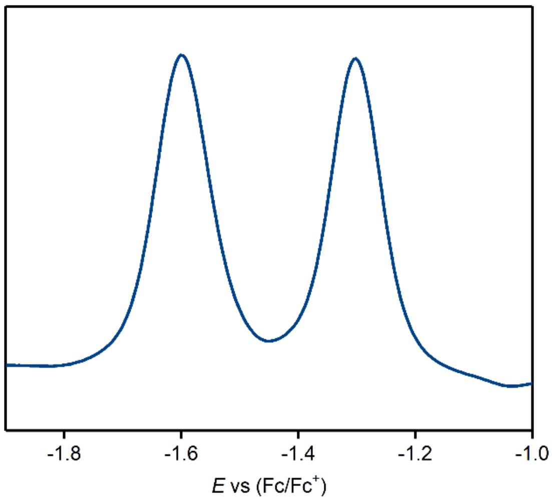

Figure S55. DPV of [**1g**$^{2+}$][I^{-}]$_2$ in acetonitrile.

3. Crystallographic details

Diffraction data of compounds were collected with a Bruker APEX-II CCD diffractometer. Raw data collection and processing were performed with APEX III software package. The data were corrected for absorption using the SADABS program [7]. Using the program Olex2, the structures were solved with the olex2, solve structure solution program using Charge Flipping and refined with the ShelXL refinement package using Least Squares minimization [8,9]. All hydrogen atoms were treated as idealized contributions, and non-hydrogen atoms were refined anisotropically.

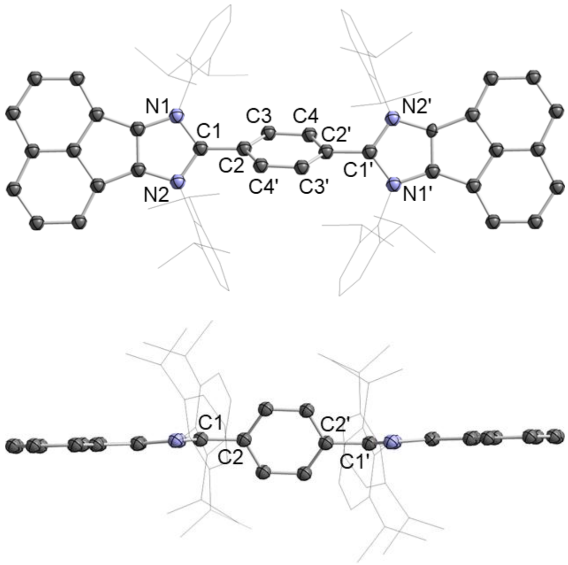

Figure S56. Solid-state molecular structure of [**1a**$^{2+}$][I^{-}]$_2$. Hydrogen atoms and counter anion were omitted for clarity. Selected bond lengths [Å] and bond angles [°]: N1–C1 1.354(3), N2–C1 1.356(3), C1–C2 1.465(4), C2–C3 1.388(3), C3–C4 1.381(4), C4–C2' 1.393(3) and N1–C1–N2 109.3(19), C3–C2–C4' 120.5(2). Selected torsion angles [°]: N1–C1–C2–C4' 116.4(3), N2–C1–C2–C3 121.1(3), N1–C1–C2–C3 64.3(3), N2–C1–C2–C4' 58.2(3).

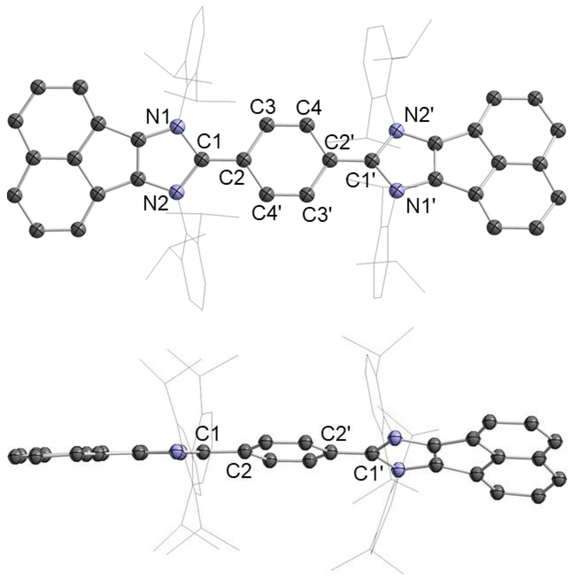

Figure S57. Solid-state molecular structure of [**1a**$^{\bullet+}$][I^-]. Hydrogen atoms and counter anion were omitted for clarity. Selected bond lengths [Å] and bond angles [°]: N1–C1 1.379(8), N2–C1 1.393(9), C1–C2 1.414(9), C2–C3 1.433(9), C3–C4 1.348(9), C4–C2' 1.414(9) and N1–C1–N2 105.9(5), C3–C2–C4' 113.9(5). Selected torsion angles [°]: N1–C1–C2–C4' 165.7(6), N2–C1–C2–C3 166.2(6), N1–C1–C2–C3 14.9(10), N2–C1–C2–C4' 13.2(10).

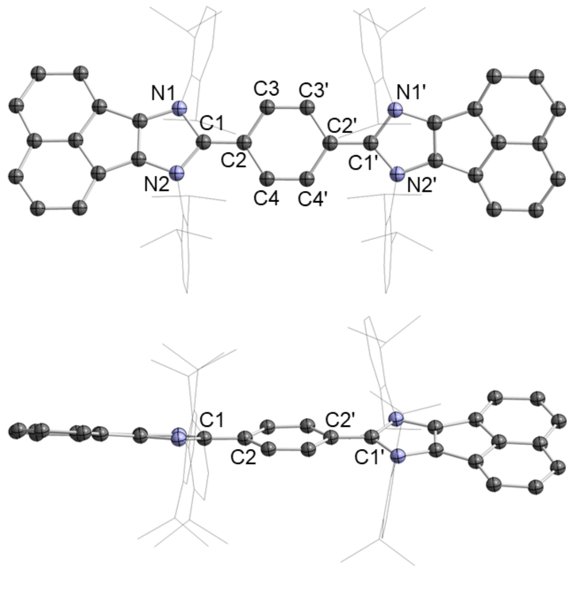

Figure S58. Solid-state molecular structure of **1a**. Hydrogen atoms and counter anion were omitted for clarity. Selected bond lengths [Å] and bond angles [°]: N1–C1 1.430(5), N2–C1 1.411(5), C1–C2 1.376(5), C2–C3 1.430(5), C3–C3' 1.352(8), C2–C4 1.449(5) and N1–C1–N2 105.2(3), C3–C2–C4 114.2(3). Selected torsion angles [°]: N1–C1–C2–C4 167.8(4), N2–C1–C2–C3 163.2(4), N1–C1–C2–C3 12.4(6), N2–C1–C2–C4 16.6(7).

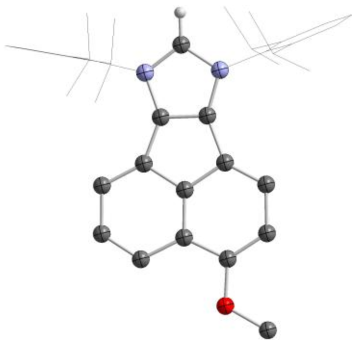

Figure S59. Solid-state molecular structure of IPr(BIAN)^{OMe}·HCl. Hydrogen atoms and counter anion were omitted for clarity.

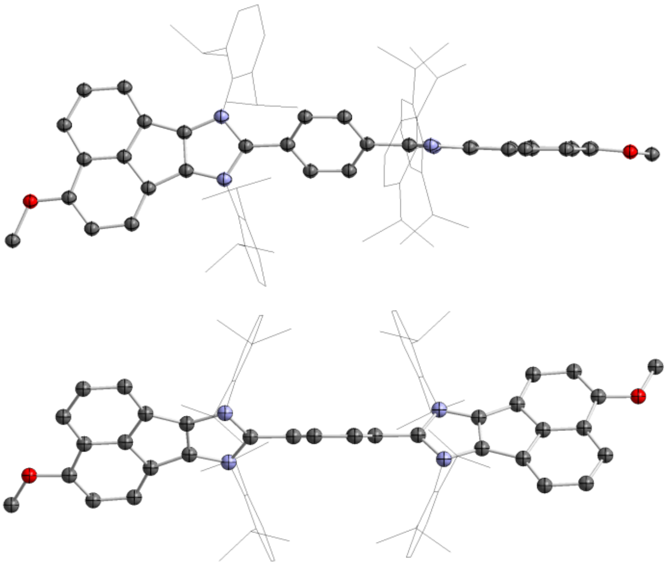

Figure S60. Solid-state molecular structure of [**1e**$^{2+}$][I^{-}]$_2$. Hydrogen atoms and counter anion were omitted for clarity.

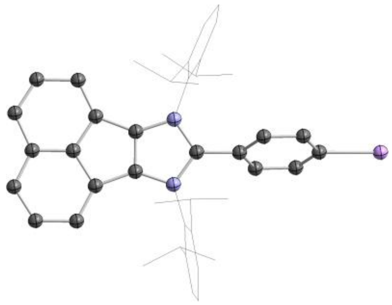

Figure S61. Solid-state molecular structure of [**IV**$^{+}$][I^{-}]. Hydrogen atoms and counter anion were omitted for clarity.

Table S1. Selected bond lengths [Å] and angles [°] determined by X-ray crystallography.

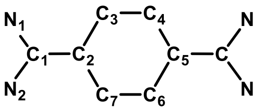

	$[\mathbf{1a}^{2+}][I^-]_2$	$[\mathbf{1a}^{\bullet+}][I^-]$	**1a**
C1–C2	1.465(4)	1.414(9)	1.376(5)
C2–C3	1.388(3)	1.433(9)	1.430(5)
C2–C7	1.393(3)	1.414(9)	1.449(5)
C3–C4	1.381(4)	1.348(9)	1.352(8)
N1–C1–N2	109.3(19)	105.9(5)	105.2(3)
N1–C1–C2–C7	116.4(3)	165.7(6)	167.8(4)

Table S2. X-Ray crystallographic details of [**1a**$^{2+}$][I$^-$]$_2$.

Compound	[**1a**$^{2+}$][I$^-$]$_2$·2CH$_3$CN
Empirical formula	$C_{84}H_{90}N_6I_2$
Formula weight	1437.41
Radiation / λ	Mo Kα / (0.71073 Å)
Temperature/K	166.0
Crystal system	monoclinic
Space group	$P2_1/c$
a/Å	15.5429(5)
b/Å	20.5071(6)
c/Å	11.7942(4)
α/°	90
β/°	107.8200(10)
γ/°	90
V/Å^3	3578.9(2)
Z	2
P calcg/cm^3	1.334
μ/mm^{-1}	0.928
F(000)	1484.0
2Θ range for data collection/°	4.834 to 50.776
Index ranges	$-18 \leq h \leq 18$, $-24 \leq k \leq 24$, $-14 \leq l \leq 14$
Independent reflections	6562 [R_{int} = 0.0614]
Data/restraints/parameters	6562/0/424
Goodness-of-fit on F^2	1.050
Final R indexes [I>=2σ (I)]	R_1 = 0.0329, wR_2 = 0.0659
Final R indexes [all data]	R_1 = 0.0478, wR_2 = 0.0735
Largest diff. peak/hole / e Å^{-3}	0.52/-0.4
CCDC number	2431383

Table S3. X-Ray crystallographic details of [**1a**$^{\bullet+}$][I^-].

Compound	[**1a**$^{\bullet+}$][I^-]
Empirical formula	$C_{80}H_{84}N_4I$
Formula weight	1228.41
Radiation / λ	Cu Kα / (1.54178 Å)
Temperature/K	220.0
Crystal system	monoclinic
Space group	*C*2/*c*
a/Å	27.646(19)
b/Å	14.559(10)
c/Å	22.55(3)
α/°	90
β/°	124.62(3)
γ/°	90
V/Å^3	7470(11)
Z	4
P calcg/cm^3	1.092
μ/mm^{-1}	3.676
F(000)	2580.0
2Θ range for data collection/°	7.208 to 118.336
Index ranges	-24 ≤ h ≤ 30, -14 ≤ k ≤ 16, -19 ≤ l ≤ 25
Independent reflections	5145 [R_{int} = 0.0474]
Data/restraints/parameters	5145/90/384
Goodness-of-fit on F^2	0.965
Final *R* indexes [I>=2σ (I)]	R_1 = 0.1526, wR_2 = 0.3921
Final *R* indexes [all data]	R_1 = 0.1732, wR_2 = 0.4119
Largest diff. peak/hole / e Å^{-3}	0.93/-0.62
CCDC number	2431385

Table S4. X-Ray crystallographic details of **1a**.

Compound	**1a**·$C_6H_5CH_3$
Empirical formula	$C_{87}H_{92}N_4$
Formula weight	1193.64
Radiation / λ	Mo Kα / (0.71073 Å)
Temperature/K	207.0
Crystal system	tetragonal
Space group	*I*-42*d*
a/Å	20.941(2)
b/Å	20.941(2)
c/Å	32.261(4)
α/°	90
β/°	90
γ/°	90
V/Å^3	14148(4)
Z	8
P calcg/cm^3	1.121
μ/mm^{-1}	0.064
F(000)	5136.0
2Θ range for data collection/°	5.05 to 52.81
Index ranges	$-26 \leq h \leq 18$, $-20 \leq k \leq 26$, $-30 \leq l \leq 40$
Independent reflections	7051 [R_{int} = 0.0601]
Data/restraints/parameters	7051/57/453
Goodness-of-fit on F^2	1.035
Final R indexes [I>=2σ (I)]	R_1 = 0.0550, wR_2 = 0.1171
Final R indexes [all data]	R_1 = 0.1181, wR_2 = 0.1447
Largest diff. peak/hole / e Å^{-3}	0.19/-0.26
CCDC number	2431386

Table S5. X-Ray crystallographic details of IPr(BIAN)^{OMe}·HCl.

Compound	IPr(BIAN)^{OMe}·HCl·3CHCl$_3$
Empirical formula	$C_{41}H_{46}N_2Cl_{10}O$
Formula weight	937.30
Radiation / λ	Cu Kα / (1.54178 Å)
Temperature/K	240.0
Crystal system	orthorhombic
Space group	*Pnma*
a/Å	23.6124(14)
b/Å	14.2593(9)
c/Å	13.8862(9)
α/°	90
β/°	90
γ/°	90
V/Å^3	4675.4(5)
Z	4
P calcg/cm^3	1.332
μ/mm^{-1}	5.712
F(000)	1936.0
2Θ range for data collection/°	7.386 to 133.392
Index ranges	$-28 \leq h \leq 28$, $-16 \leq k \leq 16$, $-16 \leq l \leq 16$
Independent reflections	4293 [R_{int} = 0.0520]
Data/restraints/parameters	4293/0/284
Goodness-of-fit on F^2	1.065
Final R indexes [I>=2σ (I)]	R_1 = 0.0737, wR_2 = 0.2040
Final R indexes [all data]	R_1 = 0.0782, wR_2 = 0.2087
Largest diff. peak/hole / e Å^{-3}	0.66/-0.73
CCDC number	2515073

Table S6. X-Ray crystallographic details of [**1e**$^{2+}$][I^{-}]$_2$.

Compound	[**1e**$^{2+}$][I^{-}]$_2$·3CH$_3$CN
Empirical formula	$C_{88}H_{97}N_7I_2O_2$
Formula weight	1538.52
Radiation / λ	Cu Kα / (1.54178 Å)
Temperature/K	240.0
Crystal system	monoclinic
Space group	*C2/c*
a/Å	23.736(2)
b/Å	16.6878(16)
c/Å	23.514(3)
α/°	90
β/°	117.813(4)
γ/°	90
V/Å^3	8238.2(15)
Z	4
P calcg/cm^3	1.240
μ/mm^{-1}	6.370
F(000)	3184.0
2Θ range for data collection/°	6.766 to 133.256
Index ranges	$-28 \leq h \leq 28$, $-13 \leq k \leq 19$, $-27 \leq l \leq 20$
Independent reflections	7147 [R_{int} = 0.0645]
Data/restraints/parameters	7147/15/444
Goodness-of-fit on F^2	1.084
Final R indexes [I>=2σ (I)]	R_1 = 0.1315, wR_2 = 0.3786
Final R indexes [all data]	R_1 = 0.1426, wR_2 = 0.3838
Largest diff. peak/hole / e Å^{-3}	2.35/-1.13
CCDC number	2515071

Table S7. X-Ray crystallographic details of [**IV**$^+$][I$^-$].

Compound	[**IV**$^+$][I$^-$]·$2C_4H_8O·0.5C_6H_{14}$
Empirical formula	$C_{54}H_{67}N_2I_2O_2$
Formula weight	1029.89
Radiation / λ	Cu Kα / (1.54178 Å)
Temperature/K	240.0
Crystal system	triclinic
Space group	*P-1*
a/Å	12.0041(12)
b/Å	12.3042(14)
c/Å	19.956(2)
α/°	100.029(6)
β/°	90.949(5)
γ/°	119.074(5)
V/Å^3	2519.2(5)
Z	2
P calcg/cm^3	1.358
μ/mm^{-1}	10.103
F(000)	1054.0
2Θ range for data collection/°	4.528 to 130.554
Index ranges	$-14 \leq h \leq 14$, $-14 \leq k \leq 14$, $-23 \leq l \leq 23$
Independent reflections	8484 [R_{int} = 0.0660]
Data/restraints/parameters	8484/70/535
Goodness-of-fit on F^2	1.083
Final R indexes [I>=2σ (I)]	R_1 = 0.0574, wR_2 = 0.1637
Final R indexes [all data]	R_1 = 0.0752, wR_2 = 0.1788
Largest diff. peak/hole / e Å^{-3}	0.97/-1.04
CCDC number	2515072

4. EPR spectra

EPR measurement at X-band were carried out using a Bruker ELEXSYS-II E500 CW EPR spectrometer. For temperature control (300-100 K), EPR spectrometer was equipped with custom-made nitrogen flow system. The spectral analysis was performed using MATLAB 9.14.0.2006163 (R2023a) and the Curve Fitting toolbox. The spectral simulations were performed using MATLAB 9.14.0.2006163 (R2023a) and the EasySpin 6.0.5 [10].

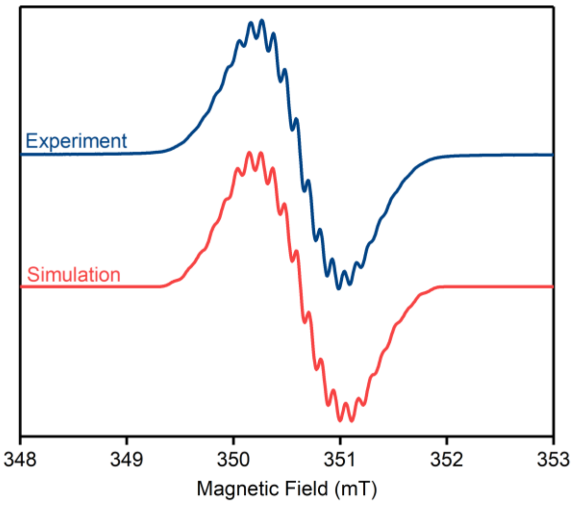

Figure S62. Experimental (blue) and simulated (red) EPR spectra of [**1a**$^{\bullet+}$][I^{-}] in CH_2Cl_2 solution at room temperature (c = 1.0 mM). Microwave freq. 9.83 GHz, powder = 2.0 mW, amp. 0.1 mT. Hyperfine coupling constants: g = 2.0032, $A_{iso}(4{*}^{14}N)$ = 2.2 G, $A_{iso}(4{*}^{1}H)$ = 1.2 G.

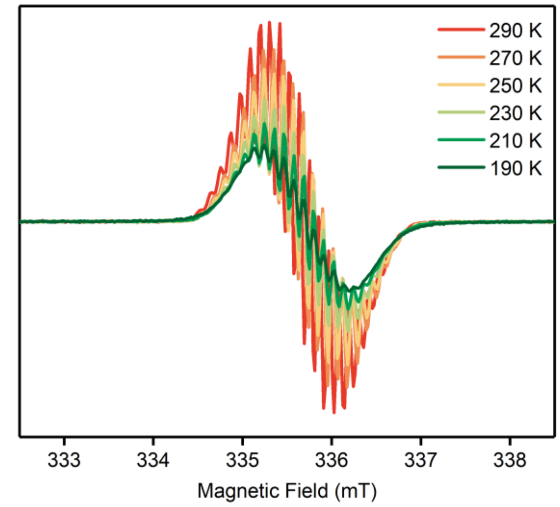

Figure S63. Variable temperature EPR spectra of **1a** in 2-MeTHF solution between 190 K and 290 K (c = 5.0 mM). Microwave freq. 9.38 GHz, powder = 2.0 mW, amp. 0.1 mT.

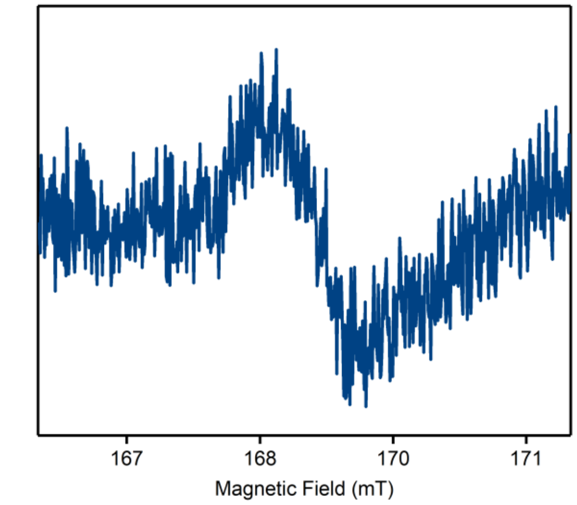

Figure S64. Half-field EPR signal of **1a** in the solid state at 290 K. Microwave freq. 9.38 GHz, powder = 20.0 mW, amp. 0.8 mT.

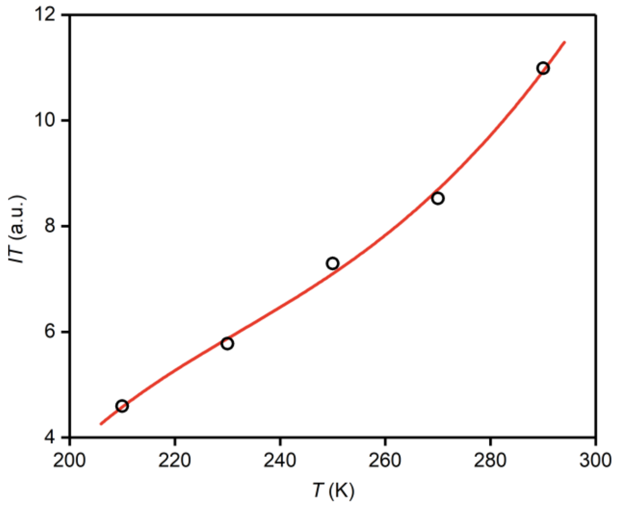

Figure S65. The temperature dependence of the double integral EPR intensity (I) of **1a** in 2-MeTHF solution. Circles (○) represent the experimental results and the red line corresponds to the fit with the Bleaney-Bowers equation. Fitting of the temperature-dependent double-integral intensity to the Bleaney-Bowers equation gives a singlet-triplet gap of $2J = -459$ cm^{-1} ($\Delta E_{ST} = -1.31$ kcal mol^{-1}), indicating a thermally excited triplet state.

5. Computational details

All calculations were performed with the Gaussian(R) 09 program optimizer [11]. The theoretical approach is based on the density functional theory (DFT) framework [12,13]. The geometry optimizations of selected N-heterocyclic carbenes (NHCs) were carried out using the B3LYP functional in combination with the 6-311G(d) and def2-TZVP basis sets. The geometry optimizations of [**1a**$^{2+}$][I^-]$_2$, [**1a**$^{\bullet+}$][I^-], and **1a** were calculated at (U)B3LYP/6-311G(d) level of theory. Frequency analysis was performed to verify the stationary state geometry. In all cases no imaginary frequency was found. Wiberg bond indexes (WBI) of [**1a**$^{2+}$][I^-]$_2$, [**1a**$^{\bullet+}$][I^-], and **1a** were also calculated from the optimized geometry with Löwdin orthogonalization method using Multiwfn program [14].

Nucleus-independent chemical shift (NICS) was employed to study the (anti)aromaticity. The NICS(1)$_{ZZ}$ calculations were performed with the nmr=GIAO key word based on the (U)B3LYP/6-311G(d) level of theory with the help of Multiwfn program. All of the calculated points are set on top the molecules. Time-dependent density functional theory (TD-DFT) was employed to calculate excitation energies. The solvent THF was described in this case by the solvation model based on density (SMD).

The theoretical calculations of NHCs in the literature usually employ methyl as the N-substituent. Under such conditions, the electron pair generally occupies the HOMO orbital, while the empty *p*-orbital is positioned at the LUMO orbital. However, the electronic and steric effects of 2,6-diisopropylphenyl (Dipp) groups should also be considered in practical chemical structures. Therefore, Dipp was selected as the N-substituent for calculations performed at the B3LYP/6-311G(d) level of theory, and presented the relevant molecular orbital distribution of the selected NHCs. To further improve the reliability of the results, additional verification calculations were performed using the def2-TZVP basis set, and the results obtained by the two methods were consistent.

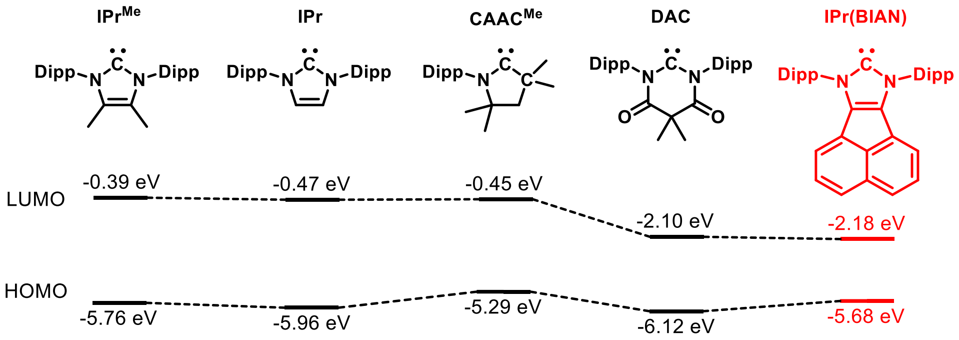

Figure S66. Frontier orbital energies of NHCs calculated at the B3LYP/def2-TZVP level.

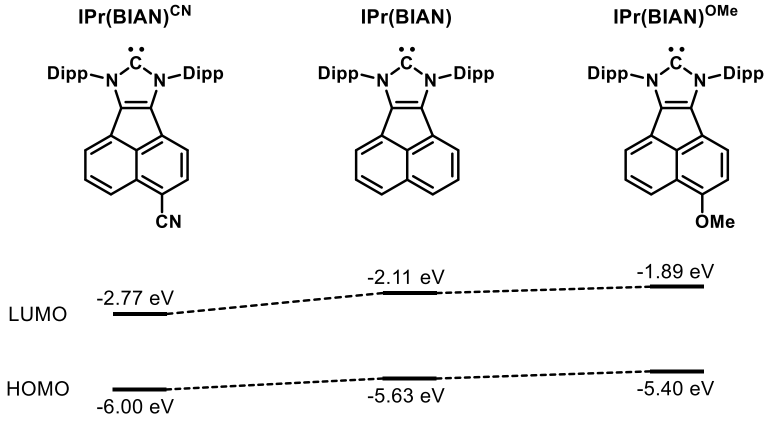

Figure S67. Frontier orbital energies of IPr(BIAN)^{CN}, IPr(BIAN) and IPr(BIAN)^{OMe} calculated at the B3LYP/6-311G(d) level.

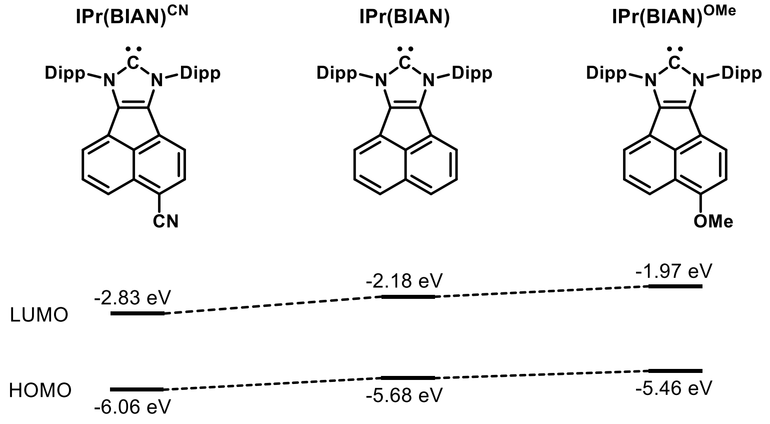

Figure S68. Frontier orbital energies of IPr(BIAN)^{CN}, IPr(BIAN) and IPr(BIAN)^{OMe} calculated at the B3LYP/def2-TZVP level.

	B3LYP/6-311G(d)	B3LYP/def2-TZVP

LUMO+5	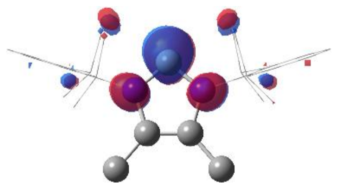 −0.81 eV	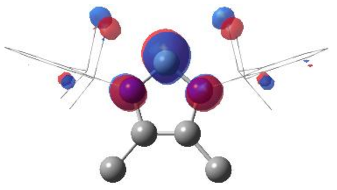 −0.65 eV
LUMO	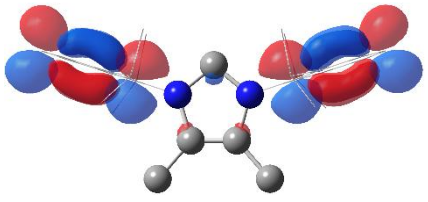 −0.34 eV	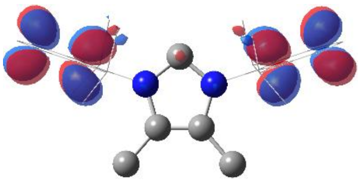 −0.393 eV
HOMO	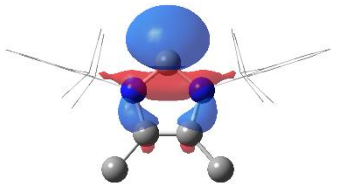 −5.71 eV	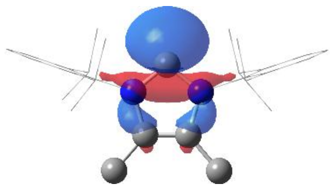 −5.76 eV

Figure S69. Frontier molecular orbitals of IPr^{Me} (isovalue 0.04 a.u.).

	B3LYP/6-311G(d)	B3LYP/def2-TZVP
LUMO+4	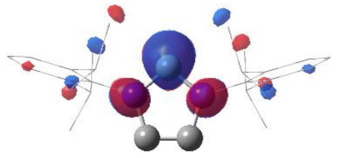 −0.67 eV	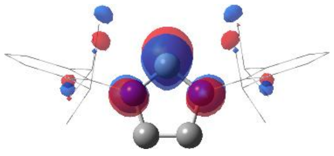 −0.50 eV
LUMO	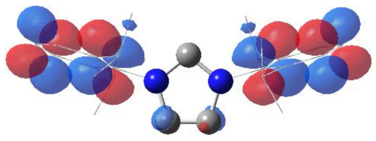 −0.37 eV	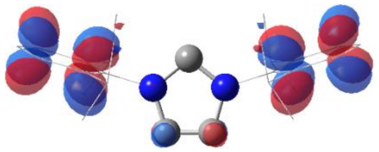 −0.47 eV
HOMO	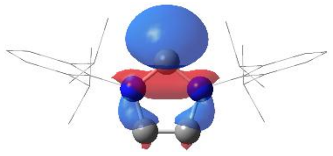 −5.90 eV	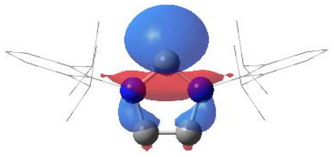 −5.96 eV

Figure S70. Frontier molecular orbitals of IPr (isovalue 0.04 a.u.).

	B3LYP/6-311G(d)	B3LYP/def2-TZVP

LUMO	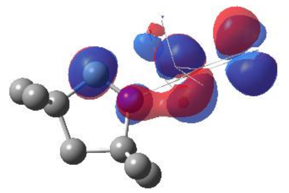<br>−0.37 eV	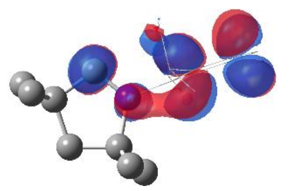<br>−0.45 eV
HOMO	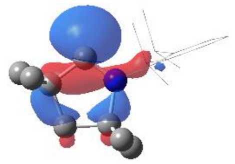<br>−5.26 eV	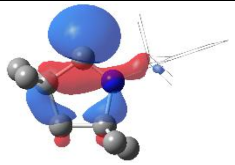<br>−5.29 eV

Figure S71. Frontier molecular orbitals of CAAC^{Me} (isovalue 0.04 a.u.).

	B3LYP/6-311G(d)	B3LYP/def2-TZVP
LUMO	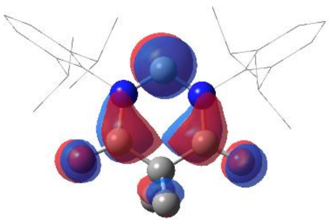<br>−2.01 eV	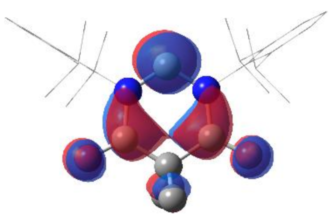<br>−2.10 eV
HOMO	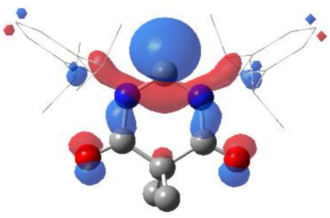<br>−6.07 eV	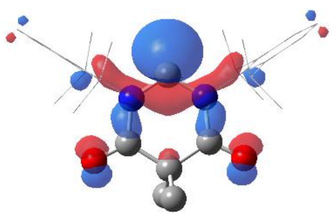<br>−6.12 eV

Figure S72. Frontier molecular orbitals of DAC (isovalue 0.04 a.u.).

	B3LYP/6-311G(d)	B3LYP/def2-TZVP
LUMO+6	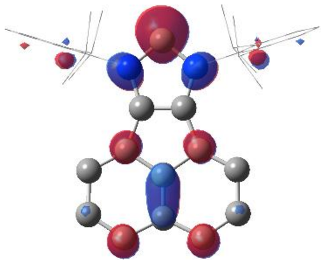 −0.32 eV	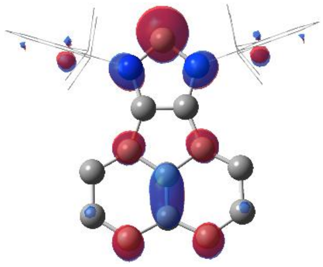 −0.21 eV
LUMO	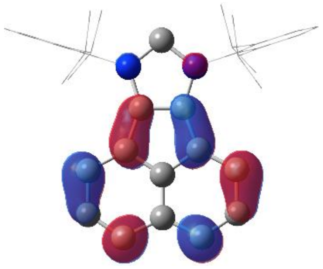 −2.11 eV	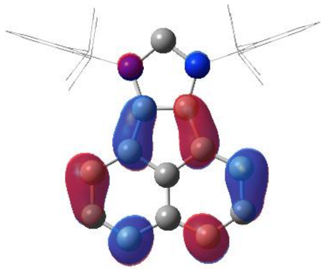 −2.18 eV
HOMO	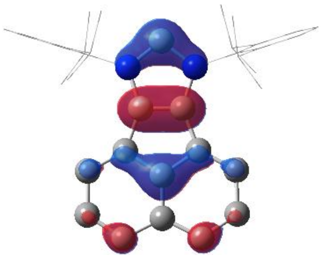 −5.63 eV	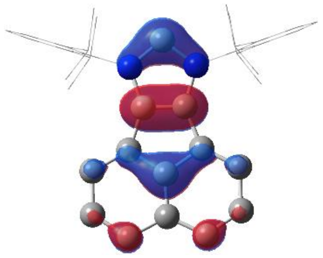 −5.68 eV
HOMO−1	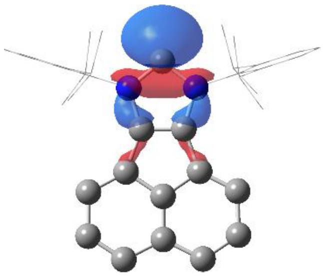 −5.92 eV	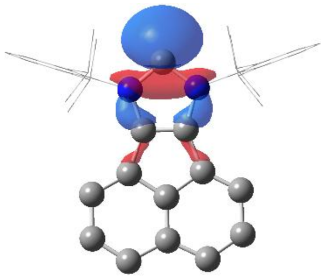 −5.97 eV

Figure S73. Frontier molecular orbitals of IPr(BIAN) (isovalue 0.04 a.u.).

	B3LYP/6-311G(d)	B3LYP/def2-TZVP
LUMO+7	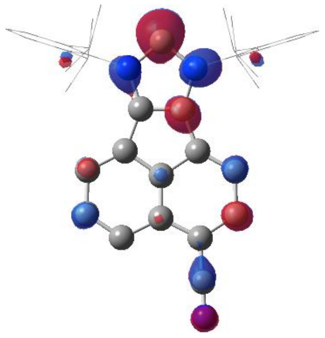 0.39 eV	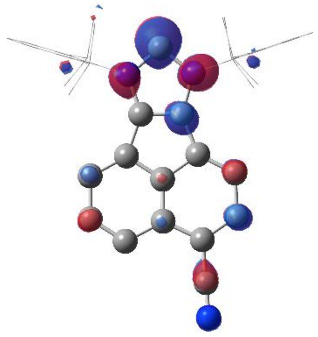 0.29 eV
LUMO	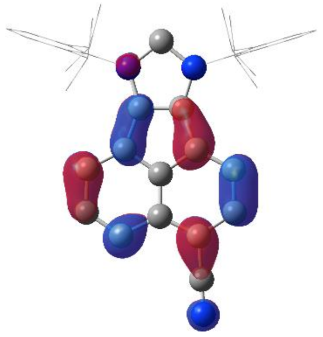 −2.77 eV	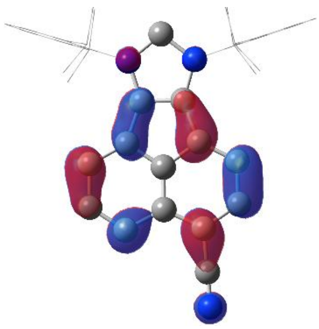 −2.83 eV
HOMO	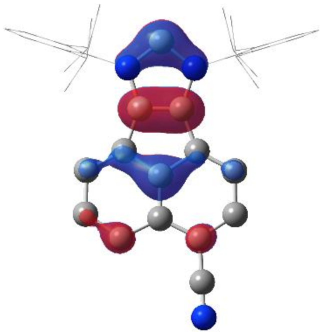 −6.00 eV	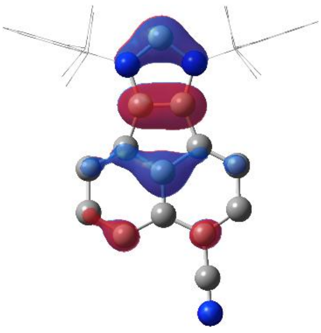 −6.06 eV
HOMO−1	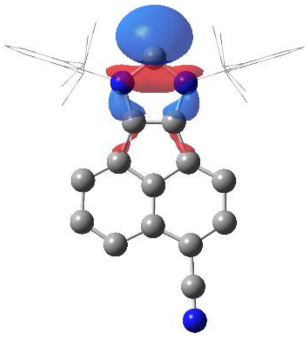 −6.21 eV	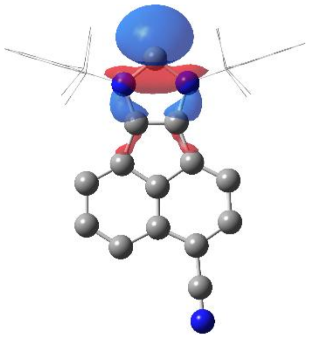 −6.26 eV

Figure S74. Frontier molecular orbitals of IPr(BIAN)^{CN} (isovalue 0.04 a.u.).

	B3LYP/6-311G(d)	B3LYP/def2-TZVP
LUMO+6	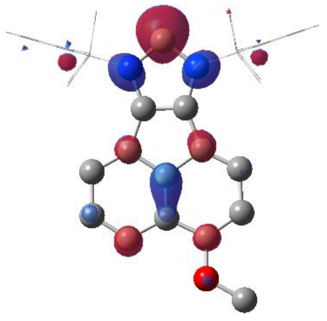 0.47 eV	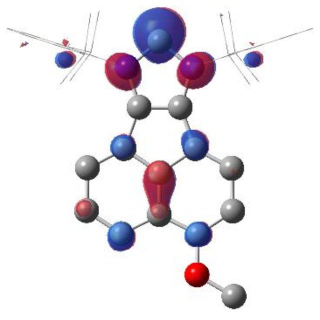 0.38 eV
LUMO	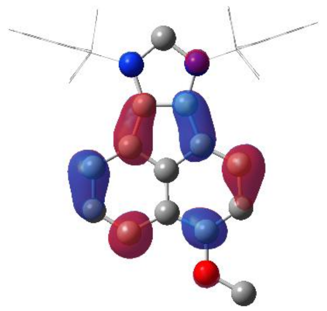 −1.89 eV	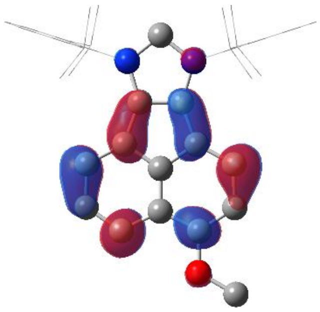 −1.97 eV
HOMO	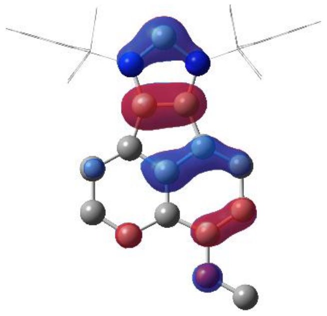 −5.40 eV	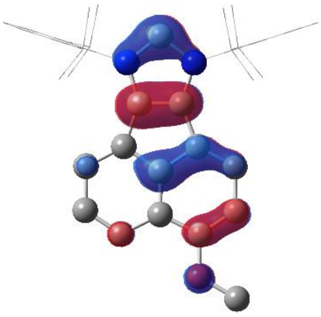 −5.46 eV
HOMO−1	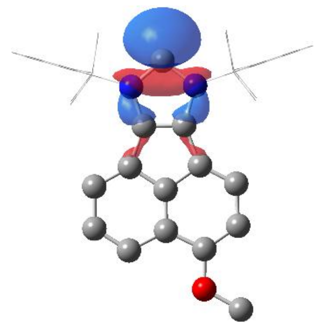 −5.82 eV	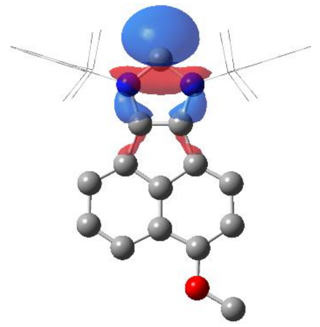 −5.88 eV

Figure S75. Frontier molecular orbitals of IPr(BIAN)^{OMe} (isovalue 0.04 a.u.).

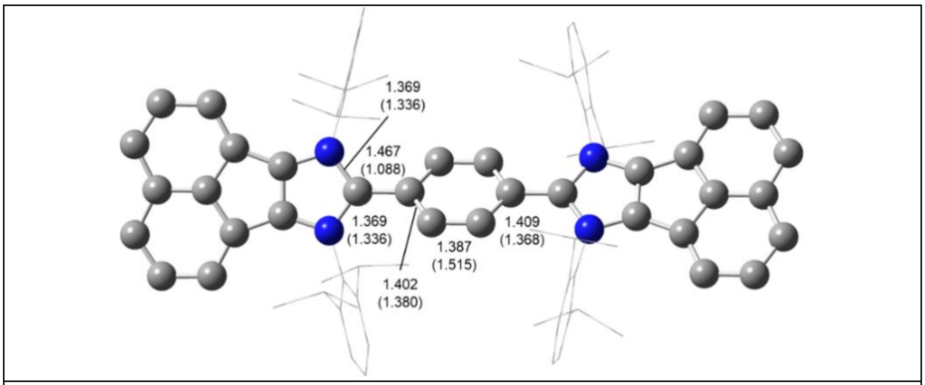

[$\mathbf{1a}^{2+}$][I^-]$_2$

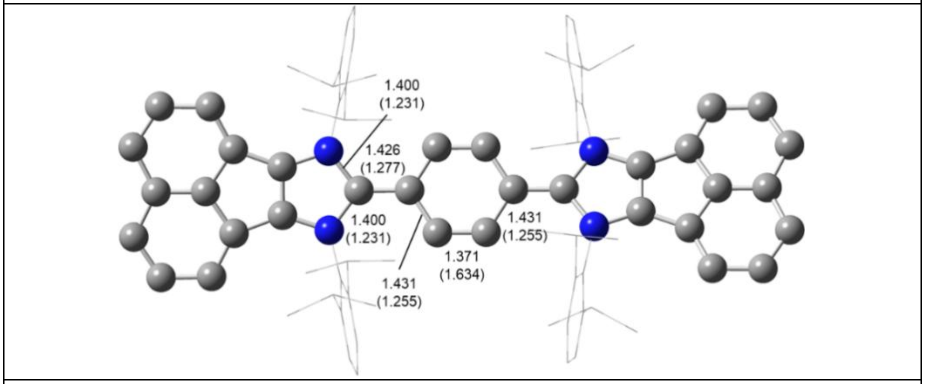

[$\mathbf{1a}^{\bullet+}$][I^-]

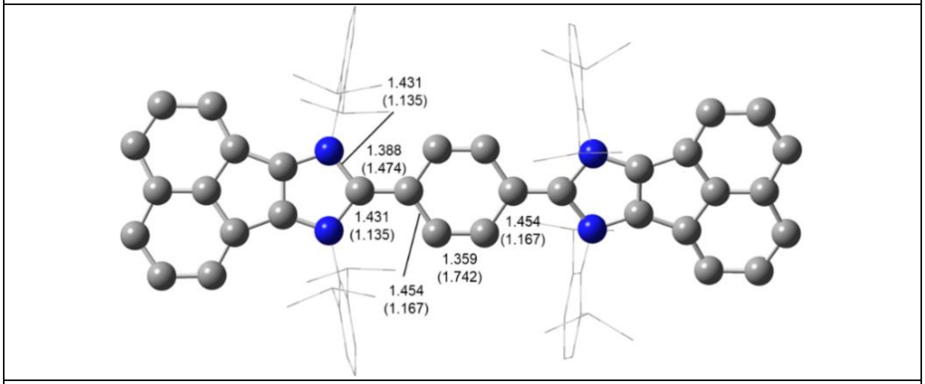

1a

Figure S76. Selected structural parameters and WBI indexes (in parenthesis) of [$\mathbf{1a}^{2+}$][I^-]$_2$, [$\mathbf{1a}^{\bullet+}$][I^-], and **1a**. Hydrogen atoms and counter anion were omitted for clarity.

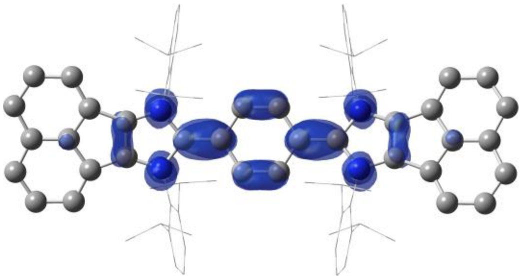

Figure S77. Spin densities at the UB3LYP/6-311G(d) level of theory of [$\mathbf{1a}^{\bullet+}$][I^-] (isovalue 0.0015 a.u.).

Table S8. For compound **1a** electronic energies (E in Hartree) and relative energies (ΔE_{ST} in kcal mol^{-1}) of the different electronic states: closed-shell singlet (CS) and triplet (T).

Method	CS	T		ΔE_{ST}
	E (Hartree)	E (Hartree)	$<S^2>$	(kcal mol^{-1})
(U)B3LYP/6-311G(d)	-3318.761360	-3318.75335	2.0185	-5.03

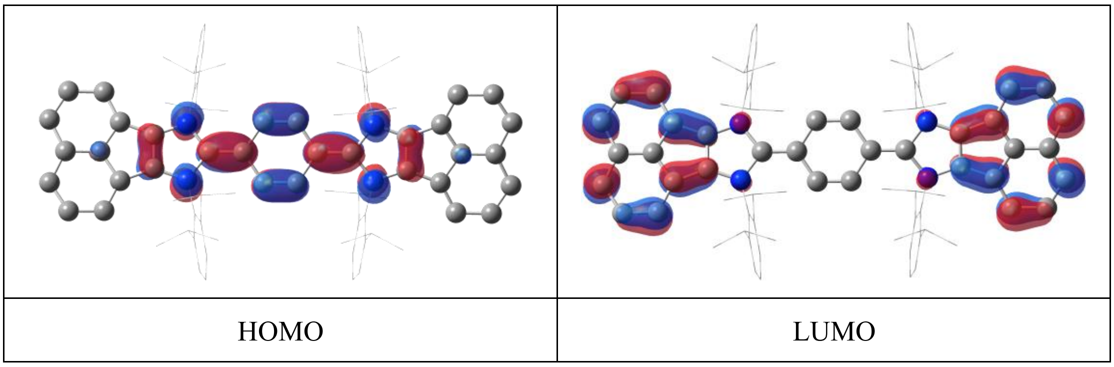

Figure S78. Frontier molecular orbitals of **1a** at the B3LYP/6-311G(d) level of theory for the closed-shell singlet state (isovalue 0.03 a.u.).

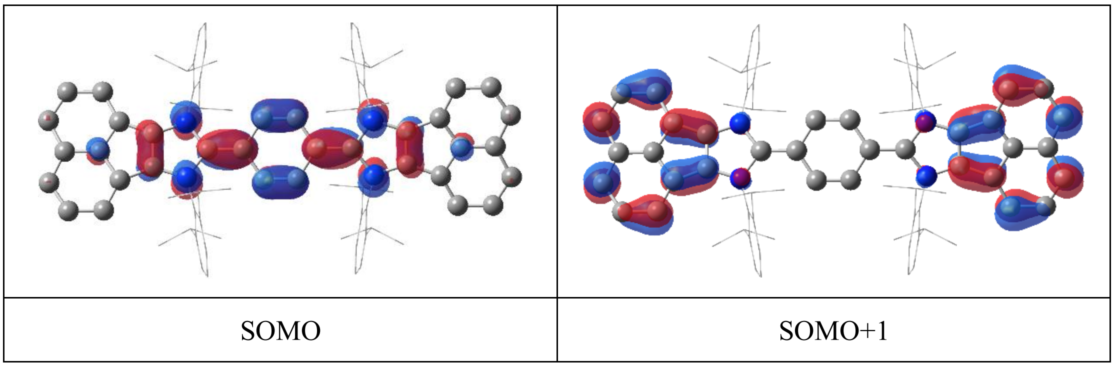

Figure S79. Frontier molecular orbitals of **1a** at the UB3LYP/6-311G(d) level of theory for the triple state (isovalue 0.03 a.u.).

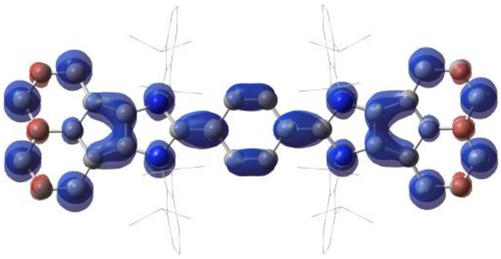

Figure S80. Spin densities of **1a** at the UB3LYP/6-311G(d) level of theory for the triplet state (isovalue 0.0015 a.u.).

Table S9. NICS(1)$_{ZZ}$ data for $[\mathbf{1a}^{2+}][\mathrm{I}^-]_2$, $[\mathbf{1a}^{\bullet+}][\mathrm{I}^-]$, and **1a**.

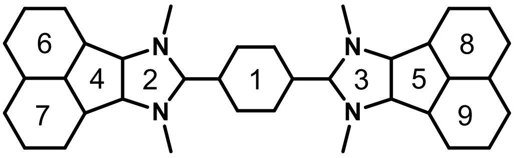

Rings	$[\mathbf{1a}^{2+}][\mathrm{I}^-]_2$	$[\mathbf{1a}^{\bullet+}][\mathrm{I}^-]$	**1a**
1	−23.42	−2.69	10.12
2	−17.79	−10.90	−9.48
3	−16.44	−10.90	−9.48
4	6.45	12.12	25.57
5	6.37	12.12	25.57
6	−22.44	−20.92	−15.25
7	−22.11	−20.63	−15.59
8	−22.24	−20.63	−15.25
9	−22.28	−20.92	−15.59

Table S10. Summary of TD-DFT results for $[\mathbf{1a}^{2+}][\mathrm{I}^-]_2$ in the THF at the ωB97XD/6-311G(d). Wavelength (λ), oscillator strength (f), and main assignment.

λ (nm)	f	Assignment
344.9	2.0180	HOMO→LUMO (82%)

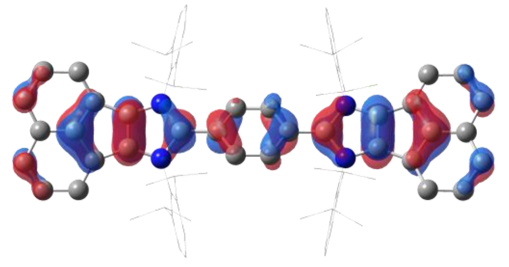	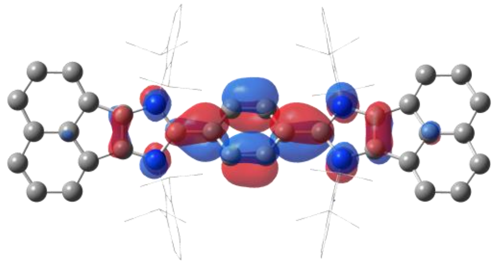
HOMO	LUMO

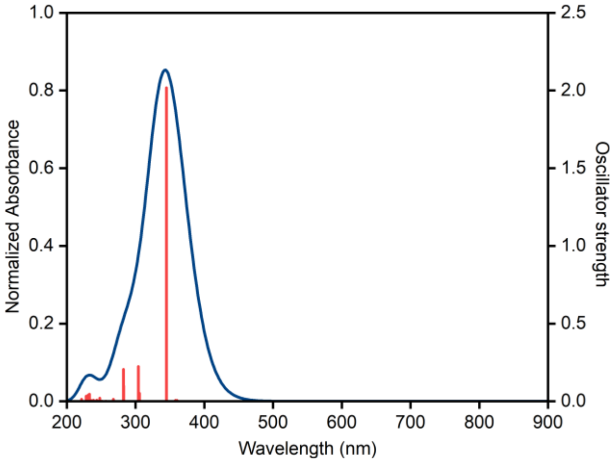

Figure S81. TD-DFT calculated electronic absorption spectra of $[\mathbf{1a}^{2+}][I^{-}]_2$ at the ωB97XD/6-311G(d) level of theory (Solvent = THF).

Table S11. Summary of TD-DFT results for $[\mathbf{1a}^{\bullet+}][I^{-}]$ in the THF at the UCAM-B3LYP/6-311G(d). Wavelength (λ), oscillator strength (f), and main assignment.

λ (nm)	f	Assignment
664.61	0.8371	SOMO(α)→LUMO+2(α) (92%)
475.43	0.9755	HOMO(β)→SUMO (84%)

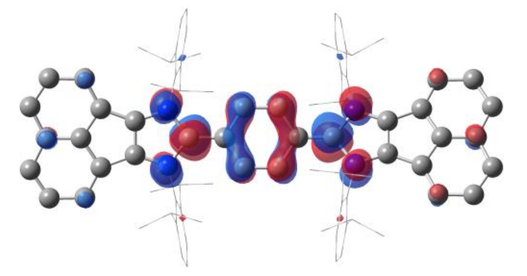	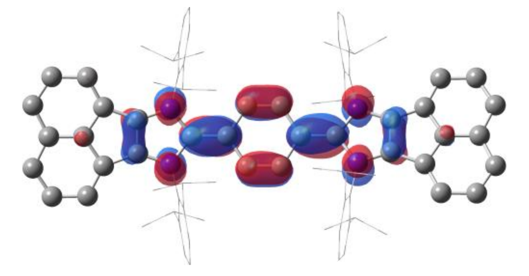
LUMO+2(α)	SUMO
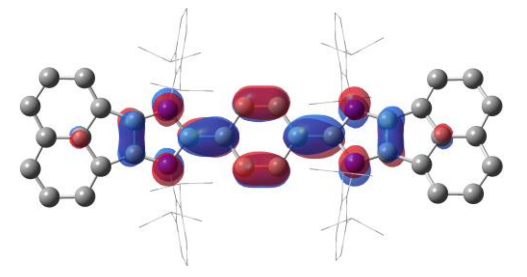	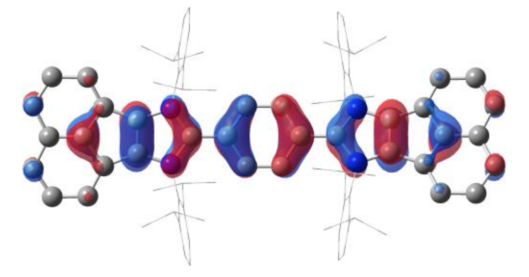
SOMO(α)	HOMO(β)

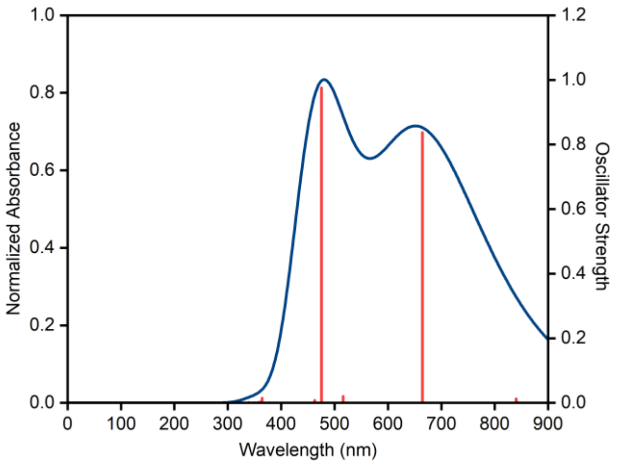

Figure S82. TD-DFT calculated electronic absorption spectra of $[\mathbf{1a}^{\bullet+}][I^-]$ at the UCAM-B3LYP/6-311G(d) level of theory (Solvent = THF).

Table S12. Summary of TD-DFT results for **1a** as a closed-shell singlet state and triplet state in the THF at the (U)ωB97XD/6-311G(d). Wavelength (λ), oscillator strength (f), and main assignment.

Closed-shell singlet state			Triplet state		
λ (nm)	f	Assignment	λ (nm)	f	Assignment
776.55	0.0399	HOMO→LUMO+1 (86%)	806.72	0.0431	HOMO(β)→SUMO (53%)
472.46	2.0504	HOMO→LUMO+2 (68%)	467.52	2.0855	HOMO(α)→LUMO(α) (52%)

Closed-shell singlet state	Triplet state
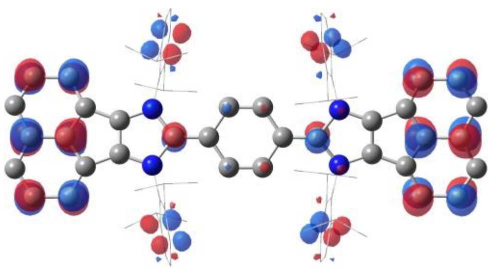	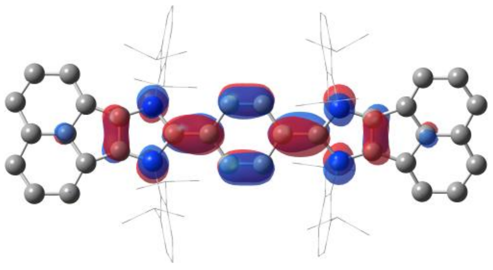
LUMO+2	SUMO

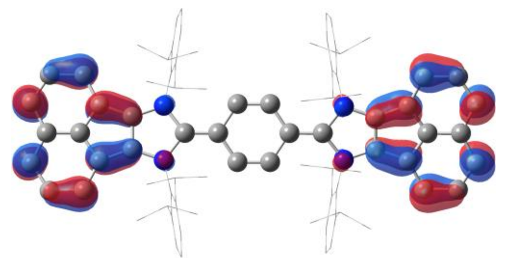	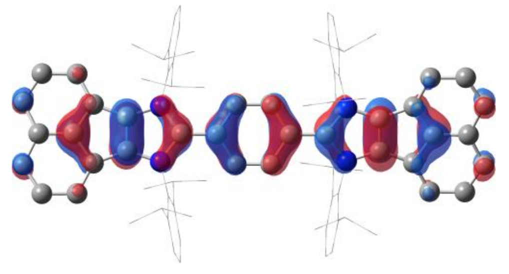
LUMO+1	HOMO(β)
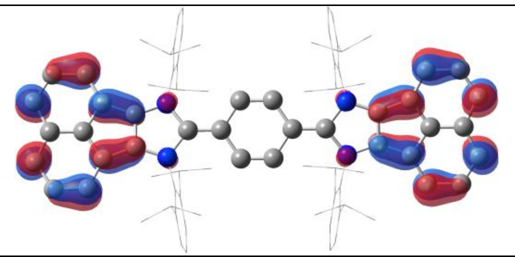	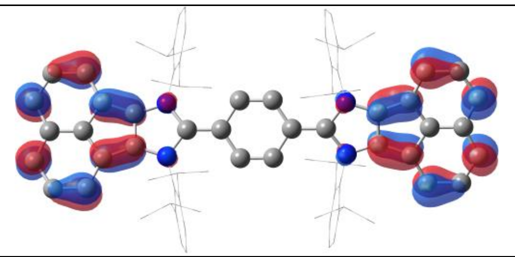
LUMO	LUMO(α)
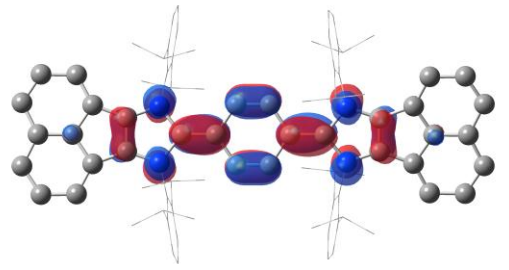	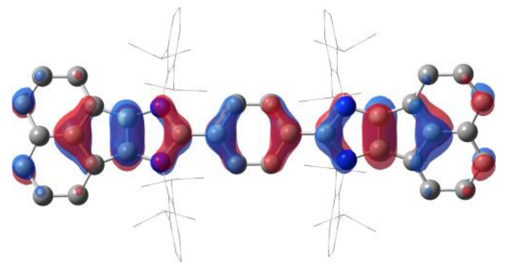
HOMO	HOMO(α)

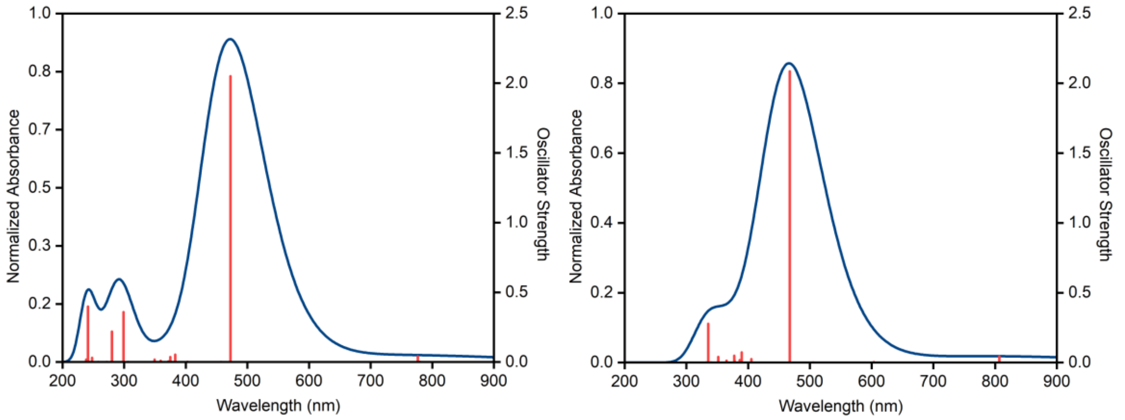

Figure S83. TD-DFT calculated electronic absorption spectra of **1a** for closed-shell singlet state (left) and triplet state (right) at the (U)ωB97XD/6-311G(d) level of theory (Solvent = THF).

6. Photocatalysis

Gas chromatography (GC) analysis was carried out on Agilent 7890B GC system using an HP-5 capillary column (length: 30 m; diam. 0.32 mm; film: 0.25 μm) using N_2 as carrier. GC was equipped with a flame ionization detector (FID). Reduction products were identified by comparing with authentic samples (GC-FID). Quantification of reduction products was performed by GC-FID analysis using internal standards. Photoreduction reactions were performed with 50 W white LED (Perfect Light, PLS-SXE 300$^+$). Anhydrous Et_3N, DMF, DMA and DMSO were purchased and used without distillation. THF and MeCN were dried over Na/benzophenone or CaH_2, distilled prior to use. All manipulations were performed using oven-dried glassware under an argon atmosphere.

6.1 General procedure for the photoreduction

The respective aryl halide or amide (0.05 mmol, 1.0 equiv.), [**1a**$^{2+}$][I^-]$_2$ (0.001 mmol, 0.02 equiv.) and Et_3N (0.40 mmol, 8.0 equiv.) were dissolved in MeCN (3 mL). The reaction mixture was irradiated using a white light. Photoreduction yields were calculated from GC or NMR measurements using internal standards.

6.2 Procedure for carbon-phosphorus coupling

The 4-bromobenzonitrile (0.05 mmol, 1.0 equiv.), triethyl phosphite (0.25 mmol, 5.0 equiv.), [**1a**$^{2+}$][I^-]$_2$ (0.001 mmol, 0.02 equiv.) and Et_3N (0.30 mmol, 6.0 equiv.) were dissolved in MeCN (3 mL). The reaction mixture was irradiated using a white light. Coupling yield was calculated from NMR measurements using internal standard.

6.3 Optimization of photoreduction reaction conditions

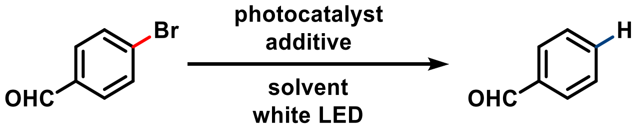

Table S13. Optimization of the types of electron donors[a].

entry	PC (mol%)	additive (equiv.)	solvent	time (h)	yield (%)
1	[**1a**$^{2+}$][I$^-$]$_2$ (5)	DABCO (8)	DMF	12	13
2	[**1a**$^{2+}$][I$^-$]$_2$ (5)	Et_2NH (8)	DMF	12	13
3	[**1a**$^{2+}$][I$^-$]$_2$ (5)	*i*Pr_2NH (8)	DMF	12	< 10
4	[**1a**$^{2+}$][I$^-$]$_2$ (5)	DIPEA (8)	DMF	12	14
5	[**1a**$^{2+}$][I$^-$]$_2$ (5)	DBU (8)	DMF	12	< 10
6	[**1a**$^{2+}$][I$^-$]$_2$ (5)	DMAP (8)	DMF	12	< 10
7	[**1a**$^{2+}$][I$^-$]$_2$ (5)	Pyrrolidine (8)	DMF	12	< 10
8	[**1a**$^{2+}$][I$^-$]$_2$ (5)	Et_3N (8)	DMF	12	**27**

[a]Reactions were carried out using the 4-bromobenzaldehyde (0.05 mmol) in MeCN (3 mL). Yields were calculated from GC measurements using internal standards. PC = photocatalyst.

Table S14. Optimization of reaction solvents[a].

entry	PC (mol%)	additive (equiv.)	solvent	time (h)	yield (%)
1	[**1a**$^{2+}$][I$^-$]$_2$ (5)	Et_3N (8)	DMA	12	< 10
2	[**1a**$^{2+}$][I$^-$]$_2$ (5)	Et_3N (8)	DMSO	12	24
3	[**1a**$^{2+}$][I$^-$]$_2$ (5)	Et_3N (8)	THF	12	20
4	[**1a**$^{2+}$][I$^-$]$_2$ (5)	Et_3N (8)	MeCN	12	**41**
5	[**1a**$^{2+}$][I$^-$]$_2$ (5)	Et_3N (8)	DMF : H_2O = 5 : 1	12	14
6	[**1a**$^{2+}$][I$^-$]$_2$ (5)	Et_3N (8)	DMF : H_2O = 10 : 1	12	15
7	[**1a**$^{2+}$][I$^-$]$_2$ (5)	Et_3N (8)	DMF : H_2O = 20 : 1	12	20
8	[**1a**$^{2+}$][I$^-$]$_2$ (5)	Et_3N (8)	MeCN : H_2O = 5 : 1	12	< 10
9	[**1a**$^{2+}$][I$^-$]$_2$ (5)	Et_3N (8)	MeCN : H_2O = 10 : 1	12	17
10	[**1a**$^{2+}$][I$^-$]$_2$ (5)	Et_3N (8)	MeCN : H_2O = 20 : 1	12	35

[a]Reactions were carried out using the 4-bromobenzaldenhyde (0.05 mmol) in MeCN (3 mL). Yields were calculated from GC measurements using internal standards. PC = photocatalyst.

Table S15. Optimization of catalyst equivalent[a].

entry	PC (mol%)	additive (equiv.)	solvent	time (h)	yield (%)
1	[**1a**$^{2+}$][I$^-$]$_2$ (0.1)	Et_3N (8)	MeCN	12	< 10
2	[**1a**$^{2+}$][I$^-$]$_2$ (0.5)	Et_3N (8)	MeCN	12	13
3	[**1a**$^{2+}$][I$^-$]$_2$ (1)	Et_3N (8)	MeCN	12	33
4	[**1a**$^{2+}$][I$^-$]$_2$ (2)	Et_3N (8)	MeCN	12	**54**
5	[**1a**$^{2+}$][I$^-$]$_2$ (5)	Et_3N (8)	MeCN	12	19
6	[**1a**$^{2+}$][I$^-$]$_2$ (10)	Et_3N (8)	MeCN	12	< 10

[a]Reactions were carried out using the 4-bromobenzaldenhyde (0.05 mmol) in MeCN (3 mL). Yields were calculated from GC measurements using internal standards. PC = photocatalyst.

Table S16. Optimization of electron donor equivalents[a].

entry	PC (mol%)	additive (equiv.)	solvent	time (h)	yield (%)
1	[**1a**$^{2+}$][I$^-$]$_2$ (2)	Et_3N (2)	MeCN	12	36
2	[**1a**$^{2+}$][I$^-$]$_2$ (2)	Et_3N (4)	MeCN	12	47
3	[**1a**$^{2+}$][I$^-$]$_2$ (2)	Et_3N (6)	MeCN	12	51
4	[**1a**$^{2+}$][I$^-$]$_2$ (2)	Et_3N (8)	MeCN	12	**54**

[a]Reactions were carried out using the 4-bromobenzaldenhyde (0.05 mmol) in MeCN (3 mL). Yields were calculated from GC measurements using internal standards. PC = photocatalyst.

Table S17. Optimization of catalyst counter anions[a].

entry	PC (mol%)	additive (equiv.)	solvent	time (h)	yield (%)
1	[**1a**$^{2+}$][I$^-$]$_2$ (2)	Et_3N (8)	MeCN	12	**54**
2	[**1a**$^{2+}$][BF_4^-]$_2$ (2)	Et_3N (8)	MeCN	12	43
3	[**1a**$^{2+}$][PF_6^-]$_2$ (2)	Et_3N (8)	MeCN	12	56

[a]Reactions were carried out using the 4-bromobenzaldenhyde (0.05 mmol) in MeCN (3 mL). Yields were calculated from GC measurements using internal standards. PC = photocatalyst.

6.4 Calculation of the excited-state redox potential

The excited-state redox potential of the photocatalyst was calculated according to a literature report [15,16]. For [**1a**$^{\bullet+}$][I$^-$], $E_{0,0}$ was approximately estimated from the position of the long wavelength tail of the absorption spectrum. The position of the long wavelength tail was found at 829 nm.

$$E_{0,0} = 1240/\lambda \text{ eV} = 1.50 \text{ eV}$$

The ground state potential of [**1a**$^{\bullet+}$][I$^-$] is $E_{1/2}$ = −1.37 V *vs.* Fc/Fc$^+$. The excited-state oxidation potential E(PC^{2+}/PC$^{\bullet+*}$) was calculated as

$$E(\text{PC}^{2+}/\text{PC}^{\bullet+*}) = E_{1/2} - E_{0,0}/\text{e} = -2.87 \text{ V } vs. \text{ Fc/Fc}^+$$

Since the radical cation shows a weak, broad and featureless emission spectrum, it is impossible to determine its $E_{0,0}$ from the crossing point of the absorption and emission spectra.

6.5 Mechanistic experiments

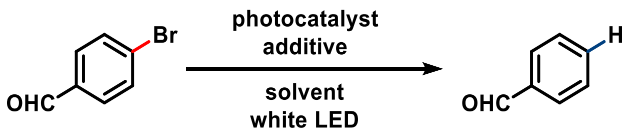

Table S18. Blank experiments[a].

entry	PC (mol%)	additive (equiv.)	solvent	time (h)	yield (%)
1[b]	no PC	Et_3N (8)	MeCN	12	< 10
2[b]	[**1a**$^{2+}$][I$^-$]$_2$ (2)	no Et_3N	MeCN	12	< 10
3	[**1a**$^{2+}$][I$^-$]$_2$ (2)	Et_3N (8), in dark	MeCN	12	0
4[c]	[**1a**$^{\bullet+}$][I$^-$] (2)	Et_3N (8), in dark	MeCN	12	0
5	[**1a**$^{\bullet+}$][I$^-$] (2)	Et_3N (8)	MeCN	12	21

[a]Reactions were carried out using the 4-bromobenzaldenhyde (0.05 mmol) in MeCN (3 mL). Yields were calculated from GC measurements using internal standards. PC = photocatalyst. [b]In the absence of PC or Et_3N, irradiated continuously. [c]Using [**1a**$^{\bullet+}$][I$^-$] instead of [**1a**$^{2+}$][I$^-$]$_2$ and kept under dark.

Table S19. Control experiments of [**1a**$^{2+}$][I^{-}]$_2$ and classical metal-based photocatalysts[a].

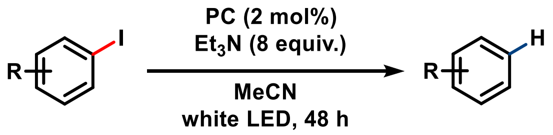

entry	PC	yield (%)		
		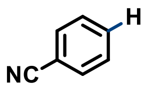	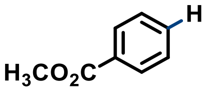	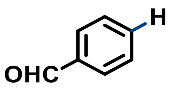
1	[**1a**$^{2+}$][I^{-}]$_2$	**67**	**70**	**54**[b]
2	Ru(bpy)$_3$Cl$_2$	48	61	< 10[b]
3	*fac*-Ir(ppy)$_3$	15	49	< 10[b]

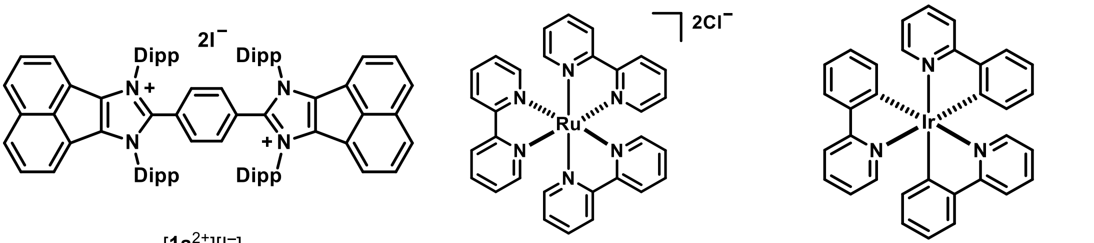

[**1a**$^{2+}$][I^{-}]$_2$ Ru(bpy)$_3$Cl$_2$ *fac*-Ir(ppy)$_3$

[a]Reactions were conducted using the respective aryl halides (0.05 mmol) in MeCN (3 mL). Yields were calculated from GC measurement using internal standards. PC = photocatalyst. [b]4-bromobenzaldehyde was used as substrate. Reaction time is 12 h.

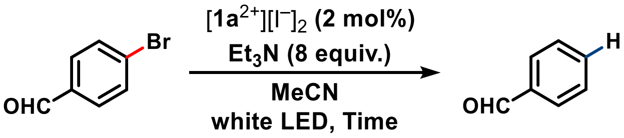

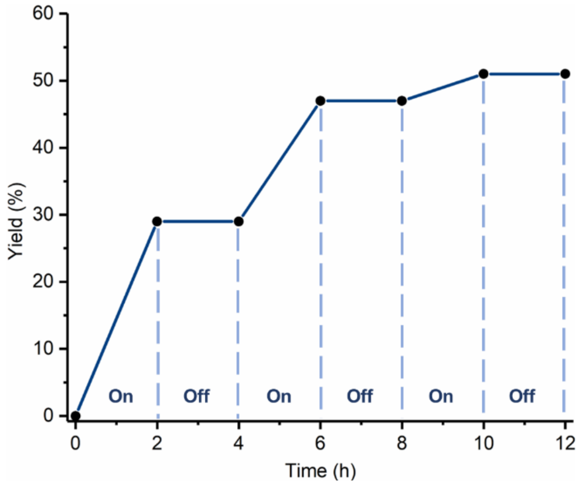

Figure S84. Light on/off experiments.

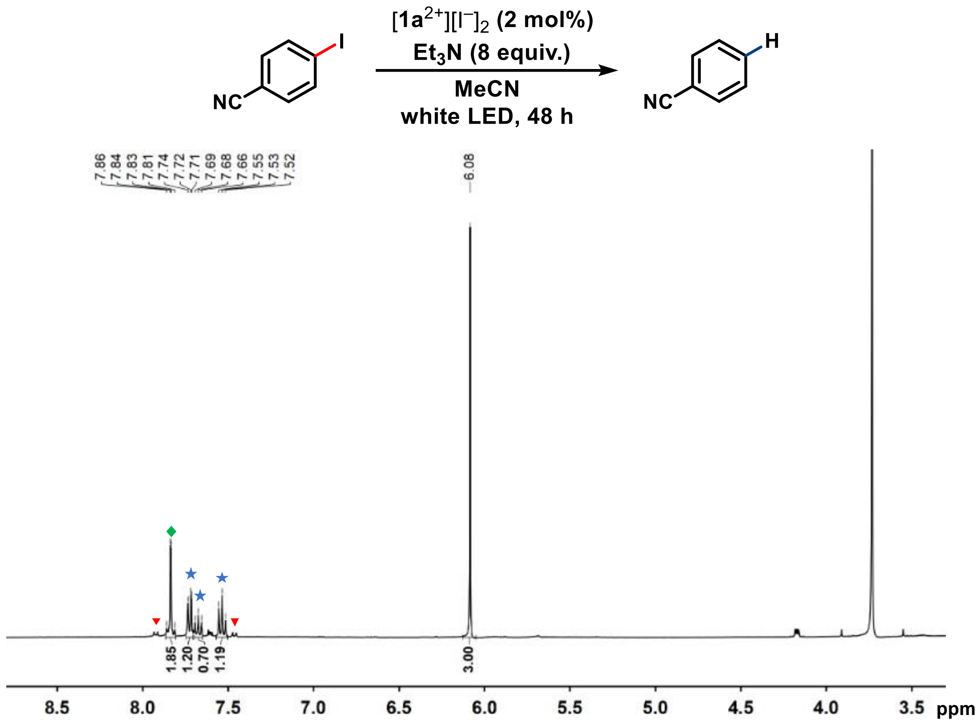

Figure S85. Crude ^{1}H NMR spectrum of dehalogenation of 4-iodobenzonitrile (400 MHz, CD_3CN; ★: target product; ▼: starting materials; ♦: main by-product is 4,4'-biphenyldicarbonitrile).

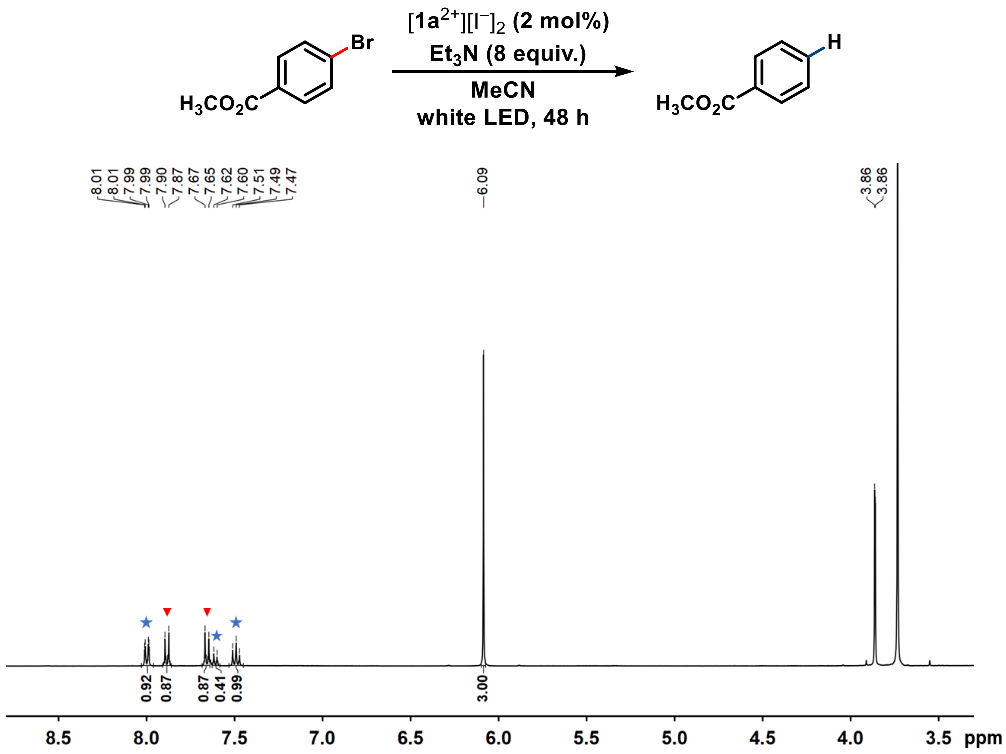

Figure S86. Crude ^{1}H NMR spectrum of dehalogenation of methyl 4-iodobenzoate (400 MHz, $CDCl_3$).

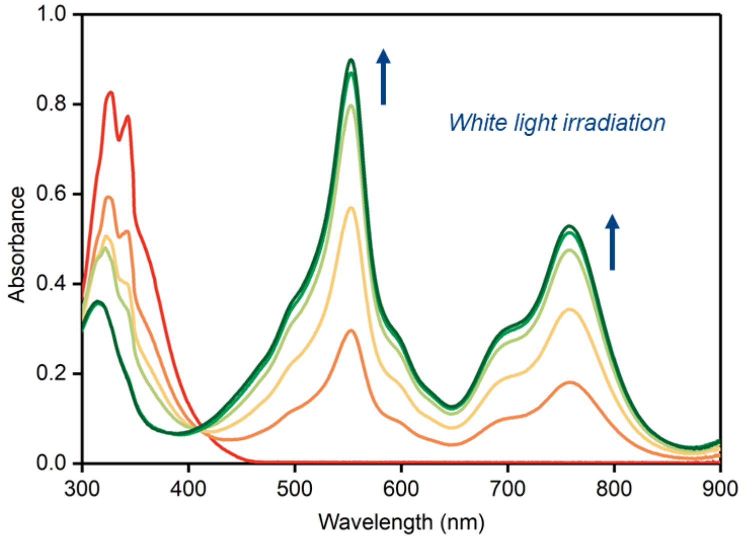

Figure S87. In the presence of Et_3N, [**1a**$^{2+}$][I^-]$_2$ is photoexcited (white light) to form [**1a**$^{\bullet+}$][I^-] (in MeCN solution at room temperature).

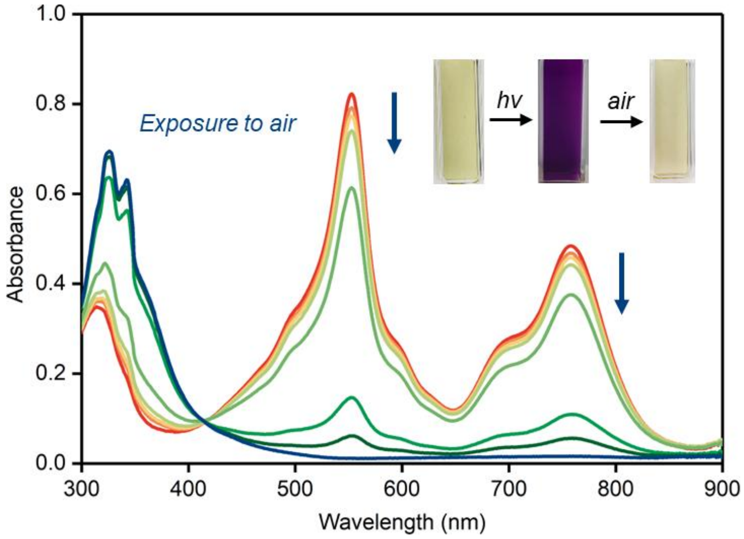

Figure S88. When the irradiated mixture was exposed to air, [**1a**$^{2+}$][I^-]$_2$ is re-formed (in MeCN solution at room temperature).

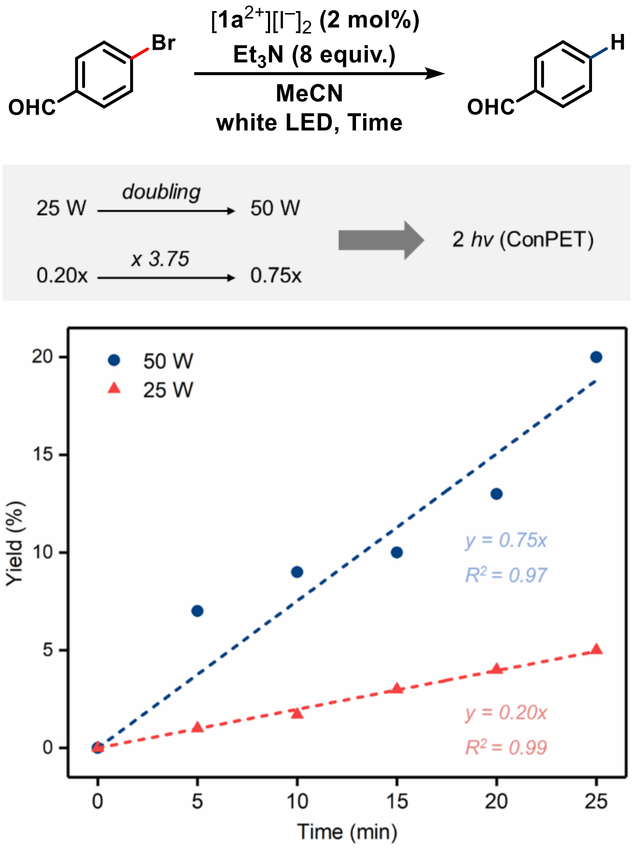

Figure S89. Kinetic analysis by variation of the irradiation intensity.

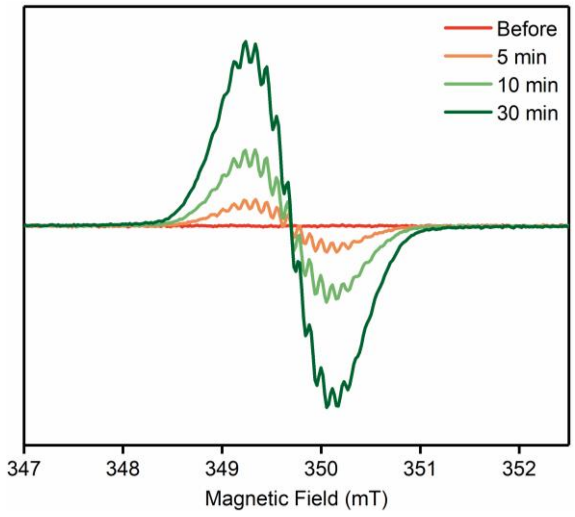

Figure S90. In the presence of Et_3N, [**1a**$^{2+}$][I$^-$]$_2$ is photoexcited (white light) to form [**1a**$^{\bullet+}$][I$^-$] at room temperature.

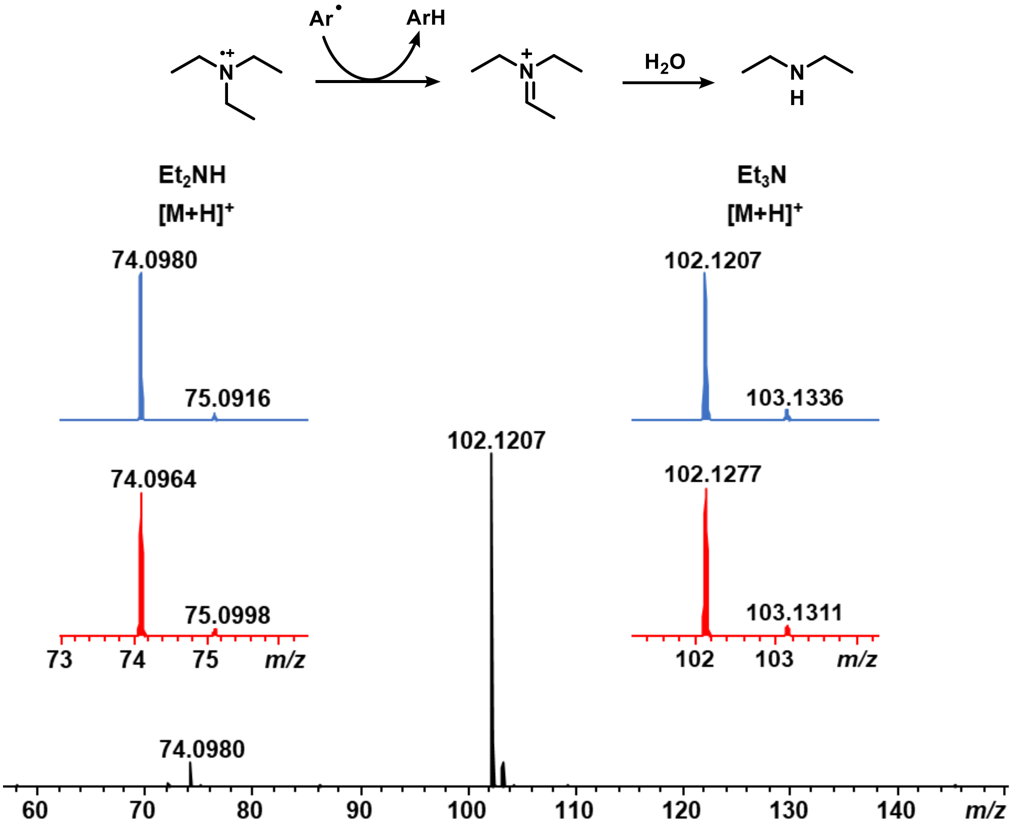

Figure S91. The HRMS spectrum of reaction mixture, inset experimental (blue) and calculated (red) isotope peaks. The diethylamine in the reaction mixture in the result of hydrogen abstraction from the triethylamine radical cation by aryl radicals.

6.6 Direct evidence of the ConPET process in NMR investigation

To a dry NMR tube, 3 mg of [**1a**$^{2+}$][I$^-$]$_2$ added. Then, 0.5 mL of CD_3CN and 16.6 μL of Et_3N were subsequently added. All manipulations were performed under an argon atmosphere using a glovebox.

(1) The ^{1}H NMR spectrum was recorded.

(2) The ^{1}H NMR spectrum was recorded after the solution was irradiated with white light for 2 min.

(3) Then 0.03 mmol of 4-bromobenzaldehyde was added. After the solution was kept in dark for 1 hour, the ^{1}H NMR spectrum was recorded.

(4) After the solution was irradiated with white light for 45 min, the ^{1}H NMR spectrum was recorded.

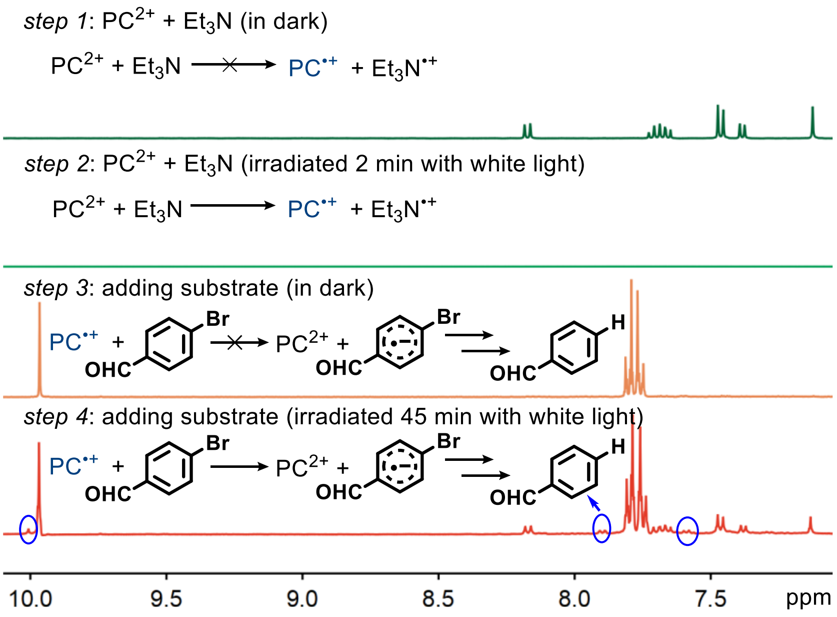

Figure S92. ^{1}H NMR spectra evidence supporting the proposed ConPET process.

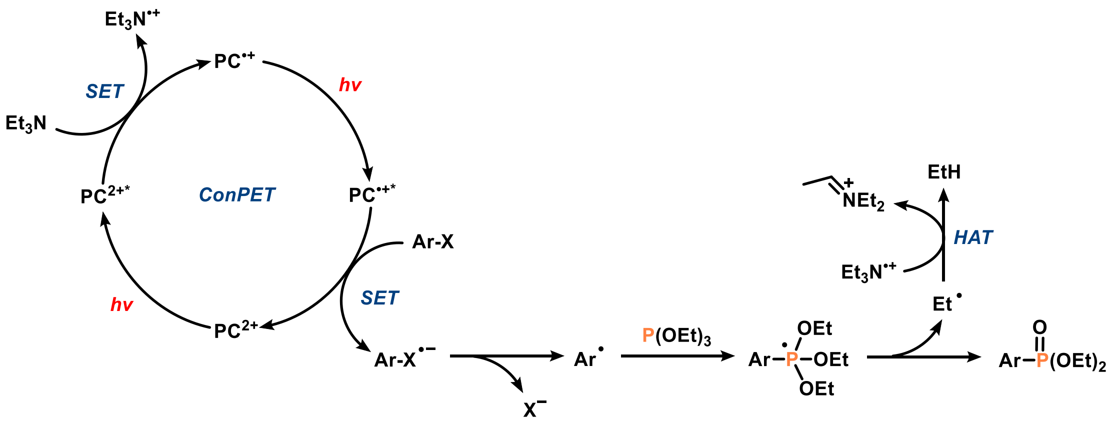

Figure S93. Plausible mechanism for C–P bond construction through the ConPET process.

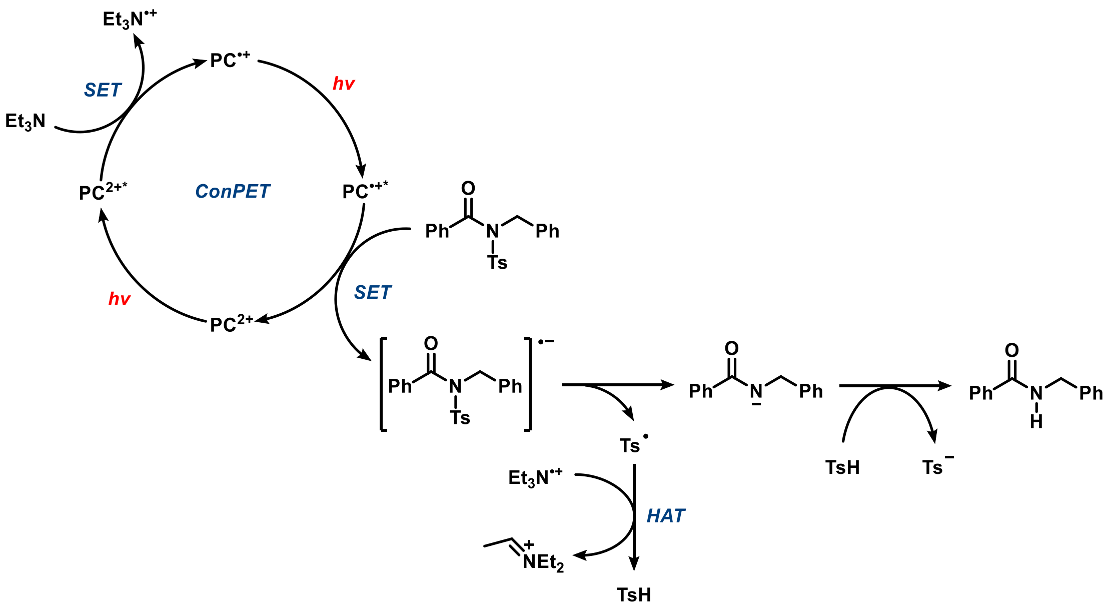

Figure S94. Plausible photoreduction mechanism of *N*-Tosyl-benzamide through the ConPET process.

6.7 Characterization of photocatalytic products

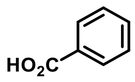	^{1}H NMR (400 MHz, $CDCl_3$): δ = 8.13 (d, J = 7.1 Hz, 2H), 7.63 (t, J = 7.4 Hz, 1H), 7.49 (t, J = 7.7 Hz, 2H) ppm. MS (EI): m/z = 122 $[M]^+$. Spectroscopic data are consistent with literature reports [17].
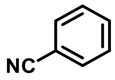	^{1}H NMR (400 MHz, $CDCl_3$): δ = 7.66 (d, J = 7.0 Hz, 2H), 7.61 (t, J = 7.6 Hz, 1H), 7.48 (t, J = 7.7 Hz, 2H) ppm. MS (EI): m/z = 103 $[M]^+$. Spectroscopic data are consistent with literature reports [18].
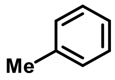	^{1}H NMR (400 MHz, $CDCl_3$): δ = 7.29 (d, J = 7.5 Hz, 2H), 7.22-7.15 (m, 3H), 2.38 (s, 3H) ppm. MS (EI): m/z = 91 $[M{-}H]^+$. Spectroscopic data are consistent with literature reports [18].
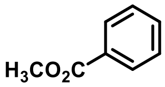	^{1}H NMR (400 MHz, $CDCl_3$): δ = 8.04 (d, J = 7.0 Hz, 2H), 7.56 (t, J = 7.4 Hz, 1H), 7.44 (t, J = 7.7 Hz, 2H), 3.92 (s, 3H) ppm. MS (EI): m/z = 136 $[M]^+$. Spectroscopic data are consistent with literature reports [19].
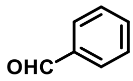	^{1}H NMR (400 MHz, $CDCl_3$): δ = 10.03 (s, 1H), 7.89 (d, J = 6.9 Hz, 2H), 7.64 (t, J = 7.4 Hz, 1H), 7.54 (t, J = 7.5 Hz, 2H) ppm. MS (EI): m/z = 106 $[M]^+$. Spectroscopic data are consistent with literature reports [18].

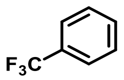	^{1}H NMR (400 MHz, $CDCl_3$): δ = 7.63 (d, J = 7.6 Hz, 2H), 7.56 (t, J = 7.1 Hz, 1H), 7.49 (t, J = 7.5 Hz, 2H) ppm. MS (EI): m/z = 146 $[M]^+$. Spectroscopic data are consistent with literature reports [20].
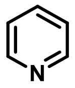	^{1}H NMR (400 MHz, $CDCl_3$): δ = 8.62 (d, J = 4.2 Hz, 2H), 7.72 (t, J = 7.6 Hz, 1H), 7.38-7.28 (m, 2H) ppm. MS (EI): m/z = 79 $[M]^+$. Spectroscopic data are consistent with literature reports [18].
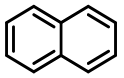	^{1}H NMR (400 MHz, $CDCl_3$): δ = 7.89-7.82 (m, 4H), 7.53-7.44 (m, 4H) ppm. MS (EI): m/z = 128 $[M]^+$. Spectroscopic data are consistent with literature reports [18].
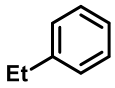	^{1}H NMR (400 MHz, $CDCl_3$): δ = 7.33-7.27 (m, 2H), 7.24-7.15 (m, 3H), 2.67 (q, J = 7.6 Hz, 2H), 1.25 (t, J = 7.6 Hz, 3H) ppm. MS (EI): m/z = 106 $[M]^+$. Spectroscopic data are consistent with literature reports [21].
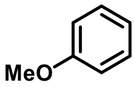	^{1}H NMR (400 MHz, $CDCl_3$): δ = 7.34-7.27 (m, 2H), 6.98-6.89 (m, 3H), 3.82 (s, 3H) ppm. MS (EI): m/z = 108 $[M]^+$. Spectroscopic data are consistent with literature reports [18].
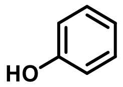	^{1}H NMR (400 MHz, $CDCl_3$): δ = 7.32-7.24 (m, 2H), 6.97 (t, J = 7.4 Hz, 1H), 6.91-6.83 (m, 2H), 4.88 (s, 1H) ppm. MS (EI): m/z = 94 $[M]^+$. Spectroscopic data are consistent with literature reports [18].
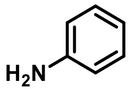	^{1}H NMR (400 MHz, $CDCl_3$): δ = 7.21-7.12 (m, 2H), 6.77 (t, J = 7.4 Hz, 1H), 6.70 (d, J = 7.5 Hz, 2H), 3.65 (s, 2H) ppm. MS (EI): m/z = 93 $[M]^+$. Spectroscopic data are consistent with literature reports [18].
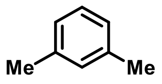	^{1}H NMR (400 MHz, $CDCl_3$): δ = 7.16 (t, J = 7.5 Hz, 1H), 7.04-6.96 (m, 3H), 2.33 (d, J = 0.8 Hz, 6H) ppm. MS (EI): m/z = 106 $[M]^+$. Spectroscopic data are consistent with literature reports [22].
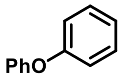	^{1}H NMR (400 MHz, $CDCl_3$): δ = 7.38-7.30 (m, 4H), 7.11 (t, J = 7.4 Hz, 2H), 7.02 (d, J = 8.6 Hz, 4H) ppm. MS (EI): m/z = 170 $[M]^+$. Spectroscopic data are consistent with literature reports [22].
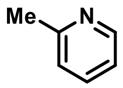	^{1}H NMR (400 MHz, $CDCl_3$): δ = 8.49 (d, J = 4.3 Hz, 1H), 7.59-7.52 (m, 1H), 7.14 (d, J = 7.8 Hz, 1H), 7.10-7.04 (m, 1H), 2.55 (s, 3H) ppm. MS

	(EI): m/z = 93 $[M]^+$. Spectroscopic data are consistent with literature reports [23].
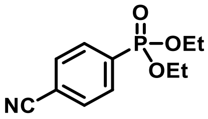	^1H NMR (400 MHz, $CDCl_3$): δ = 7.98-7.87 (m, 2H), 7.79-7.70 (m, 2H), 4.24-4.05 (m, 4H), 1.33 (t, J = 7.1 Hz, 6H) ppm. MS (EI): m/z = 239 $[M]^+$. Spectroscopic data are consistent with literature reports [22].
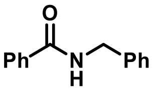	^1H NMR (400 MHz, $CDCl_3$): δ = 7.86-7.74 (m, 2H), 7.50 (t, J = 7.3 Hz, 1H), 7.43 (t, J = 7.5 Hz, 2H), 7.36 (d, J = 4.4 Hz, 4H), 7.34-7.27 (m, 1H), 6.45 (s, 1H), 4.65 (d, J = 5.7 Hz, 2H) ppm. MS (EI): m/z = 211 $[M]^+$. Spectroscopic data are consistent with literature reports [24].

7. Cartesian coordinates of the optimized geometries by DFT calculations

Table S20. Cartesians coordinates of calculated IPr^{Me} at the B3LYP/6-311G(d) level of theory.

Atom	X	Y	Z
N	1.06287104	-0.00781795	0.34501215
N	-1.06297399	0.00788964	0.34499196
C	-0.00003998	0.00003442	-0.51831311
C	-0.67940475	0.005124	1.69947802
C	0.67928685	-0.00508702	1.69948433
C	2.43474409	-0.01925235	-0.09682486
C	3.09747801	1.20388055	-0.30684711
C	4.42904696	1.16336335	-0.73145965
C	5.08027916	-0.0453211	-0.93884168
C	4.40605135	-1.24179446	-0.72885506
C	3.07488393	-1.25594266	-0.30333579
C	2.39948087	2.54793625	-0.13653058
C	2.1527963	3.20644342	-1.50615033
C	3.16266811	3.49476541	0.80552439
C	2.34060544	-2.58140632	-0.13912315
C	1.92971612	-3.13444664	-1.5165445
C	3.14403133	-3.62043243	0.66030275
C	-2.43486231	0.01926251	-0.09676545
C	-3.07508383	1.25594866	-0.30317067
C	-4.40630725	1.2417592	-0.72847726
C	-5.08050018	0.04524663	-0.93845505
C	-4.42918016	-1.16340513	-0.73124947
C	-3.09755806	-1.2038761	-0.30676632
C	-2.34073921	2.58139315	-0.13913479
C	-3.14451208	3.62108578	0.65905177
C	-1.92877551	3.13352164	-1.51662105
C	-2.39940499	-2.54788358	-0.13664994
C	-3.16274239	-3.4951883	0.80478842
C	-2.15209114	-3.20581421	-1.5064275
H	4.96164752	2.09179334	-0.90974995
H	6.11401366	-0.05558364	-1.27064588
H	4.92156879	-2.17964715	-0.90581048
H	1.42100244	2.36228166	0.3103139
H	1.60120161	4.14444008	-1.3897836
H	1.5709042	2.54814912	-2.15397135
H	3.09464803	3.43515303	-2.01425836
H	2.59439881	4.41680145	0.96007194
H	4.13812046	3.77923259	0.40103516
H	3.33308918	3.03919332	1.78497518
H	1.42091918	-2.38658877	0.41682548
H	1.36427054	-4.06527393	-1.40780036
H	2.80836099	-3.34620431	-2.13373229
H	1.30545312	-2.41551831	-2.0503326
H	2.5333904	-4.50927322	0.84407311
H	3.46296645	-3.22703145	1.62951233
H	4.0392953	-3.95155039	0.12671873
H	-4.92191883	2.17958362	-0.9052805
H	-6.11428534	0.05548065	-1.27010197
H	-4.96173397	-2.09185459	-0.90955604
H	-1.42146962	2.38674186	0.41757327
H	-2.53353719	4.50958868	0.84335944
H	-3.46492471	3.22814326	1.62796533
H	-4.03888237	3.95266954	0.12424926

H	-1.36341056	4.06442156	-1.40807081	H	-3.09371223	-3.43443883	-2.01499977
H	-2.80697863	3.34485831	-2.13458564	C	-1.65790714	0.0144048	2.82600675
H	-1.30412222	2.4142203	-2.04944371	H	-2.31770075	-0.85809886	2.80126794
H	-1.42111411	-2.36220558	0.31060695	H	-1.13887036	0.0096509	3.7857764
H	-4.1379245	-3.77983889	0.39978163	H	-2.30131121	0.89920209	2.80120658
H	-3.3337385	-3.03992399	1.78428624	C	1.65776975	-0.0143746	2.82603288
H	-2.59428233	-4.4171048	0.9593473	H	2.31776277	0.85797206	2.80112137
H	-1.60042907	-4.1437913	-1.39020939	H	1.13873008	-0.00930621	3.78579948
H	-1.57001692	-2.54718664	-2.15374268	H	2.30096489	-0.89932768	2.8014237

E(B3LYP/6-311G(d)) = -1238.926699 Hartree

Table S21. Cartesians coordinates of calculated IPr^{Me} at the B3LYP/def2-TZVP level of theory.

Atom	X	Y	Z				
				C	3.15502144	-3.52116573	0.77348026
N	1.06083733	-0.00003938	0.34479464	C	-2.42709014	-0.00020163	-0.10406007
N	-1.06102745	-0.00022095	0.34491561	C	-3.0745853	1.2269811	-0.32122135
C	-0.00014529	-0.00020399	-0.51276603	C	-4.39620581	1.199502	-0.76570044
C	-0.67837678	-0.00010846	1.69491321	C	-5.05395669	-0.00019019	-0.98581507
C	0.67832593	0.00001316	1.69483968	C	-4.39605398	-1.19992578	-0.76612417
C	2.42688565	0.00012212	-0.10416966	C	-3.07444111	-1.2274172	-0.32171754
C	3.07406333	1.22737527	-0.32198586	C	-2.36981558	2.56199143	-0.13293505
C	4.39574368	1.20002521	-0.7662702	C	-3.15490478	3.52128842	0.77310943
C	5.05388307	0.00039407	-0.98563818	C	-2.06405652	3.21059955	-1.49240194
C	4.39627866	-1.19938724	-0.76540485	C	-2.36950342	-2.56239817	-0.13399475
C	3.07461074	-1.22700584	-0.32114257	C	-3.15487201	-3.52252799	0.77094037
C	2.36901877	2.56234383	-0.13447626	C	-2.06290297	-3.2099864	-1.49378097
C	2.06493225	3.21118293	-1.49421586	H	4.91562649	2.13162194	-0.95036675
C	3.15303283	3.52149878	0.77265586	H	6.0798306	0.00049539	-1.33285915
C	2.37002438	-2.56210869	-0.13288201	H	4.91660047	-2.13087142	-0.94881446
C	2.06480495	-3.21097511	-1.49236752	H	1.41215184	2.36780291	0.34908059

H	1.51733335	4.14668609	-1.35754166	H	-4.10736134	3.81487354	0.32731996
H	1.46087454	2.54761093	-2.113358	H	-1.51691844	4.14630101	-1.35521978
H	2.98602472	3.43827338	-2.03617339	H	-2.98452087	3.43725473	-2.03559782
H	2.57714795	4.43397307	0.94230837	H	-1.45900255	2.5470323	-2.1105828
H	4.10610704	3.8149525	0.32810052	H	-1.4135447	-2.36807267	0.35146712
H	3.36529226	3.07120774	1.74442735	H	-4.10634255	-3.8171889	0.32374782
H	1.41358044	-2.36771677	0.35158966	H	-3.37033484	-3.07256805	1.74215623
H	1.51786893	-4.14679763	-1.35522173	H	-2.57832751	-4.4343047	0.94208636
H	2.98547231	-3.43746068	-2.03528673	H	-1.51567967	-4.14567025	-1.35703233
H	1.45973834	-2.54765574	-2.1107975	H	-1.45766036	-2.54584238	-2.11115239
H	2.57906233	-4.43337445	0.94429831	H	-2.98306238	-3.4363693	-2.03761884
H	3.36886236	-3.07061008	1.74478287	C	-1.65163477	-0.00007687	2.8220515
H	4.1073394	-3.81517155	0.32765017	H	-2.30180872	-0.87788087	2.79766576
H	-4.91634963	2.1310413	-0.94932717	H	-1.12588682	-0.0009599	3.77607277
H	-6.07985632	-0.00020257	-1.33317902	H	-2.30067088	0.87860579	2.7987036
H	-4.91614459	-2.1314399	-0.95004515	C	1.65167167	0.00041774	2.82189502
H	-1.41356822	2.36747412	0.35188959	H	2.30083684	0.8789905	2.79800945
H	-2.57913587	4.43370613	0.9434226	H	1.12596291	0.00012771	3.77593526
H	-3.36845406	3.07109167	1.74463783	H	2.30173481	-0.87748052	2.79796077

E(B3LYP/def2-TZVP) = -1239.119211 Hartree

Table S22. Cartesians coordinates of calculated IPr at the B3LYP/6-311G(d) level of theory.

Atom	X	Y	Z				
N	-1.06275912	0.0650466	0.51746426	C	-3.20343125	-1.03901613	0.05415441
N	1.06270196	-0.06517526	0.5174584	C	-4.54077664	-0.93641453	-0.34132526
C	-0.00003975	-0.00009943	-0.34745167	C	-5.09389447	0.28822093	-0.69009053
C	0.67446874	-0.04230046	1.85790647	C	-4.31638301	1.4392005	-0.64873839
C	-0.67451466	0.04227899	1.85791287	C	-2.97710292	1.39390187	-0.25208015
C	-2.4387673	0.14048985	0.09509647	C	-2.62104892	-2.40632025	0.39421628
				C	-2.63796024	-3.33825753	-0.83062088

C	-3.33439448	-3.05206464	1.59521108	H	-4.3869837	-3.25821644	1.38033666
C	-2.1337014	2.663243	-0.25213103	H	-3.29847907	-2.40654354	2.47698479
C	-1.670772	2.99786688	-1.68266783	H	-1.23650896	2.46518429	0.3384652
C	-2.84720524	3.85933046	0.39889234	H	-1.03585399	3.88959859	-1.68441396
C	2.43870084	-0.14051604	0.09506951	H	-2.52575719	3.19413381	-2.33737944
C	2.97715741	-1.39386241	-0.25212424	H	-1.09882238	2.16981651	-2.10538424
C	4.31644501	-1.43902335	-0.64881871	H	-2.16111498	4.70756491	0.47842343
C	5.09382802	-0.28796629	-0.69018294	H	-3.20100865	3.61910807	1.40521052
C	4.54059132	0.93661356	-0.34137248	H	-3.70785581	4.19802245	-0.18468363
C	3.20326237	1.0390774	0.05413768	H	4.75900568	-2.38809119	-0.93072293
C	2.13399018	-2.66333873	-0.25208776	H	6.13299773	-0.34611206	-0.99919215
C	2.84760733	-3.85914218	0.39935115	H	5.1544935	1.83035558	-0.38487955
C	1.67141289	-2.9983194	-1.68265014	H	1.23663783	-2.46535448	0.33826653
C	2.62073005	2.40629439	0.39426536	H	2.16167047	-4.70749481	0.47893915
C	3.33376556	3.05186367	1.59554894	H	3.2011366	-3.61859053	1.40568174
C	2.63793183	3.33844423	-0.83041785	H	3.7084615	-4.19779531	-0.1839518
H	1.38226923	-0.08743125	2.6694833	H	1.03662568	-3.8901404	-1.68436331
H	-1.38231047	0.08748379	2.66948712	H	2.52654137	-3.19456614	-2.33718009
H	-5.15476301	-1.83009594	-0.38481802	H	1.09942323	-2.1704237	-2.10561346
H	-6.13306912	0.34650079	-0.99905783	H	1.57505861	2.26678014	0.67365505
H	-4.75886056	2.3883111	-0.93062098	H	4.38651236	3.25769556	1.38112103
H	-1.57544127	-2.26690123	0.67388371	H	3.2972809	2.40636303	2.47731056
H	-2.16262249	-4.29443917	-0.59112919	H	2.8597671	4.00279747	1.85663574
H	-2.09815493	-2.89278476	-1.66899316	H	2.16257592	4.29456965	-0.59090069
H	-3.65796375	-3.55166707	-1.16354074	H	2.09833445	2.89311034	-1.66899854
H	-2.86023128	-4.0028558	1.8565082	H	3.65801415	3.55190241	-1.1630439

E(B3LYP/6-311G(d)) = -1160.273008 Hartree

Table S23. Cartesians coordinates of calculated IPr at the B3LYP/def2-TZVP level of theory.

Atom	X	Y	Z
N	-1.06238236	0.00005973	0.5177615
N	1.06250313	-0.00012911	0.51779671
C	0.00007768	-0.00012555	-0.34229656
C	0.67497433	0.00003542	1.85348468
C	-0.67490812	0.00016757	1.8534553
C	-2.43573062	0.00021969	0.08993117
C	-3.08504493	-1.2278654	-0.11169995
C	-4.4158182	-1.19957182	-0.52801221
C	-5.07722419	0.00056029	-0.73499246
C	-4.41540566	1.20052488	-0.52835158
C	-3.08462123	1.22847216	-0.11205655
C	-2.38103673	-2.56319841	0.07541821
C	-2.1686636	-3.26667009	-1.27418675
C	-3.11751846	-3.47472769	1.06789635
C	-2.38014082	2.5636089	0.07467969
C	-2.16690654	3.26625917	-1.27521207
C	-3.11664091	3.4759361	1.06641202
C	2.43588209	-0.00025139	0.09006559
C	3.08485908	-1.22849596	-0.1117798
C	4.41566953	-1.20049242	-0.52796577
C	5.07745459	-0.00049702	-0.73457137
C	4.41598788	1.19961391	-0.52769799
C	3.08516109	1.22785985	-0.11155528
C	2.38026923	-2.56361479	0.07471384
C	3.11734094	-3.4769228	1.06507971
C	2.1657189	-3.26519495	-1.27554894
C	2.381013	2.56313685	0.07534643
C	3.11790968	3.47544777	1.06677422
C	2.1676297	3.26580946	-1.27452716
H	1.37932686	0.00004095	2.66659287
H	-1.37928922	0.00028304	2.66654186
H	-4.94030707	-2.13127435	-0.6980707
H	-6.11015363	0.00068623	-1.06077861
H	-4.93957187	2.13236446	-0.69865116
H	-1.3942205	-2.36415777	0.49147548
H	-1.61740527	-4.19957367	-1.13481654
H	-1.60156865	-2.63343412	-1.95715435
H	-3.12208718	-3.51016094	-1.74880231
H	-2.55345257	-4.3971634	1.22274203
H	-4.10887412	-3.75315083	0.7046646
H	-3.24293731	-2.98867612	2.03734231
H	-1.39357837	2.36437822	0.49124431
H	-1.61537616	4.1990423	-1.1361182
H	-3.12002226	3.50981478	-1.75041156
H	-1.59971124	2.6324255	-1.95755107
H	-2.55220069	4.39816627	1.22110281
H	-3.24272115	2.99036525	2.03601055
H	-4.10769849	3.75468309	0.70261456
H	4.9399087	-2.13229243	-0.69822993
H	6.11042652	-0.00060242	-1.06022377
H	4.94045532	2.13132593	-0.697716
H	1.3941212	-2.36442844	0.49229285
H	2.55255871	-4.39894099	1.21978264
H	3.24473123	-2.99205498	2.03486338
H	4.10784665	-3.75604026	0.70007185

H	1.61442343	-4.19815388	-1.13667232	H	3.2446133	2.98985609	2.03627902
H	3.11843122	-3.50825103	-1.75182967	H	2.55341135	4.39758252	1.2218328
H	1.59781877	-2.63084596	-1.95681412	H	1.61642734	4.19871508	-1.13532669
H	1.3945048	2.36410565	0.49217125	H	1.60014558	2.63215279	-1.95676959
H	4.10871107	3.7544046	0.70244883	H	3.12074194	3.50915195	-1.74986158

E(B3LYP/def2-TZVP) = -1160.45385 Hartree

Table S24. Cartesians coordinates of calculated CAAC^{Me} at the B3LYP/6-311G(d) level of theory.

Atom	X	Y	Z				
N	-0.61457542	-0.03936426	0.0517062	H	1.81619155	3.16524965	-2.09154926
C	-1.33269173	-0.06136217	-1.04411497	H	0.20526009	3.88514592	-1.96276581
C	-1.35657629	-0.08152615	1.40810059	H	0.37874704	2.17706845	-2.3996444
C	2.97054865	-1.0896872	-0.04394595	C	0.7187245	2.58724041	-0.28582513
H	3.55886055	-2.00000097	-0.07956266	H	-0.33110032	2.41076406	-0.06035722
C	0.83741213	0.02908281	-0.00649198	C	1.5751686	-1.17276021	-0.05546527
C	1.47881459	1.28359544	-0.06363939	C	-1.28424704	1.28043532	2.11238704
C	2.87725331	1.30824549	-0.04939497	H	-0.25252048	1.56644631	2.32706911
H	3.39322551	2.26136884	-0.08914479	H	-1.8142077	1.22330926	3.06729483
C	-0.78382306	-1.14512196	2.34907805	H	-1.74859877	2.07423146	1.52601434
H	-0.84970643	-2.14717018	1.9253231	C	1.00573769	-2.93063222	-1.74781058
H	-1.35509413	-1.14528784	3.28166323	H	0.53327651	-2.17289978	-2.37603315
H	0.25965189	-0.94372901	2.60200505	H	0.49949268	-3.88552429	-1.92244555
C	3.62017523	0.13676838	-0.01378719	H	2.04639287	-3.03935423	-2.06802827
H	4.70484084	0.17910687	0.00217589	C	0.91679644	-2.53398892	-0.26076391
C	-2.79369586	-0.14821521	-0.61707446	H	-0.14406975	-2.4379704	-0.03169517
C	-2.77843129	-0.41976342	0.91796269	C	1.19458756	3.7429614	0.60830885
H	-3.53280044	0.16128893	1.45442283	H	1.18378725	3.47164927	1.66725496
H	-2.99424859	-1.47441905	1.11148819	H	0.542745	4.61193355	0.47900533
C	0.78557147	2.97566829	-1.77638123	H	2.20914757	4.06651289	0.36061208
				C	1.48528764	-3.63852096	0.64389031

H	2.52353914	-3.87926143	0.40026485
H	0.9081171	-4.55986841	0.52178921
H	1.45005048	-3.3579689	1.69977496
C	-3.47401602	1.18984933	-0.97318956
H	-3.03361155	2.03363549	-0.43539286
H	-3.38091739	1.39572598	-2.04166297
H	-4.53904923	1.15355315	-0.72190299
C	-3.48684557	-1.28087322	-1.39183286
H	-4.5370612	-1.36936761	-1.09286209
H	-3.44905055	-1.09200605	-2.46682628
H	-3.00376728	-2.24498362	-1.20828837

E(B3LYP/6-311G(d)) = -835.672253 Hartree

Table S25. Cartesians coordinates of calculated CAAC^{Me} at the B3LYP/def2-TZVP level of theory.

Atom	X	Y	Z
N	-0.61580509	-0.04424531	0.04491095
C	-1.33072721	-0.06736046	-1.04631703
C	-1.36151145	-0.09015527	1.39631471
C	2.96849765	-1.07247268	-0.05858971
H	3.55976246	-1.97796048	-0.09535783
C	0.83319727	0.03155679	-0.01283416
C	1.46703808	1.28687181	-0.07234237
C	2.86216975	1.31857036	-0.06423117
H	3.37089806	2.27287368	-0.10504886
C	-0.79219824	-1.15092796	2.337706
H	-0.85600871	-2.15052265	1.91224502
H	-1.36880265	-1.1486822	3.26496561
H	0.24804031	-0.94571851	2.59433283
C	3.61049865	0.15439398	-0.03309641
H	4.69239203	0.20267827	-0.02253474
C	-2.78974016	-0.15884085	-0.62972516
C	-2.77722393	-0.4327419	0.90149051
H	-3.5337939	0.14424253	1.43515611
H	-2.98630108	-1.48757981	1.08973643
C	0.80833367	3.02307017	-1.75306303
H	1.84383575	3.23377777	-2.03032296
H	0.22314031	3.93026851	-1.92311891
H	0.42878715	2.24133477	-2.41147826
C	0.70669767	2.5895485	-0.27966854
H	-0.34539195	2.40211283	-0.08536687
C	1.57685614	-1.16354291	-0.06336871
C	-1.29790624	1.2679473	2.10255997
H	-0.26970947	1.55041459	2.32980197
H	-1.83911664	1.20432677	3.04867944
H	-1.75440083	2.06056492	1.51162747
C	1.04500283	-2.95903429	-1.72663162
H	0.58316384	-2.21792454	-2.37964192
H	0.54368663	-3.91733448	-1.883892
H	2.09032311	-3.07325139	-2.02288142
C	0.93159246	-2.53020356	-0.25286497
H	-0.13079771	-2.43669172	-0.0401235
C	1.15692314	3.71993862	0.65504589
H	1.10531128	3.42397676	1.70423133
H	0.51682613	4.59451661	0.51962605
H	2.18149691	4.03526307	0.44927215
C	1.49565687	-3.61064922	0.67897399

H	2.54185834	-3.832482	0.46082281
H	0.93670721	-4.5407615	0.5541539
H	1.43022918	-3.31501401	1.72741049
C	-3.47108297	1.175987	-0.98498364
H	-3.03198346	2.01738093	-0.44607327
H	-3.37692419	1.37959947	-2.05221886
H	-4.53398125	1.13375939	-0.73319286
C	-3.47654691	-1.28987661	-1.40685796
H	-4.523936	-1.38147665	-1.10545114
H	-3.43970906	-1.09478257	-2.47918826
H	-2.98812852	-2.24966417	-1.22564621

E(B3LYP/def2-TZVP) = -835.802032 Hartree

Table S26. Cartesians coordinates of calculated DAC at the B3LYP/6-311G(d) level of theory.

Atom	X	Y	Z
N	-1.15054221	0.11552601	0.35679922
O	2.33956769	-0.39118708	2.29474309
O	-2.33952282	0.39164997	2.29472065
N	1.15053414	-0.11544266	0.35680396
C	-0.0000099	0.00001615	-0.36751758
C	1.27283309	-0.19199757	1.76419079
C	-0.00001097	-0.00000368	2.58287984
C	-1.27282241	0.19223202	1.76419698
C	0.19216244	1.24888852	3.47823008
H	0.33588364	2.15552633	2.88451393
H	1.06899141	1.11070719	4.11060583
H	-0.69157097	1.38581947	4.10104518
C	-0.19223053	-1.24925814	3.47771566
H	-0.33592016	-2.15567299	2.88364472
H	-1.06908947	-1.11134472	4.1101197
H	0.6914615	-1.38645493	4.10053714
C	-2.38885746	0.23030975	-0.41273051
C	-3.17878459	-0.9164237	-0.59712599
C	-4.34833697	-0.78630815	-1.35095555
H	-4.97836164	-1.65468699	-1.51154892
C	-4.7208596	0.43525455	-1.89542658
H	-5.63223222	0.51512091	-2.47971169
C	-3.92679124	1.55485451	-1.68890294
H	-4.22752561	2.5059029	-2.11559156
C	-2.74859256	1.4805568	-0.94011069
C	-1.91698902	2.74115786	-0.73605416
H	-1.0593322	2.48864884	-0.10940333
C	-2.71129097	3.83029102	0.0068541
H	-3.09420409	3.45868507	0.96036342
H	-3.56463571	4.18164616	-0.57980388
H	-2.07455651	4.69694768	0.20945498
C	-1.35419043	3.26029808	-2.07064709
H	-0.71600849	4.13364709	-1.90550419
H	-2.1528793	3.56039164	-2.7552468
H	-0.75866581	2.49048974	-2.56622256
C	-2.82765982	-2.27254124	0.00444721
H	-1.84254299	-2.19374229	0.4701782
C	-3.82696826	-2.6626269	1.10972824
H	-3.88356162	-1.89056018	1.879439
H	-3.52819722	-3.60216835	1.58517257
H	-4.83266709	-2.80417507	0.70336817

C	-2.72251882	-3.37199633	-1.0664501
H	-1.99966448	-3.10514499	-1.84064336
H	-3.68266365	-3.55641234	-1.55584517
H	-2.40216417	-4.31517058	-0.61348442
C	2.38882764	-0.23027248	-0.41277215
C	3.17885282	0.91640251	-0.59714346
C	4.34836624	0.78620924	-1.35101296
H	4.97848369	1.65452996	-1.51156981
C	4.72074095	-0.43535589	-1.89558228
H	5.63207537	-0.51526846	-2.47992101
C	3.92657898	-1.55488963	-1.68907865
H	4.22720748	-2.50594683	-2.11582097
C	2.74842614	-1.48053035	-0.94021498
C	2.8278841	2.27254014	0.00449675
H	1.8428774	2.19375674	0.47046608
C	2.7225002	3.37194339	-1.06642304
H	1.99922743	3.10519744	-1.84026066
H	3.68245997	3.55608459	-1.55629087
H	2.40258059	4.31522708	-0.61338483
C	3.82744652	2.66263393	1.10953591
H	3.52881662	3.60220328	1.58500307
H	4.83306501	2.80411701	0.70296984
H	3.88415519	1.89059091	1.87926803
C	1.91682891	-2.74110619	-0.73604456
H	1.05907009	-2.48850337	-0.10956326
C	1.3542071	-3.26060454	-2.07057068
H	0.75864261	-2.49097956	-2.5663774
H	0.71610277	-4.13397803	-1.905264
H	2.15296687	-3.56078626	-2.75504069
C	2.71112114	-3.83003531	0.00718787
H	3.09398373	-3.45820102	0.96062497
H	3.56450734	-4.18149003	-0.57936365
H	2.0744059	-4.69666965	0.20995918

E(B3LYP/6-311G(d)) = -1427.539553 Hartree

Table S27. Cartesians coordinates of calculated DAC at the B3LYP/def2-TZVP level of theory.

Atom	X	Y	Z
N	-1.15413762	0.0000534	0.34674682
O	2.36801019	0.00073108	2.28065227
O	-2.36803657	-0.00004787	2.28072037
N	1.15406571	0.00011875	0.34672602
C	-0.00006192	-0.00005407	-0.37096215
C	1.28398857	0.00051648	1.75018988
C	-0.00000259	0.00068916	2.56882202
C	-1.28402996	0.00017819	1.75025769
C	-0.00016746	1.26476845	3.45934976
H	-0.00030587	2.17749844	2.86075499
H	0.8905142	1.2632825	4.08493858
H	-0.89082161	1.26303142	4.0849785
C	0.00020018	-1.26252794	3.46054565
H	0.00032141	-2.17581959	2.86280799
H	-0.89045784	-1.26046109	4.08616399
H	0.89088183	-1.26019706	4.0861281
C	-2.39566857	-0.00016527	-0.42167538
C	-2.97219393	-1.22727757	-0.77485526
C	-4.15123172	-1.19913529	-1.51892576

H	-4.61892913	-2.13116783	-1.80926415
C	-4.73753926	-0.00053959	-1.89048552
H	-5.65355685	-0.00070207	-2.46812848
C	-4.15169342	1.19823443	-1.51882653
H	-4.61971337	2.13013075	-1.80908585
C	-2.9726395	1.22674303	-0.774771
C	-2.38263384	2.56939806	-0.37084145
H	-1.43907155	2.38560524	0.14266097
C	-3.30143895	3.3081532	0.61567656
H	-3.5094995	2.69594463	1.49406379
H	-4.25644938	3.5627677	0.15117911
H	-2.83491432	4.23916528	0.94652367
C	-2.05954348	3.44281229	-1.59204826
H	-1.57533119	4.37004533	-1.27768491
H	-2.96351108	3.71347345	-2.14116013
H	-1.39100428	2.92442955	-2.28028471
C	-2.381813	-2.56972286	-0.37078167
H	-1.43784602	-2.38564085	0.14187362
C	-3.29991696	-3.30794302	0.61681879
H	-3.50736031	-2.69527031	1.49502398
H	-2.83319092	-4.23880244	0.94781517
H	-4.25525456	-3.5627361	0.15308664
C	-2.05964351	-3.44384264	-1.59170929
H	-1.39173123	-2.9258305	-2.280835
H	-2.96400487	-3.71503284	-2.13990881
H	-1.57504299	-4.37080198	-1.277142
C	2.39554568	-0.00004216	-0.42179133
C	2.97226848	1.22689312	-0.77525848

C	4.15121727	1.19839248	-1.51946404
H	4.61907088	2.1302843	-1.80999964
C	4.7372202	-0.0003794	-1.89091565
H	5.65316017	-0.00050467	-2.46868167
C	4.15115903	-1.19897739	-1.51901331
H	4.61894869	-2.13100707	-1.80920615
C	2.97220901	-1.22713479	-0.77478535
C	2.38223131	2.56952552	-0.37129969
H	1.43804559	2.38574267	0.14105984
C	2.06073353	3.44387461	-1.59223483
H	1.39294594	2.9261312	-2.28168299
H	2.96535374	3.7149422	-2.1400662
H	1.57623959	4.37090637	-1.27771759
C	3.30033995	3.30734251	0.61660806
H	2.83389395	4.2383768	0.94750886
H	4.2559065	3.56176164	0.15313875
H	3.50730071	2.6945452	1.49483756
C	2.38210919	-2.56958355	-0.37030592
H	1.43799459	-2.38554677	0.14209449
C	2.06039181	-3.44432625	-1.59090134
H	1.39255267	-2.92677155	-2.28044233
H	1.57587287	-4.37121731	-1.27600651
H	2.96492056	-3.71564699	-2.13875666
C	3.30025527	-3.30714719	0.61775982
H	3.50737411	-2.69407572	1.49576155
H	4.25574825	-3.56181434	0.15427655
H	2.83375659	-4.23802351	0.94902997

E(B3LYP/def2-TZVP) = -1427.75584 Hartree

Table S28. Cartesians coordinates of calculated IPr(BIAN) at the B3LYP/6-311G(d) level of theory.

Atom	X	Y	Z
C	0.00005238	-1.42132596	0.00011377
C	-0.68493257	0.76798027	0.00958772
C	0.68501381	0.76798247	-0.00995587
C	-1.18646799	2.14155473	0.01765431
C	-2.4045513	2.78295054	0.03709008
H	-3.33871579	2.23179229	0.0521392
C	-2.42769916	4.20682871	0.03774543
H	-3.39145115	4.70567923	0.05323017
C	-1.27683202	4.9670055	0.01952151
H	-1.34136149	6.0509016	0.02052827
C	0.00004113	4.33225709	-0.00059354
C	0.00004059	2.93769161	-0.0004588
C	1.27691475	4.96699862	-0.02081219
H	1.34144884	6.05089413	-0.02203875
C	2.42778289	4.20681553	-0.03885827
H	3.39153356	4.70566613	-0.05442454
C	2.40463403	2.78294064	-0.0379054
H	3.33879155	2.23176799	-0.05281425
C	1.18654633	2.14155303	-0.01837267
C	-2.45331465	-1.00421731	0.0327107
C	-3.12961349	-1.17559763	-1.18852023
C	-4.46280603	-1.59452879	-1.14108408
H	-5.00896419	-1.74257821	-2.06687926
C	-5.09824032	-1.83307088	0.07058353
H	-6.1331703	-2.1608823	0.085393
C	-4.40805077	-1.65867118	1.26377467
H	-4.91209064	-1.85793479	2.20344251
C	-3.07472946	-1.2391008	1.27312311
C	-2.45150058	-0.96121164	-2.53651557
H	-1.45359812	-0.56056903	-2.35045504
C	-3.19758329	0.06345224	-3.40833197
H	-2.65388464	0.23538487	-4.34197325
H	-3.29698713	1.02508293	-2.89836532
H	-4.20185352	-0.2785607	-3.67442871
C	-2.2704902	-2.29945505	-3.27550537
H	-3.23392479	-2.76027102	-3.51373061
H	-1.70029433	-3.00598691	-2.66884996
H	-1.73313762	-2.14893058	-4.21683308
C	-2.32776634	-1.10123022	2.59434469
H	-1.38698497	-0.58473828	2.39454247
C	-3.09481929	-0.25794	3.62630224
H	-3.34495837	0.72974647	3.23025285
H	-2.4854311	-0.11380667	4.52321016
H	-4.02579061	-0.7368109	3.94253887
C	-1.97221036	-2.49038998	3.15577198
H	-2.8728557	-3.07032568	3.37980053
H	-1.39605194	-2.39692003	4.08153444
H	-1.37487022	-3.05629349	2.43814256
C	2.45342547	-1.00416395	-0.03249841
C	3.07494195	-1.23942626	-1.27278943
C	4.40827799	-1.6589464	-1.26319557
H	4.9124081	-1.85849776	-2.20274923
C	5.09837067	-1.83294268	-0.0698889
H	6.13331476	-2.16072082	-0.08450674
C	4.46283542	-1.59402854	1.14165262

H	5.00892336	-1.74176657	2.06753885
C	3.12963165	-1.17511467	1.18884333
C	2.3279746	-1.10212067	-2.59406546
H	1.38761192	-0.58472189	-2.3946205
C	3.09551039	-0.26039591	-3.62691983
H	3.34682285	0.72733132	-3.23171069
H	2.48591946	-0.11630981	-4.5236934
H	4.02584922	-0.74046661	-3.94319698
C	1.97128706	-2.49155237	-3.15414532
H	2.87149287	-3.07240109	-3.37757147
H	1.39521481	-2.39853269	-4.08000699
H	1.37349208	-3.05625055	-2.43594914
C	2.45136783	-0.96033947	2.53670305
H	1.45374176	-0.55909829	2.35044543
C	3.1978477	0.06381747	3.40875279
H	2.65371055	0.23645547	4.34200789
H	3.29847571	1.02525068	2.89865476
H	4.20162713	-0.27906212	3.67559126
C	2.26942486	-2.29853782	3.27556
H	3.23256784	-2.75996814	3.51379843
H	1.69882789	-3.00464583	2.66878659
H	1.73210492	-2.1477704	4.21687116
N	-1.08121585	-0.56101751	0.01457834
N	1.08130375	-0.56101873	-0.01460167

E(B3LYP/6-311G(d)) = -1543.828029 Hartree

Table S29. Cartesians coordinates of calculated IPr(BIAN) at the B3LYP/def2-TZVP level of theory.

Atom	X	Y	Z
C	-0.00005234	-1.41578783	0.00020276
C	-0.68389821	0.76384321	0.00004205
C	0.68384013	0.76382895	-0.00006174
C	-1.18469674	2.13556506	-0.00010536
C	-2.39985377	2.7756458	-0.00011238
H	-3.33285154	2.22681297	0.00002529
C	-2.42256965	4.19658883	-0.00030746
H	-3.38407383	4.694616	-0.000305
C	-1.27411414	4.95510882	-0.00049638
H	-1.33759291	6.03656306	-0.00063942
C	0.00000696	4.32107063	-0.00050832
C	-0.00000748	2.92978535	-0.00031042
C	1.27414011	4.95508253	-0.00071717
H	1.33764157	6.03653533	-0.00087301
C	2.42258048	4.19653889	-0.00072085
H	3.38409443	4.69454699	-0.00088676
C	2.3998345	2.77559778	-0.00051471
H	3.3328215	2.22674675	-0.00052464
C	1.18466463	2.13554131	-0.00030681
C	-2.44612293	-1.01046724	0.00028099
C	-3.09177605	-1.22098679	-1.22832033
C	-4.41685915	-1.65529067	-1.19980195
H	-4.93859001	-1.8336884	-2.13146737
C	-5.07554165	-1.8705877	0.00044902
H	-6.10395431	-2.21039414	0.00051411
C	-4.41671135	-1.65527459	1.20062197
H	-4.93833513	-1.83365227	2.13234808

C	-3.09162153	-1.22098867	1.22897082
C	-2.3896863	-1.02707201	-2.56377364
H	-1.41096454	-0.59162036	-2.36629607
C	-3.1426836	-0.05368377	-3.48221991
H	-2.57980909	0.10473384	-4.40470375
H	-3.28389729	0.91679689	-3.00317683
H	-4.12679191	-0.43671715	-3.76003203
C	-2.1518309	-2.37702798	-3.25851099
H	-3.0964936	-2.86990664	-3.49987506
H	-1.57328031	-3.04591886	-2.62061453
H	-1.60236706	-2.23321327	-4.19173934
C	-2.38929788	-1.02719075	2.56431688
H	-1.41094037	-0.5909398	2.36677492
C	-3.14266825	-0.0548204	3.48352472
H	-3.28504893	0.91570857	3.00492278
H	-2.57940615	0.10377229	4.40574166
H	-4.12626263	-0.43883833	3.76179673
C	-2.15031943	-2.37736081	3.25827013
H	-3.09461323	-2.8709238	3.49969884
H	-1.60061192	-2.23369093	4.19137719
H	-1.57158615	-3.0455546	2.61981382
C	2.44601645	-1.01054618	-0.00002311
C	3.09136633	-1.22159194	-1.22868853
C	4.41645181	-1.65590957	-1.20032047
H	4.93794248	-1.83468866	-2.13204955
C	5.07542208	-1.87073725	-0.00014601
H	6.10382604	-2.21056955	-0.00018427
C	4.41688042	-1.65492521	1.20009829
H	4.9387227	-1.83294706	2.13177249
C	3.09181369	-1.22058661	1.22859214
C	2.38903001	-1.02810126	-2.56406902
H	1.41023662	-0.59283474	-2.36654218
C	3.14171556	-0.05471671	-3.48278254
H	3.28286141	0.91585377	-3.00390342
H	2.57867205	0.10348426	-4.40520217
H	4.12584188	-0.43764108	-3.76068585
C	2.15137882	-2.37822056	-3.25854534
H	3.09611179	-2.87083062	-3.50019378
H	1.60154237	-2.23469875	-4.1915973
H	1.57325592	-3.04721757	-2.62037192
C	2.38987062	-1.02618726	2.56405212
H	1.41112052	-0.5908172	2.36653986
C	3.14296857	-0.05246333	3.48206382
H	2.58006052	0.10650969	4.40443281
H	3.28438594	0.9177559	3.00255197
H	4.12698984	-0.4355379	3.76013277
C	2.15213216	-2.37588624	3.25932642
H	3.09684321	-2.8686174	3.50079714
H	1.57355997	-3.04506021	2.62174926
H	1.60274239	-2.23173293	4.19254638
N	-1.0793186	-0.5613383	0.0001888
N	1.07923086	-0.56136157	0.00004494

E(B3LYP/def2-TZVP) = -1544.06310 Hartree

Table S30. Cartesians coordinates of calculated IPr(BIAN)^{CN} at the B3LYP/6-311G(d) level of theory.

Atom	X	Y	Z
C	-0.69485	-1.57145	-0.00113
C	-0.51393	0.713589	0.02647
C	0.757192	0.20117	-0.00603
C	-0.46598	2.176344	0.045193
C	-1.35773	3.223286	0.079683
H	-2.42941	3.058577	0.102848
C	-0.84875	4.553608	0.084952
H	-1.55602	5.375837	0.112163
C	0.501023	4.832648	0.056223
H	0.847528	5.860409	0.060644
C	1.444708	3.764615	0.020511
C	0.930551	2.471877	0.017299
C	2.877587	3.87251	-0.01389
C	3.65916	2.72347	-0.04784
H	4.73767	2.830073	-0.07404
C	3.102145	1.420513	-0.04937
H	3.761739	0.560666	-0.0775
C	1.731313	1.285008	-0.01586
C	-2.81647	-0.26216	0.047967
C	-3.50574	-0.12815	-1.17059
C	-4.89813	-0.00873	-1.11918
H	-5.45944	0.090853	-2.04218
C	-5.57638	-0.02468	0.092481
H	-6.65807	0.065776	0.110012
C	-4.87237	-0.16423	1.282139
H	-5.41487	-0.187	2.221072
C	-3.47971	-0.2834	1.288814
C	-2.79926	-0.14687	-2.52145
H	-1.72376	-0.18464	-2.33944
C	-3.07852	1.125175	-3.34044
H	-2.5149	1.106139	-4.2778
H	-2.78588	2.025453	-2.79397
H	-4.1371	1.221158	-3.5981
C	-3.16605	-1.4147	-3.31401
H	-4.23258	-1.44384	-3.55603
H	-2.92589	-2.31521	-2.74492
H	-2.61194	-1.45083	-4.25682
C	-2.73697	-0.48495	2.603971
H	-1.67166	-0.33967	2.413359
C	-3.1439	0.532166	3.682909
H	-3.02206	1.560479	3.332448
H	-2.52034	0.406401	4.57265
H	-4.18342	0.408143	3.998783
C	-2.91538	-1.93206	3.100092
H	-3.96659	-2.15099	3.311414
H	-2.34777	-2.09761	4.020952
H	-2.5648	-2.64425	2.35048
C	1.737289	-2.1018	-0.05369
C	2.196587	-2.56536	-1.30032
C	3.282171	-3.44601	-1.30498
H	3.655009	-3.82984	-2.24841
C	3.890267	-3.84397	-0.12092
H	4.732549	-4.52836	-0.14741

C	3.417719	-3.37197	1.096676
H	3.895284	-3.69699	2.015023
C	2.33224	-2.49268	1.159419
C	1.52006	-2.18087	-2.61061
H	0.84591	-1.34615	-2.40783
C	2.518358	-1.70588	-3.67946
H	3.137066	-0.88182	-3.31443
H	1.982221	-1.35404	-4.56552
H	3.187681	-2.50672	-4.00532
C	0.655322	-3.34521	-3.12868
H	1.267015	-4.22496	-3.35085
H	0.134808	-3.05969	-4.04794
H	-0.09336	-3.6313	-2.38733
C	1.812662	-2.02875	2.515397
H	1.033433	-1.28436	2.341867
C	2.906721	-1.35305	3.360108
H	2.48618	-0.97982	4.298393
H	3.352962	-0.50493	2.834395
H	3.712061	-2.04656	3.617423
C	1.161138	-3.19736	3.277085
H	1.88765	-3.9845	3.499537
H	0.352088	-3.64174	2.693722
H	0.744651	-2.85161	4.228039
N	-1.37732	-0.36657	0.027081
N	0.627796	-1.17976	-0.0206
C	3.499994	5.156955	-0.01393
N	3.989779	6.204286	-0.01353

E(B3LYP/6-311G(d)) = -1636.095055 Hartree

Table S31. Cartesians coordinates of calculated IPr(BIAN)^{CN} at the B3LYP/def2-TZVP level of theory.

Atom	X	Y	Z
C	-0.69572	-1.56219	0.004253
C	-0.51078	0.713274	0.003281
C	0.756772	0.197704	-0.00866
C	-0.45814	2.174134	0.004974
C	-1.34447	3.221782	0.016103
H	-2.41486	3.062174	0.027342
C	-0.83197	4.547579	0.012937
H	-1.5352	5.370534	0.021825
C	0.515928	4.82159	-0.00107
H	0.865337	5.845991	-0.00317
C	1.454007	3.752134	-0.01277
C	0.936966	2.463862	-0.00891
C	2.88449	3.854496	-0.02834
C	3.661276	2.705754	-0.03906
H	4.738009	2.809988	-0.05111
C	3.101494	1.407237	-0.0345
H	3.757951	0.547361	-0.04378
C	1.733163	1.276877	-0.01857
C	-2.81125	-0.25851	0.022175
C	-3.49671	-0.19015	-1.20076
C	-4.88667	-0.07951	-1.15938
H	-5.44404	-0.03066	-2.08603
C	-5.56775	-0.04081	0.04674

H	-6.64757	0.041878	0.056377	H	4.692278	-4.55635	-0.03509
C	-4.8679	-0.114	1.240718	C	3.347076	-3.4074	1.174472
H	-5.4112	-0.0925	2.176676	H	3.787396	-3.74825	2.102741
C	-3.47756	-0.22369	1.257332	C	2.271601	-2.51964	1.211073
C	-2.78796	-0.26912	-2.54463	C	1.605989	-2.14377	-2.57429
H	-1.71456	-0.2702	-2.35911	H	0.924117	-1.31557	-2.38233
C	-3.0947	0.943212	-3.43591	C	2.647651	-1.64595	-3.58568
H	-2.52899	0.878713	-4.36791	H	3.240271	-0.8251	-3.17769
H	-2.82485	1.878729	-2.94273	H	2.149749	-1.28593	-4.48843
H	-4.15357	0.995778	-3.69721	H	3.334829	-2.43855	-3.88743
C	-3.12113	-1.58639	-3.26295	C	0.768862	-3.29374	-3.15809
H	-4.18547	-1.6511	-3.49993	H	1.393804	-4.16361	-3.37356
H	-2.85904	-2.44482	-2.64368	H	0.292596	-2.98359	-4.09104
H	-2.56584	-1.65818	-4.20084	H	-0.01059	-3.59997	-2.45983
C	-2.74517	-0.34237	2.585222	C	1.716504	-2.06449	2.55248
H	-1.67595	-0.28174	2.385135	H	0.94848	-1.31586	2.361717
C	-3.09084	0.800603	3.550326	C	2.789237	-1.40238	3.430014
H	-2.88273	1.775121	3.104906	H	2.344658	-1.03877	4.358912
H	-2.49746	0.713675	4.462974	H	3.250736	-0.55314	2.922984
H	-4.14295	0.78307	3.841373	H	3.582211	-2.10347	3.69763
C	-3.00596	-1.71367	3.228992	C	1.038306	-3.23051	3.288675
H	-4.06439	-1.84361	3.466205	H	1.755051	-4.02061	3.523479
H	-2.44035	-1.81141	4.158426	H	0.242733	-3.66404	2.681865
H	-2.70663	-2.52041	2.559236	H	0.602456	-2.88481	4.228757
C	1.726031	-2.10581	-0.01401	N	-1.37512	-0.36125	0.010221
C	2.225062	-2.5553	-1.24708	N	0.624109	-1.17911	-0.0072
C	3.299816	-3.44379	-1.22563	C	3.511009	5.136179	-0.03295
H	3.703585	-3.81458	-2.15877	N	4.004348	6.178926	-0.03639
C	3.85788	-3.86593	-0.02909				

E(B3LYP/def2-TZVP) = -1636.340825 Hartree

Table S32. Cartesians coordinates of calculated IPr(BIAN)^{OMe} at the B3LYP/6-311G(d) level of theory.

Atom	X	Y	Z
C	1.102349	1.431768	-0.00002251
C	0.439074	-0.76571	0.00017023
C	-0.69528	0.003745	0.00016376
C	0.08415	-2.18196	0.00003683
C	0.733837	-3.39759	-0.00000814
H	1.816655	-3.46326	0.00001785
C	-0.04247	-4.58784	-0.00010746
H	0.475454	-5.54158	-0.00015321
C	-1.42386	-4.5748	-0.00014455
H	-1.98245	-5.50296	-0.00021618
C	-2.11827	-3.3345	-0.00009664
C	-1.34621	-2.17481	-0.00003054
C	-3.54298	-3.14869	-0.00012093
C	-4.07363	-1.86754	-0.00005397
H	-5.14644	-1.72368	-0.00007294
C	-3.24814	-0.7091	0.00003581
H	-3.71984	0.267888	0.00009414
C	-1.8805	-0.85	0.00003058
C	2.897335	-0.28984	0.00002095
C	3.547963	-0.48961	-1.23114995
C	4.885067	-0.89684	-1.20290169
H	5.416137	-1.04997	-2.13648819
C	5.548722	-1.10166	0.00005071
H	6.587789	-1.41655	0.00006057
C	4.885023	-0.89689	1.20298157
H	5.416051	-1.05006	2.13658512
C	3.54791	-0.48967	1.23119658
C	2.859899	-0.23846	-2.56761199
H	1.803353	-0.04941	-2.36976148
C	2.934595	-1.45651	-3.50392972
H	2.368013	-1.26561	-4.42009332
H	2.515171	-2.34891	-3.03223931
H	3.962147	-1.68703	-3.79962189
C	3.42776	1.024354	-3.24043252
H	4.490023	0.908506	-3.47647652
H	3.320012	1.893403	-2.58813681
H	2.900134	1.233673	-4.1761785
C	2.859818	-0.23859	2.56765442
H	1.803303	-0.04936	2.36979913
C	2.934311	-1.45679	3.50381038
H	2.51508	-2.34914	3.0318682
H	2.367459	-1.26605	4.41984183
H	3.961794	-1.68727	3.79975978
C	3.42781	1.024061	3.24067219
H	4.49001	0.907995	3.47689618
H	2.900064	1.233398	4.17634954
H	3.320334	1.893193	2.58843568
C	-1.16458	2.460889	0.000025
C	-1.58984	2.992063	-1.23117812
C	-2.45943	4.086466	-1.20302409
H	-2.7958	4.525197	-2.13658792
C	-2.89343	4.628655	-0.00000029
H	-3.56649	5.480624	-0.00000915

C	-2.45966	4.086316	1.20304505	C	-2.26541	2.052071	3.49739982
H	-2.79622	4.524933	2.13659404	H	-1.87959	1.595948	4.41389172
C	-1.59005	2.991931	1.23122607	H	-2.93305	1.329553	3.02082331
C	-1.10414	2.443071	-2.5670089	H	-2.86596	2.917343	3.79301158
H	-0.53192	1.535319	-2.3676786	C	-0.14953	3.439209	3.24920011
C	-2.26505	2.051968	-3.49727475	H	-0.6577	4.377123	3.49336583
H	-2.93228	1.329046	-3.02074814	H	0.697027	3.673109	2.60044571
H	-1.87915	1.596221	-4.41392001	H	0.241873	3.020879	4.18167809
H	-2.86604	2.917034	-3.7926033	N	1.513785	0.114545	0.00002159
C	-0.14947	3.439618	-3.24922531	N	-0.27704	1.325432	0.00004513
H	-0.65787	4.377397	-3.493462	O	-4.27467	-4.29172	-0.00020987
H	0.242031	3.021319	-4.18167313	C	-5.69177	-4.19788	-0.00024033
H	0.697035	3.673781	-2.60049705	H	-6.05586	-3.68135	0.89374901
C	-1.10446	2.442849	2.56706099	H	-6.05581	-3.68126	-0.89419619
H	-0.53246	1.534959	2.36774823	H	-6.05676	-5.22297	-0.00030165

E(B3LYP/6-311G(d)) = -1658.381040 Hartree

Table S33. Cartesians coordinates of calculated IPr(BIAN)^{OMe} at the B3LYP/def2-TZVP level of theory.

Atom	X	Y	Z	Atom	X	Y	Z
C	1.101694	1.418265	-0.00001655	H	-1.998	-5.48805	-0.0000307
C	0.434318	-0.76776	0.00001421	C	-2.12882	-3.3224	0.00002674
C	-0.69486	0.004674	0.00007389	C	-1.35386	-2.16778	0.00003006
C	0.073984	-2.18054	-0.00001384	C	-3.55011	-3.12971	0.00007516
C	0.71793	-3.39529	-0.0000672	C	-4.07394	-1.84916	0.00013305
H	1.798137	-3.46571	-0.00010874	H	-5.14397	-1.70346	0.00016657
C	-0.06115	-4.57998	-0.0000707	C	-3.24528	-0.69658	0.00014674
H	0.452049	-5.53346	-0.00010817	H	-3.71221	0.280205	0.00019161
C	-1.43954	-4.56246	-0.00002716	C	-1.88191	-0.84356	0.00008671
				C	2.890403	-0.29755	-0.000054

C	3.541237	-0.49315	-1.22847175	C	-1.56711	2.997204	-1.22839665
C	4.877249	-0.8925	-1.2001209	C	-2.41985	4.100638	-1.20004002
H	5.407163	-1.04373	-2.1318498	H	-2.75023	4.541872	-2.13171457
C	5.541092	-1.09224	-0.00009692	C	-2.84451	4.648429	0.00006948
H	6.579243	-1.40111	-0.00011823	H	-3.50433	5.507403	0.00010164
C	4.877276	-0.89255	1.19994301	C	-2.41964	4.100717	1.20015682
H	5.407205	-1.04383	2.13165778	H	-2.74992	4.542011	2.13183884
C	3.54125	-0.49322	1.2283345	C	-1.56689	2.997311	1.22845702
C	2.853175	-0.25447	-2.56388596	C	-1.09603	2.441036	-2.56313752
H	1.800082	-0.05862	-2.36582625	H	-0.53165	1.530719	-2.36450931
C	2.921096	-1.48274	-3.48300981	C	-2.26544	2.058347	-3.48189363
H	2.358197	-1.29728	-4.4004493	H	-2.9317	1.341226	-2.99908484
H	2.49616	-2.36433	-2.99993519	H	-1.88835	1.60228	-4.3999276
H	3.947639	-1.71973	-3.77042405	H	-2.85982	2.92872	-3.76832259
C	3.422995	0.993087	-3.25704941	C	-0.14054	3.423501	-3.25861164
H	4.481942	0.865351	-3.4935524	H	-0.64575	4.36117	-3.50193743
H	3.32146	1.871858	-2.61945025	H	0.234476	2.994755	-4.19088771
H	2.892708	1.185647	-4.19269192	H	0.713062	3.65373	-2.62035885
C	2.85321	-0.25465	2.56377682	C	-1.09559	2.441206	2.56315026
H	1.800119	-0.05875	2.36575275	H	-0.5312	1.530916	2.36444314
C	2.921103	-1.48301	3.48277456	C	-2.26485	2.058512	3.4820709
H	2.496022	-2.36451	2.99965231	H	-1.88764	1.602434	4.40005092
H	2.358321	-1.29759	4.40028885	H	-2.93121	1.341407	2.99935621
H	3.947649	-1.72014	3.77005312	H	-2.85918	2.928898	3.76858478
C	3.423048	0.992828	3.25707131	C	-0.14	3.423697	3.25845862
H	4.48192	0.86494	3.49383865	H	-0.64508	4.361481	3.50160195
H	2.892561	1.185467	4.19258211	H	0.713648	3.653678	2.62017911
H	3.321781	1.871617	2.61945311	H	0.23494	2.995077	4.19082446
C	-1.14987	2.460954	0.00000777	N	1.50982	0.105721	-0.00001844

N	-0.27317	1.321092	-0.00001747	H	-6.06501	-3.64094	0.89264477
O	-4.29062	-4.26452	0.00005189	H	-6.06509	-3.64091	-0.89234956
C	-5.70583	-4.16087	0.00012113	H	-6.08042	-5.1816	0.00012039

E(B3LYP/def2-TZVP) = -1658.634197 Hartree

Table S34. Cartesians coordinates of calculated [**1a**$^{2+}$][I^{-}]$_2$ at the B3LYP/6-311G(d) level of theory.

Atom	X	Y	Z	Atom	X	Y	Z
				H	3.27902945	1.80982401	2.03354284
N	3.68484367	1.07906892	-0.2429672	C	3.36830944	2.88655323	-1.88040091
N	3.68489051	-1.0789225	0.2433603	C	7.14241974	0.00016893	0.00032157
C	1.39552773	-0.00003207	-0.00001708	C	7.0010866	-2.36873786	0.4587176
C	3.35043726	2.46869425	-0.5341562	H	6.45376048	-3.28757235	0.63514364
C	3.12390083	3.33964031	0.54635848	C	3.16082607	-2.89607355	-2.00532605
C	4.99158448	-0.67432162	0.14417411	H	3.28158362	-1.80954063	-2.03352005
C	2.86440657	0.00002052	0.0000678	C	2.88086683	-5.12853601	1.07890231
C	3.35061336	-2.46865621	0.53417492	H	2.7000423	-6.17644726	1.29306086
C	4.99155459	0.67461559	-0.143391	C	2.8827351	4.68163482	0.23555744
C	3.36795884	-2.8867696	1.88035563	H	2.70630277	5.38929943	1.03740977
C	6.35863113	1.16856877	-0.23321593	C	3.12141931	-4.24135773	2.12181206
C	3.1248674	-3.33946921	-0.54660071	H	3.12719114	-4.60942429	3.14146176
C	0.65768941	1.00816774	-0.65181164	C	3.12150824	4.24103332	-2.12223099
H	1.15880702	1.80234101	-1.18101687	H	3.12764501	4.60890456	-3.1419475
C	6.35867172	-1.16825415	0.23388334	C	7.00100654	2.36906293	-0.45810592
C	-0.72907251	1.01004698	-0.64541118	H	6.45364925	3.28788208	-0.63451291
H	-1.24551678	1.79749806	-1.17435307	C	8.4199204	-2.38889018	0.45552434
C	3.63657423	-1.94831604	3.05602787	H	8.92350907	-3.33255875	0.63171427
H	3.76824398	-0.93460642	2.66697222	C	9.17507004	-1.25154232	0.23838795
C	2.88389791	-4.68159032	-0.23616834	H	10.25820157	-1.31377994	0.2478945
H	2.7080372	-5.38914095	-1.03825432	C	2.45281554	-1.90627123	4.04125404
C	3.1594703	2.89648854	2.00516144	H	1.52327884	-1.597638	3.55615057

H	2.65792184	-1.19519014	4.84533757	H	4.40750952	3.14305653	3.77228272
H	2.27878575	-2.87936685	4.5064677	H	4.30685608	4.59571629	2.77935346
C	8.54125328	0.00018551	0.00028291	C	2.45404592	1.90494765	-4.04114981
C	4.93555516	-2.32432897	3.79408788	H	1.52464332	1.59584026	-3.55609376
H	4.86604882	-3.3159715	4.24812511	H	2.65968918	1.19381797	-4.84505591
H	5.13414077	-1.6101332	4.59693164	H	2.27947963	2.87784313	-4.50658531
H	5.79829391	-2.32217901	3.12562608	C	4.36874031	-3.5058542	-2.74225331
C	3.63767209	1.94797057	-3.0557967	H	5.31282369	-3.24364573	-2.259085
H	3.76994977	0.93444637	-2.66647057	H	4.40835253	-3.14348188	-3.77265307
C	1.84665158	-3.2224685	-2.73688149	H	4.30629575	-4.59634387	-2.78020262
H	1.68430762	-4.30005427	-2.81823916	N	-3.72861101	-1.07240886	-0.27318695
H	1.87261006	-2.82180116	-3.75344983	N	-3.72856461	1.07226322	0.27308741
H	0.98110015	-2.7941645	-2.22575294	C	-1.45556663	-0.00009758	-0.00006067
C	2.8802155	5.12833391	-1.07960559	C	-3.32838113	-2.40218663	-0.7179324
H	2.69920387	6.17615647	-1.29404002	C	-3.099185	-2.59254951	-2.0932911
C	9.17502808	1.25192198	-0.23788318	C	-5.03958539	0.66581157	0.18315712
H	10.25815749	1.31418329	-0.24745836	C	-2.92297667	-0.00008951	-0.00004744
C	8.41983928	2.38924804	-0.45500101	C	-3.32837451	2.40205716	0.71782428
H	8.92339524	3.33292477	-0.63124027	C	-5.03962098	-0.66591771	-0.18321393
C	4.93649599	2.32458116	-3.79382398	C	-3.27808519	3.44554861	-0.22878233
H	4.86640558	3.31600111	-4.2482594	C	-6.40794669	-1.14074432	-0.3430721
H	5.13565523	1.61021308	-4.59637225	C	-3.09925471	2.59246409	2.09319529
H	5.79914822	2.3232407	-3.12525154	C	-0.72904942	-1.01022709	0.64528742
C	1.84580684	3.22441367	2.73694805	H	-1.24547701	-1.79769504	1.17421859
H	1.68492087	4.30218094	2.81878449	C	-6.40787478	1.14071727	0.34305082
H	1.87136399	2.82334504	3.75336755	C	0.65771298	-1.00828538	0.65171991
H	0.97961227	2.79745267	2.22578403	H	1.15885449	-1.80245416	1.18091061
C	4.36816235	3.50514422	2.7417712	C	-3.56747932	3.25461535	-1.71736232
H	5.31191086	3.24179733	2.25857599	H	-3.57583248	2.18088849	-1.93165882

C	-2.78397547	3.88958887	2.50950315	C	-4.95641632	3.80513305	-2.09731239
H	-2.60182084	4.08179716	3.56075997	H	-5.02123212	4.87807675	-1.89862507
C	-3.20900531	-1.47743788	-3.12869018	H	-5.14547857	3.65463791	-3.16319261
H	-3.4413013	-0.54286207	-2.61056183	H	-5.75824552	3.31364917	-1.5452542
C	-3.27805505	-3.44568917	0.22866137	C	-3.56755682	-3.25481328	1.71723011
C	-7.19149781	0.00000858	-0.00000016	H	-3.57597019	-2.18109021	1.93155868
C	-7.05081588	2.30216744	0.71965227	C	-1.88133178	1.26189969	3.8773183
H	-6.50389378	3.19499869	1.00044548	H	-1.60523186	2.1370746	4.47037245
C	-3.20900411	1.477371	3.12861709	H	-1.96780177	0.41794157	4.56674091
H	-3.44122135	0.54276084	2.61051912	H	-1.05897751	1.0539783	3.18798849
C	-2.71921493	4.9403149	1.60421088	C	-2.71892242	-4.9403657	-1.6043265
H	-2.48499797	5.94040489	1.95303278	H	-2.4845785	-5.94042584	-1.95314916
C	-2.78374849	-3.88963491	-2.5096061	C	-9.2247818	-1.21397467	-0.38591711
H	-2.60152606	-4.08181052	-3.56085725	H	-10.3079419	-1.27248111	-0.40787412
C	-2.96563608	4.71924626	0.2545002	C	-8.46983182	-2.31757799	-0.73595735
H	-2.91971267	5.55389562	-0.43510591	H	-8.97303604	-3.23106132	-1.03173341
C	-2.96544803	-4.7193467	-0.25462549	C	-4.95649673	-3.80540192	2.09706602
H	-2.91949226	-5.55400411	0.43496714	H	-5.02125831	-4.87833683	1.8983076
C	-7.05097103	-2.30215137	-0.71966608	H	-5.14562978	-3.65498174	3.16294439
H	-6.5041146	-3.19501915	-1.00047169	H	-5.7583105	-3.31391532	1.54499261
C	-8.46967565	2.31768138	0.7359705	C	-1.88134933	-1.26182991	-3.8773718
H	-8.97282082	3.23119501	1.03175293	H	-1.60512149	-2.13698797	-4.47038604
C	-9.22469782	1.21412272	0.38594671	H	-1.96790615	-0.41790182	-4.56682033
H	-10.30785411	1.27269468	0.40792226	H	-1.05904062	-1.05378626	-3.18802538
C	-2.49303634	3.89603551	-2.61638797	C	-4.36314952	-1.740831	-4.11461686
H	-1.47842714	3.58924658	-2.34884858	H	-5.31712511	-1.86145948	-3.59627236
H	-2.66356029	3.61671094	-3.65877843	H	-4.4651266	-0.90560791	-4.812151
H	-2.52433925	4.98678852	-2.56982785	H	-4.18954508	-2.64256247	-4.7068535
C	-8.59031727	0.00005304	0.00001102	C	-2.49315362	-3.8962112	2.61631955

H	-1.47853306	-3.5894119	2.34883315	H	-5.31715485	1.86123542	3.59618415
H	-2.66373921	-3.6168723	3.65869778	H	-4.46508208	0.90548254	4.81208934
H	-2.52444148	-4.98696442	2.56977869	H	-4.18964015	2.64245474	4.70674976
C	-4.36316842	1.74069568	4.11453394				

E(B3LYP/6-311G(d)) = -3318.398610 Hartree

Table S35. Cartesians coordinates of calculated [**1a**$^{•+}$][I^{-}] at the UB3LYP/6-311G(d) level of theory.

Atom	X	Y	Z	Atom	X	Y	Z
N	-3.733249	1.088627	0.247101	C	-3.415731	2.446703	0.63545
N	-3.733208	-1.088471	-0.247378	C	-3.405482	3.444735	-0.3595
C	-5.045062	0.665196	0.156437	C	-3.175465	4.759829	0.054211
C	-2.887802	0.00007	-0.000041	H	-3.160225	5.555245	-0.682999
C	-5.045036	-0.664956	-0.157264	C	-2.966682	5.067261	1.393382
C	-3.415725	-2.446624	-0.635526	H	-2.791486	6.095873	1.691431
C	-1.461498	0.000071	-0.000067	C	-2.986716	4.062076	2.352121
C	-3.217235	-2.729042	-1.999622	H	-2.828966	4.317937	3.394095
C	-0.685419	1.202524	0.002866	C	-3.217185	2.728856	1.999606
H	-1.171745	2.164636	0.005249	C	-3.405517	-3.444469	0.359612
C	-3.254394	-1.656063	-3.082206	C	-2.967105	-5.06739	-1.393009
H	-3.411726	-0.689907	-2.597626	H	-2.792102	-6.096091	-1.690865
C	0.685501	1.202474	-0.003026	C	-1.918774	-1.566669	-3.841819
H	1.171896	2.164552	-0.005607	H	-1.084201	-1.385236	-3.160766
C	-6.413722	-1.152652	-0.287476	H	-1.949229	-0.746656	-4.564635
C	-7.055753	2.332101	0.598108	H	-1.706425	-2.483752	-4.398174
H	-6.507525	3.237504	0.832868	C	-8.478093	2.351417	0.609696
C	-6.413764	1.152728	0.287095	H	-8.97812	3.282268	0.854499
C	-7.055674	-2.332103	-0.59827	C	-3.175737	-4.759669	-0.053867
H	-6.507415	-3.237452	-0.833164	H	-3.160648	-5.554968	0.683473
C	-2.986975	-4.062372	-2.35191	C	-3.615968	3.143732	-1.840782
H	-2.829246	-4.318412	-3.393841	H	-3.727505	2.062632	-1.954006
				C	-4.431498	-1.875792	-4.050482

H	-4.331678	-2.815431	-4.600715
H	-4.474756	-1.06791	-4.78608
H	-5.387544	-1.898891	-3.522099
C	-7.205729	0.000003	-0.000108
C	-3.25469	1.655701	3.082006
H	-3.412262	0.689668	2.597258
C	-8.478012	-2.351574	-0.609434
H	-8.978013	-3.282494	-0.854029
C	-3.615865	-3.143155	1.840848
H	-3.727299	-2.062026	1.953838
C	-9.236009	-1.235203	-0.319994
H	-10.319431	-1.296169	-0.337609
C	-8.600743	-0.00007	0.000089
C	-2.400416	3.565743	-2.687294
H	-2.242976	4.647298	-2.658129
H	-2.555103	3.287212	-3.733318
H	-1.480892	3.084748	-2.346172
C	-2.400235	-3.565072	2.687304
H	-2.242952	-4.646665	2.658456
H	-2.554705	-3.286214	3.733272
H	-1.480708	-3.084298	2.345881
C	-9.236052	1.23498	0.320415
H	-10.319475	1.295822	0.338361
C	-4.903685	3.796239	-2.376647
H	-5.783885	3.473489	-1.817203
H	-5.057963	3.526782	-3.425054
H	-4.853505	4.887319	-2.322481
C	-1.919189	1.565822	3.841742
H	-1.084569	1.384302	3.160764

H	-1.94993	0.7457	4.564422
H	-1.706677	2.482746	4.398295
C	-4.903569	-3.795445	2.376994
H	-5.783811	-3.472674	1.81762
H	-5.05766	-3.525805	3.425386
H	-4.853506	-4.886533	2.322964
C	-4.431826	1.875612	4.050217
H	-4.331642	2.81502	4.600778
H	-4.475513	1.067497	4.785531
H	-5.387797	1.899306	3.521734
N	3.733231	-1.088615	0.247106
N	3.733244	1.088483	-0.247372
C	5.045055	-0.665212	0.156468
C	2.887811	-0.000039	-0.000037
C	5.045061	0.664941	-0.157231
C	3.415799	2.446641	-0.635532
C	1.461507	-0.000026	-0.000069
C	3.217364	2.729063	-1.999635
C	0.685428	-1.202479	0.002856
H	1.171755	-2.164591	0.005232
C	3.254546	1.656084	-3.082218
H	3.411841	0.689925	-2.597634
C	-0.685492	-1.202429	-0.003037
H	-1.171887	-2.164506	-0.005629
C	6.413759	1.152606	-0.287427
C	7.055705	-2.332159	0.598168
H	6.507454	-3.23755	0.832923
C	6.413744	-1.152773	0.287145
C	7.05574	2.332044	-0.598215

H	6.507503	3.237404	-0.833116
C	2.987145	4.062398	-2.351932
H	2.829459	4.318441	-3.393869
C	3.41567	-2.446685	0.635442
C	3.405392	-3.444709	-0.359516
C	3.175323	-4.759797	0.054184
H	3.160058	-5.555208	-0.683032
C	2.966518	-5.067232	1.393351
H	2.791281	-6.09584	1.691391
C	2.986575	-4.062053	2.352097
H	2.828803	-4.317916	3.394067
C	3.217094	-2.728839	1.999593
C	3.405583	3.444488	0.359605
C	2.967265	5.067417	-1.393033
H	2.792296	6.096122	-1.690895
C	1.918955	1.566717	-3.841887
H	1.084351	1.385299	-3.160868
H	1.949424	0.746705	-4.564703
H	1.706647	2.483805	-4.398249
C	8.478044	-2.351505	0.609776
H	8.978048	-3.282366	0.854587
C	3.175846	4.759692	-0.053883
H	3.160751	5.554992	0.683455
C	3.615892	-3.143702	-1.840794
H	3.727495	-2.062607	-1.954006
C	4.431692	1.875791	-4.050447
H	4.331914	2.815433	-4.600682
H	4.474964	1.067911	-4.786045
H	5.387718	1.898869	-3.522027

C	7.205737	-0.000065	-0.000047
C	3.254616	-1.65569	3.081999
H	3.412283	-0.689668	2.597263
C	8.478079	2.351484	-0.60936
H	8.978102	3.282393	-0.85395
C	3.615869	3.143171	1.840848
H	3.727281	2.06204	1.953844
C	9.236048	1.235098	-0.319909
H	10.319471	1.29604	-0.33751
C	8.600751	-0.000021	0.000168
C	2.400312	-3.565625	-2.687309
H	2.242804	-4.647171	-2.658161
H	2.555014	-3.287089	-3.733329
H	1.480819	-3.084578	-2.346178
C	2.400211	3.565109	2.687255
H	2.242947	4.646704	2.658397
H	2.554634	3.286249	3.733229
H	1.48069	3.084349	2.345794
C	9.23603	-1.235084	0.320504
H	10.319452	-1.295949	0.338464
C	4.903567	-3.796284	-2.376672
H	5.783789	-3.473594	-1.817229
H	5.057856	-3.526827	-3.425078
H	4.853318	-4.887362	-2.322514
C	1.919079	-1.565718	3.84166
H	1.08451	-1.384136	3.160635
H	1.949838	-0.7456	4.564344
H	1.706469	-2.482628	4.398199
C	4.903562	3.79544	2.377046

H	5.783821	3.472657	1.817707
H	5.057606	3.525796	3.425444
H	4.853518	4.88653	2.323016
C	4.431683	-1.875688	4.050274
H	4.331403	-2.815092	4.600825
H	4.475386	-1.067579	4.785594
H	5.387683	-1.899447	3.521844

E(UB3LYP/6-311G(d)) = -3318.630162 Hartree

Table S36. Cartesians coordinates of calculated **1a** at the B3LYP/6-311G(d) level of theory (CS).

Atom	X	Y	Z
N	-0.16954854	3.74706173	-1.11864124
N	0.16949711	3.74700257	1.11865982
C	0.00016654	8.6197263	0.00007963
C	0.00004096	7.22928704	0.00007305
C	0.11078227	5.05994092	0.67122159
C	-0.23913749	9.25755994	-1.25544519
H	-0.25339099	10.34147618	-1.31756509
C	0.56764106	3.44098966	2.46591157
C	0.21028469	6.42767977	1.16599083
C	-0.11102748	5.05996546	-0.671105
C	1.93355484	3.25227505	2.75399083
C	0.23962434	9.25750945	1.25560221
H	0.25406839	10.34142194	1.31773222
C	-0.41766597	3.41563247	3.47466009
C	-0.56766743	3.44114944	-2.46592936
C	0.00001024	3.19680027	4.79050127
H	-0.73673464	3.17035454	5.586913
C	0.4439092	7.07117391	2.36245373
H	0.62102194	6.52246684	3.28076699
C	-0.21028514	6.427733	-1.16586209
C	-0.00002363	2.87111164	-0.00001012
C	-0.00002363	1.48347978	-0.00002006
C	-0.45409913	8.496661	-2.38428706
H	-0.63848467	8.99366601	-3.3314992
C	-1.90238918	3.58780492	3.17064038
H	-2.00595437	3.70599042	2.09016953
C	0.45449955	8.49655839	2.38442584
H	0.63900259	8.99351933	3.33163874
C	0.00566615	0.67924845	1.21076344
H	0.01225795	1.16307591	2.17528915
C	-1.9336017	3.25263736	-2.75408759
C	-2.29531397	3.02935607	-4.08577455
H	-3.34015956	2.87814676	-4.33644329
C	0.41766132	3.4158681	-3.47464996
C	-0.44375337	7.07127371	-2.3623279
H	-0.62091274	6.52260633	-3.2806551
C	1.34179733	3.00390452	5.09588586
H	1.64511359	2.83202405	6.12413025
C	0.00000863	3.19724688	-4.79053713
H	0.73677712	3.17083849	-5.58692655
C	2.29529234	3.02875231	4.08563373
H	3.34012737	2.8773709	4.33624232
C	-0.00569093	0.67924891	-1.21080057
H	-0.01230447	1.16308594	-2.17532083
C	3.00817657	3.28914785	1.67386455

H	2.51231845	3.44539029	0.71447281	H	4.47989387	1.98082859	0.75052546
C	-1.34178409	3.00453296	-5.09599751	H	3.07548958	1.12294438	1.39777998
H	-1.64508338	2.83285774	-6.12428123	C	2.71151701	2.34129078	-3.57358924
C	1.90238792	3.58791627	-3.17058388	H	2.33409207	1.4416483	-3.08304538
H	2.00594537	3.7059901	-2.0901001	H	3.76095583	2.46318268	-3.28885762
C	-3.00826845	3.2894416	-1.67400261	H	2.68281843	2.17209777	-4.65419933
H	-2.51246989	3.44577968	-0.71459743	C	-3.97928264	4.46543219	-1.88513311
C	3.97902795	4.46528266	1.88497576	H	-3.44976758	5.42080628	-1.91554979
H	3.44938741	5.42058529	1.91535487	H	-4.70614105	4.51048468	-1.06861509
H	4.70591035	4.51041349	1.06848229	H	-4.54013518	4.36427171	-2.81922185
H	4.53984735	4.36420349	2.81909555	N	0.16954854	-3.74706173	-1.11864124
C	2.48154106	4.85203188	-3.83083805	N	-0.16949711	-3.74700257	1.11865982
H	2.42939397	4.79645413	-4.92252853	C	-0.00016654	-8.6197263	0.00007963
H	3.53398904	4.97569774	-3.55857964	C	-0.00004096	-7.22928704	0.00007305
H	1.94859357	5.75136526	-3.51513552	C	-0.11078227	-5.05994092	0.67122159
C	-2.48143143	4.85188971	3.83104987	C	0.23913749	-9.25755994	-1.25544519
H	-2.42921382	4.79619123	4.92273245	H	0.25339099	-10.34147618	-1.31756509
H	-3.53388843	4.97565213	3.55887002	C	-0.56764106	-3.44098966	2.46591157
H	-1.94844206	5.75122048	3.51541328	C	-0.21028469	-6.42767977	1.16599083
C	-3.76310481	1.95203834	-1.57694077	C	0.11102748	-5.05996546	-0.671105
H	-4.3249508	1.73572592	-2.49073005	C	-1.93355484	-3.25227505	2.75399083
H	-4.47985761	1.98089277	-0.7507479	C	-0.23962434	-9.25750945	1.25560221
H	-3.07528522	1.12322105	-1.39792361	H	-0.25406839	-10.34142194	1.31773222
C	-2.711597	2.34119148	3.57351631	C	0.41766597	-3.41563247	3.47466009
H	-3.76101938	2.46316118	3.28875699	C	0.56766743	-3.44114944	-2.46592936
H	-2.68294943	2.17189784	4.65411114	C	-0.00001024	-3.19680027	4.79050127
H	-2.33419871	1.44157172	3.08290407	H	0.73673464	-3.17035454	5.586913
C	3.7632013	1.95186098	1.57676568	C	-0.4439092	-7.07117391	2.36245373
H	4.32515373	1.73561431	2.49050524	H	-0.62102194	-6.52246684	3.28076699

C	0.21028514	-6.427733	-1.16586209	C	-1.90238792	-3.58791627	-3.17058388
C	0.00002363	-2.87111164	-0.00001012	H	-2.00594537	-3.7059901	-2.0901001
C	0.00002363	-1.48347978	-0.00002006	C	3.00826845	-3.2894416	-1.67400261
C	0.45409913	-8.496661	-2.38428706	H	2.51246989	-3.44577968	-0.71459743
H	0.63848467	-8.99366601	-3.3314992	C	-3.97902795	-4.46528266	1.88497576
C	1.90238918	-3.58780492	3.17064038	H	-3.44938741	-5.42058529	1.91535487
H	2.00595437	-3.70599042	2.09016953	H	-4.70591035	-4.51041349	1.06848229
C	-0.45449955	-8.49655839	2.38442584	H	-4.53984735	-4.36420349	2.81909555
H	-0.63900259	-8.99351933	3.33163874	C	-2.48154106	-4.85203188	-3.83083805
C	-0.00566615	-0.67924845	1.21076344	H	-2.42939397	-4.79645413	-4.92252853
H	-0.01225795	-1.16307591	2.17528915	H	-3.53398904	-4.97569774	-3.55857964
C	1.9336017	-3.25263736	-2.75408759	H	-1.94859357	-5.75136526	-3.51513552
C	2.29531397	-3.02935607	-4.08577455	C	2.48143143	-4.85188971	3.83104987
H	3.34015956	-2.87814676	-4.33644329	H	2.42921382	-4.79619123	4.92273245
C	-0.41766132	-3.4158681	-3.47464996	H	3.53388843	-4.97565213	3.55887002
C	0.44375337	-7.07127371	-2.3623279	H	1.94844206	-5.75122048	3.51541328
H	0.62091274	-6.52260633	-3.2806551	C	3.76310481	-1.95203834	-1.57694077
C	-1.34179733	-3.00390452	5.09588586	H	4.3249508	-1.73572592	-2.49073005
H	-1.64511359	-2.83202405	6.12413025	H	4.47985761	-1.98089277	-0.7507479
C	-0.00000863	-3.19724688	-4.79053713	H	3.07528522	-1.12322105	-1.39792361
H	-0.73677712	-3.17083849	-5.58692655	C	2.711597	-2.34119148	3.57351631
C	-2.29529234	-3.02875231	4.08563373	H	3.76101938	-2.46316118	3.28875699
H	-3.34012737	-2.8773709	4.33624232	H	2.68294943	-2.17189784	4.65411114
C	0.00569093	-0.67924891	-1.21080057	H	2.33419871	-1.44157172	3.08290407
H	0.01230447	-1.16308594	-2.17532083	C	-3.7632013	-1.95186098	1.57676568
C	-3.00817657	-3.28914785	1.67386455	H	-4.32515373	-1.73561431	2.49050524
H	-2.51231845	-3.44539029	0.71447281	H	-4.47989387	-1.98082859	0.75052546
C	1.34178409	-3.00453296	-5.09599751	H	-3.07548958	-1.12294438	1.39777998
H	1.64508338	-2.83285774	-6.12428123	C	-2.71151701	-2.34129078	-3.57358924

H	-2.33409207	-1.4416483	-3.08304538	H	3.44976758	-5.42080628	-1.91554979
H	-3.76095583	-2.46318268	-3.28885762	H	4.70614105	-4.51048468	-1.06861509
H	-2.68281843	-2.17209777	-4.65419933	H	4.54013518	-4.36427171	-2.81922185
C	3.97928264	-4.46543219	-1.88513311				

E(B3LYP/6-311G(d)) = -3318.761360 Hartree

Table S37. Cartesians coordinates of calculated **1a** at the UB3LYP/6-311G(d) level of theory (T).

Atom	X	Y	Z	Atom	X	Y	Z
				C	0.61725719	8.47404258	2.35862575
N	0.24883627	3.7355967	1.0870983	H	0.86456865	8.98082802	3.2867031
N	-0.2491974	3.7355766	-1.08714907	C	1.80922683	3.63543423	-3.16235898
C	-0.00092632	8.603007	0.00009542	H	1.92707413	3.76069961	-2.08389528
C	-0.00065452	7.20259994	0.00004824	C	-0.61904368	8.47396083	-2.35844714
C	-0.16120002	5.0580034	-0.67162548	H	-0.86655799	8.98071197	-3.28648917
C	0.32222602	9.23670331	1.2346205	C	-0.00222537	0.68554304	-1.20135741
H	0.33992253	10.32072868	1.29556642	H	-0.00396804	1.17052098	-2.16432586
C	-0.65151278	3.41201066	-2.43445544	C	2.01623533	3.20357792	2.7037642
C	-0.29199027	6.4093342	-1.15573952	C	2.38319849	2.95637787	4.02998225
C	0.16096666	5.05801681	0.67151729	H	3.42735548	2.78805585	4.2713195
C	-2.01657794	3.20327039	-2.70373049	C	-0.32882405	3.40414398	3.44696364
C	-0.3243448	9.23666161	-1.23438216	C	0.60890371	7.06379744	2.34710132
H	-0.34249825	10.32068401	-1.29524662	H	0.84973954	6.51636231	3.25178148
C	0.32838467	3.40441239	-3.44706825	C	-1.43671957	2.93268586	-5.04611209
C	0.65113644	3.41203126	2.43441393	H	-1.74471683	2.74216092	-6.06947693
C	-0.09665482	3.1583704	-4.75553511	C	0.09619181	3.15809273	4.75543487
H	0.63265198	3.14278409	-5.55863351	H	-0.63316647	3.14227059	5.55848163
C	-0.61006162	7.06371854	-2.34703914	C	-2.38355932	2.95604481	-4.02994034
H	-0.8506203	6.51625107	-3.25177236	H	-3.42769249	2.78749674	-4.27122068
C	0.2910237	6.40936981	1.15577392	C	0.00214797	0.68554692	1.20132794
C	-0.00017152	2.89161889	-0.00001917	H	0.00380698	1.17053515	2.16429425
C	-0.00007026	1.46543739	-0.00001744	C	-3.08572421	3.25285578	-1.61862497

H	-2.58674634	3.41717946	-0.6617306
C	1.43629478	2.93271915	5.04608492
H	1.74427311	2.74219339	6.06945545
C	-1.809701	3.6348495	3.16218642
H	-1.92751355	3.76027701	2.08373836
C	3.08542944	3.25343615	1.6187243
H	2.58645572	3.41766603	0.66181622
C	-4.04771304	4.43519792	-1.83695236
H	-3.50970345	5.38501479	-1.87568957
H	-4.77194965	4.49217749	-1.01905136
H	-4.61096372	4.32980526	-2.76898152
C	-2.32217549	4.92343111	3.83089776
H	-2.26074924	4.86090551	4.92163746
H	-3.37058158	5.09474519	3.56980097
H	-1.75132598	5.79681412	3.51057248
C	2.32133591	4.92427284	-3.83085477
H	2.25982955	4.8619589	-4.92160039
H	3.36972453	5.09578161	-3.56981633
H	1.75030844	5.79745617	-3.51029788
C	3.85080662	1.92315989	1.50779507
H	4.42082671	1.70543117	2.41579899
H	4.56219707	1.96384945	0.67777405
H	3.17127274	1.08704118	1.32846191
C	2.66673921	2.42583307	-3.57856778
H	3.713587	2.59596283	-3.31057692
H	2.62979971	2.25564896	-4.6584935
H	2.34057901	1.50785585	-3.08453358
C	-3.85080662	1.92241107	-1.50769485
H	-4.42081724	1.70458011	-2.41567994

H	-4.56217172	1.96291497	-0.67764151
H	-3.17107759	1.08644266	-1.32841945
C	-2.66693471	2.42496175	3.57811681
H	-2.34053207	1.50715995	3.083913
H	-3.71381136	2.59489304	3.31011942
H	-2.62999732	2.25457947	4.65801122
C	4.04713072	4.43600407	1.83711613
H	3.50889283	5.38568928	1.87590759
H	4.77135998	4.49321073	1.01922405
H	4.61039774	4.3306926	2.76914419
N	-0.24883627	-3.7355967	1.0870983
N	0.2491974	-3.7355766	-1.08714907
C	0.00092632	-8.603007	0.00009542
C	0.00065452	-7.20259994	0.00004824
C	0.16120002	-5.0580034	-0.67162548
C	-0.32222602	-9.23670331	1.2346205
H	-0.33992253	-10.32072868	1.29556642
C	0.65151278	-3.41201066	-2.43445544
C	0.29199027	-6.4093342	-1.15573952
C	-0.16096666	-5.05801681	0.67151729
C	2.01657794	-3.20327039	-2.70373049
C	0.3243448	-9.23666161	-1.23438216
H	0.34249825	-10.32068401	-1.29524662
C	-0.32838467	-3.40441239	-3.44706825
C	-0.65113644	-3.41203126	2.43441393
C	0.09665482	-3.1583704	-4.75553511
H	-0.63265198	-3.14278409	-5.55863351
C	0.61006162	-7.06371854	-2.34703914
H	0.8506203	-6.51625107	-3.25177236

C	-0.2910237	-6.40936981	1.15577392	C	1.809701	-3.6348495	3.16218642
C	0.00017152	-2.89161889	-0.00001917	H	1.92751355	-3.76027701	2.08373836
C	0.00007026	-1.46543739	-0.00001744	C	-3.08542944	-3.25343615	1.6187243
C	-0.61725719	-8.47404258	2.35862575	H	-2.58645572	-3.41766603	0.66181622
H	-0.86456865	-8.98082802	3.2867031	C	4.04771304	-4.43519792	-1.83695236
C	-1.80922683	-3.63543423	-3.16235898	H	3.50970345	-5.38501479	-1.87568957
H	-1.92707413	-3.76069961	-2.08389528	H	4.77194965	-4.49217749	-1.01905136
C	0.61904368	-8.47396083	-2.35844714	H	4.61096372	-4.32980526	-2.76898152
H	0.86655799	-8.98071197	-3.28648917	C	2.32217549	-4.92343111	3.83089776
C	0.00222537	-0.68554304	-1.20135741	H	2.26074924	-4.86090551	4.92163746
H	0.00396804	-1.17052098	-2.16432586	H	3.37058158	-5.09474519	3.56980097
C	-2.01623533	-3.20357792	2.7037642	H	1.75132598	-5.79681412	3.51057248
C	-2.38319849	-2.95637787	4.02998225	C	-2.32133591	-4.92427284	-3.83085477
H	-3.42735548	-2.78805585	4.2713195	H	-2.25982955	-4.8619589	-4.92160039
C	0.32882405	-3.40414398	3.44696364	H	-3.36972453	-5.09578161	-3.56981633
C	-0.60890371	-7.06379744	2.34710132	H	-1.75030844	-5.79745617	-3.51029788
H	-0.84973954	-6.51636231	3.25178148	C	-3.85080662	-1.92315989	1.50779507
C	1.43671957	-2.93268586	-5.04611209	H	-4.42082671	-1.70543117	2.41579899
H	1.74471683	-2.74216092	-6.06947693	H	-4.56219707	-1.96384945	0.67777405
C	-0.09619181	-3.15809273	4.75543487	H	-3.17127274	-1.08704118	1.32846191
H	0.63316647	-3.14227059	5.55848163	C	-2.66673921	-2.42583307	-3.57856778
C	2.38355932	-2.95604481	-4.02994034	H	-3.713587	-2.59596283	-3.31057692
H	3.42769249	-2.78749674	-4.27122068	H	-2.62979971	-2.25564896	-4.6584935
C	-0.00214797	-0.68554692	1.20132794	H	-2.34057901	-1.50785585	-3.08453358
H	-0.00380698	-1.17053515	2.16429425	C	3.85080662	-1.92241107	-1.50769485
C	3.08572421	-3.25285578	-1.61862497	H	4.42081724	-1.70458011	-2.41567994
H	2.58674634	-3.41717946	-0.6617306	H	4.56217172	-1.96291497	-0.67764151
C	-1.43629478	-2.93271915	5.04608492	H	3.17107759	-1.08644266	-1.32841945
H	-1.74427311	-2.74219339	6.06945545	C	2.66693471	-2.42496175	3.57811681

H	2.34053207	-1.50715995	3.083913
H	3.71381136	-2.59489304	3.31011942
H	2.62999732	-2.25457947	4.65801122
C	-4.04713072	-4.43600407	1.83711613

H	-3.50889283	-5.38568928	1.87590759
H	-4.77135998	-4.49321073	1.01922405
H	-4.61039774	-4.3306926	2.76914419

E(UB3LYP/6-311G(d)) = -3318.75335 Hartree

8. References

1. El-Ayaan U, Paulovicova A and Yamada S *et al.* The crystal structure of bis[N-(2,6-diisopropylphenyl)imino] acenaphthene and studies of its copper(I) and copper(II) complexes. *J Coord Chem* 2003; **56**: 373–81.

2. Vasudevan KV, Butorac RR and Abernethy CD *et al.* Synthesis and coordination compounds of a bis(imino)acenaphthene (BIAN)-supported N-heterocyclic carbene. *Dalton Trans* 2010; **39**: 7401–8.

3. Ding L, Ying HZ and Zhou Y *et al.* Polycyclic imide derivatives: synthesis and effective tuning of lowest unoccupied molecular orbital levels through molecular engineering. *Org Lett* 2010; **12**: 5522–5.

4. Zou W and Chen C. Influence of backbone substituents on the ethylene (Co)polymerization properties of α-diimine Pd(II) and Ni(II) catalysts. *Organometallics* 2016; **35**: 1794–801.

5. Rottschäfer D, Neumann B and Stammler HG *et al.* Kekulé diradicaloids derived from a classical N-heterocyclic carbene. *Chem Sci* 2018; **9**: 4970–6.

6. Rottschäfer D, Ho NKT and Neumann B *et al.* N-Heterocyclic carbene analogues of Thiele and Chichibabin hydrocarbons. *Angew Chem Int Ed* 2018; **57**: 5838–42.

7. Dolomanov OV, Bourhis LJ and Gildea RJ *et al.* OLEX2: a complete structure solution, refinement and analysis program. *J Appl Cryst* 2009; **42**: 339–41.

8. Bourhis LJ, Dolomanov OV and Gildea RJ *et al.* The anatomy of a comprehensive constrained, restrained refinement program for the modern computing environment – Olex2 dissected. *Acta Cryst A* 2015; **71**: 59–75.

9. Sheldrick GM. Crystal structure refinement with SHELXL. *Acta Crystallogr Sect C* 2015; **71**: 3–8.

10. Stoll S and Schweiger A. EasySpin, a comprehensive software package for spectral simulation

and analysis in EPR. *J Magn Reson* 2006; **178**: 42–55.

11. Frisch MJ, Trucks GW and Schlegel HB *et al.* Gaussian, Revision E.01. Wallingford, CT: Gaussian, Inc.; 2013.

12. Hohenberg P and Kohn W. Inhomogeneous electron gas. *Phys Rev* 1964; **136**: B864–71.

13. Kohn W and Sham LJ. Self-consistent equations including exchange and correlation effects. *Phys Rev* 1965; **140**: A1133–8.

14. Lu T and Chen F. Multiwfn: a multifunctional wavefunction analyzer. *J Comput Chem* 2012; **33**: 580–92.

15. Buzzetti L, Crisenza GEM and Melchiorre P. Mechanistic studies in photocatalysis. *Angew Chem Int Ed* 2019; **58**: 3730–47.

16. Nicewicz DA and Romero NA. Organic photoredox catalysis. *Chem Rev* 2016; **116**: 10075–166.

17. Sathyanarayana P, Upare A and Ravi O *et al.* Iodine-catalyzed oxidative C–C bond cleavage for benzoic acids and benzamides from alkyl aryl ketones. *RSC Adv* 2016; **6**: 22749–53.

18. Fichez J, Prestat G and Busca P. Reductive cleavage of aromatic and heteroaromatic ester functions via copper-catalyzed proto-decarbomethoxylation. *Org Lett* 2018; **20**: 2724–7.

19. Pathania V, Roy VJ and Raha Roy S. Transforming non-innocent phenalenyl to a potent photoreductant: captivating reductive functionalization of aryl halides through visible-light-induced electron transfer processes. *J Org Chem* 2022; **87**: 16550–66.

20. Bazyar Z and Hosseini-Sarvari M. Au@ZnO core–shell: scalable photocatalytic trifluoromethylation using CF_3CO_2Na as an inexpensive reagent under visible light irradiation. *Org Process Res Dev* 2019; **23**: 2345–53.

21. Duan Z, Li W and Lei A. Nickel-catalyzed reductive cross-coupling of aryl bromides with alkyl bromides: Et_3N as the terminal reductant. *Org Lett* 2016; **18**: 4012–5.

22. Biswas A, Kolb S and Röttger SH *et al.* A BOIMPY dye enables multi-photoinduced electron transfer catalysis: reaching super-reducing properties. *Angew Chem Int Ed* 2025; **64**: e202416472.

23. Bazyar Z and Hosseini-Sarvari M. On/off O_2 switchable photocatalytic oxidative and protodecarboxylation of carboxylic acids. *J Org Chem* 2019; **84**: 13503–15.

24. Miyajima R, Ooe Y and Miura T *et al.* Triarylamine-substituted benzimidazoliums as electron

donor–acceptor dyad-type photocatalysts for reductive organic transformations. *J Am Chem Soc* 2023; **145**: 10236–48.
